# Two-Screen Virtual Board Game Didactic for Teaching Wilderness and Environmental Medicine Topics to Emergency Medicine Residents

**DOI:** 10.21980/J8J343

**Published:** 2021-10-15

**Authors:** Amy L Briggs, Robert Katzer, Isabel Algaze Gonzalez, Megan Boysen-Osborn

**Affiliations:** * University of California Irvine, Department of Emergency Medicine, Orange, CA

## Abstract

**Audience:**

This game is appropriate for medical students, interns, junior and senior residents

**Introduction:**

The COVID-19 pandemic has forced a transition from in-person to virtual learning, and educators must innovate and adapt to keep learners engaged. One way to achieve this is through gamification.^1^ The authors employed a novel approach to gamification of virtual learning which engaged not only learners’ computers but also their mobile phones. Learners worked in teams communicating by text message to answer ABEM board-style questions and occupy sites on the virtual board.

**Educational Objectives:**

By the end of this didactic, the learner will:

1. Describe the basics of the presentation of each topic listed above
2. Recall the basics of management of each topic listed above
3. Improve learners’ preparedness for the Emergency Medicine Inservice Exam and Written Board Examination

**Educational Methods:**

Wilderness and environmental medicine topics were selected from the list of topics covered on the ABEM boards. Questions were then written by the authors teaching these concepts.

**Research Methods:**

Learners were surveyed immediately following the session using an evaluation tool containing both Likert-scale questions on quality and applicability as well as open-ended questions on strengths and areas for improvement. The response rate to this survey was 100%.

**Results:**

Feedback was overwhelmingly positive, with 19/20 respondents rating the sessions 5/5 for effectiveness and value of teaching compared with other sessions, and 1/20 rating the session 4/5. Nineteen out of twenty respondents rated the content as “highly relevant”; 1/20 rated it as “mostly relevant.” Nineteen out of twenty respondents rated the session 5/5 for being engaging and holding their attention; 1/20 rated it as somewhat engaging.

**Discussion:**

Learners rated the session as highly relevant and engaging. We hypothesize that by engaging two screens and forcing learners to work together by text message, we were able to turn what would normally be a distraction (texting co-residents during resident conference) into a tool for learning.

**Topics:**

Electrical injury, lightning strike, thermal burns, inhalational injury, chemical burns, acute radiation syndrome, snake bites, scorpion envenomation, stingray envenomation, jellyfish stings, black widow spider bites, mammalian bites, rabies, murine typhus, bear encounters, decompression sickness, arterial gas embolism, drowning, dehydration, heat stroke, exercise-associated hyponatremia, frostbite, hypothermia, CO poisoning, hydrogen sulfide poisoning, giardia.

## USER GUIDE

**Table:** 

List of Resources:	
Abstract	1
User Guide	3
Wilderness Explorers The GAME	6

**Learner Audience:**

Medical students, Interns, Junior Residents, Senior Residents

**Time Required for Implementation:** 90 minutes if all 30 questions are used. Alternatively, educators can elect to use only level 1 and 2 questions, which shortens the duration of the game to approximately 60 minutes. We recommend setting a 2-minute time limit for teams to answer each question.

**Recommended number of learners per instructor:** The game is designed for 3–5 teams. We divided 20 residents into five teams of four learners each, but teams of 3–5 learners would be appropriate depending on the number of participants.

**Topics:**

Electrical injury, lightning strike, thermal burns, inhalational injury, chemical burns, acute radiation syndrome, snake bites, scorpion envenomation, stingray envenomation, jellyfish stings, black widow spider bites, mammalian bites, rabies, murine typhus, bear encounters, decompression sickness, arterial gas embolism, drowning, dehydration, heat stroke, exercise-associated hyponatremia, frostbite, hypothermia, CO poisoning, hydrogen sulfide poisoning, giardia.

**Objectives:**

By the end of this didactic, the learner will:

4. Describe the basics of the presentation of each topic listed above
5. Recall the basics of management of each topic listed above
6. Improve learners’ preparedness for the Emergency Medicine Inservice Exam and Written Board Examination

### Linked objectives and methods

Each board style question was designed to test a concept relating to either the presentation or management of the topic, addressing objectives one and two. The competitive nature of the game as well as the opportunity for other teams to “snipe” incorrectly answered questions encouraged engagement by all learners, not just those whose turn it was. The question was followed by a 1–2 slide summary lecture covering basic presentation and management to fill in any gaps not addressed by the question, rounding out objectives one and two.

### Recommended pre-reading for instructor

A review of the wilderness and environmental topics covered in this presentation using published guidelines (such as the Wilderness Medical Society guidelines), journal articles, and textbooks is advisable. One foundational text which covers many of these topics is the following:

- Tintinalli JE, Stapczynski JS, Ma OJ, Cline D, Meckler GD, Yealy h, eds. *Tintinalli’s Emergency Medicine: A Comprehensive Study Guide*. Eighth edition. McGraw-Hill Education; 2016.

The sections of this text with particular relevance are: section 2 (disaster management), chapters 8 and 10, section 13 (infectious diseases), chapters 157, 159, 150, and section 16 (environmental injuries), chapters 208 – 211, 213 – 218, and 222. Instructors are encouraged to review the references listed for any question or topic with which they are less familiar, and which can be found in the references section.

### Learner responsible content (LRC, optional)

Learners should prepare by reading the following before the game:

- Tintinalli JE, Stapczynski JS, Ma OJ, Cline D, Meckler GD, Yealy DM, eds. *Tintinalli’s Emergency Medicine: A Comprehensive Study Guide*. Eighth edition. McGraw-Hill Education; 2016.
- Section 2 (disaster management), chapters 8 and 10, section 13 (infectious diseases), chapters 157, 159, 150, and section 16 (environmental injuries), chapters 208 – 211, 213 – 218, and 222.

### Results and tips for successful implementation

The exercise was presented as part of a weekly emergency medicine grand rounds series for residents and medical students. Twenty learners participated and gave feedback. Feedback was overwhelmingly positive, with 19/20 respondents rating the sessions 5/5 for effectiveness and value of teaching compared with other sessions, and 1/20 rating the session 4/5. Nineteen out of twenty respondents rated the content as “highly relevant”; 1/20 rated it as “mostly relevant.” Nineteen out of twenty respondents rated the session 5/5 for being engaging and holding their attention; 1/20 rated it as somewhat engaging. Some modifications have been made for publication, primarily the removal of copyrighted images.

For successful implementation, it is vital that teams are able to communicate effectively within the team. Establishing teams ahead of time and ensuring that this asynchronous communication channel is functional is recommended.

### Technology necessary

Participants must be able to log into a video meeting platform such as Zoom or WebEx (typically from a laptop with a webcam) as well as an alternate channel of communication for individual teams, such as a group text message thread or a WhatsApp group. The presenter must have a PowerPoint or similar slide presentation software as well as the ability to be on presenter mode in the video chat platform. There must also be a text chat function in the video meeting platform.

### Assessment (optional)

Learners were not formally tested or evaluated on the material following this session. However, all participants indicated in their responses that they found the session to have high relevance and teaching value.

## Supplementary Information



## Figures and Tables

**Figure:**
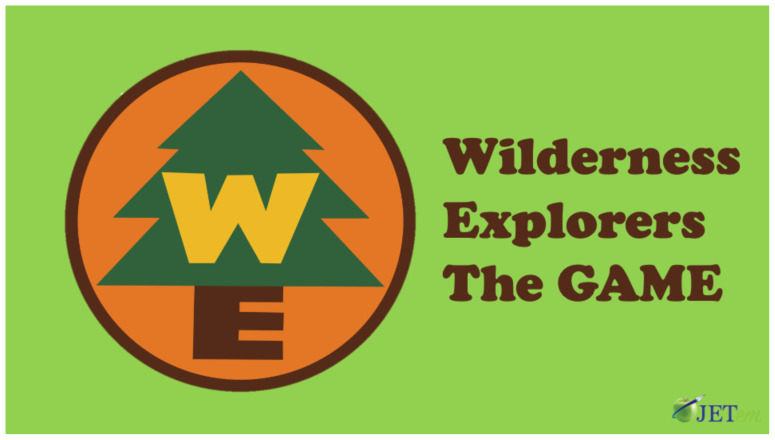
Please see associated video file
