# Supplementary material for: Two-Screen Virtual Board Game Didactic for Teaching Wilderness and Environmental Medicine Topics to Emergency Medicine Residents
Source: J Educ Teach Emerg Med. 2021 Oct 15;6(4):L1–6. doi: 10.21980/J8J343 (PMC10332738; doi:10.21980/J8J343)
Supplement: Supplementary file 1 [file JETem-6-4-L1-supp1.pptx]

## Slide 1
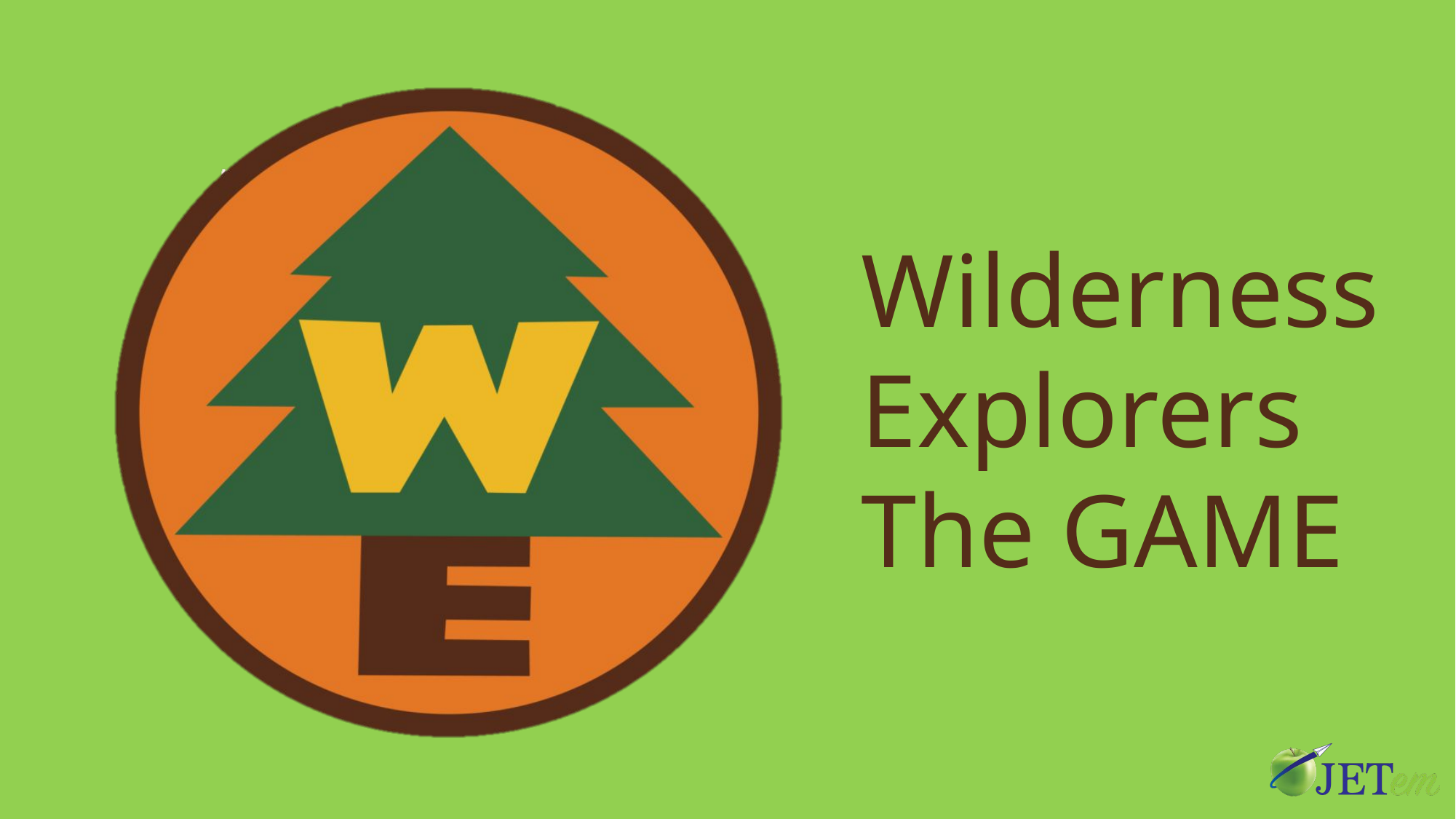

Wilderness
Explorers
The GAME

## Slide 2
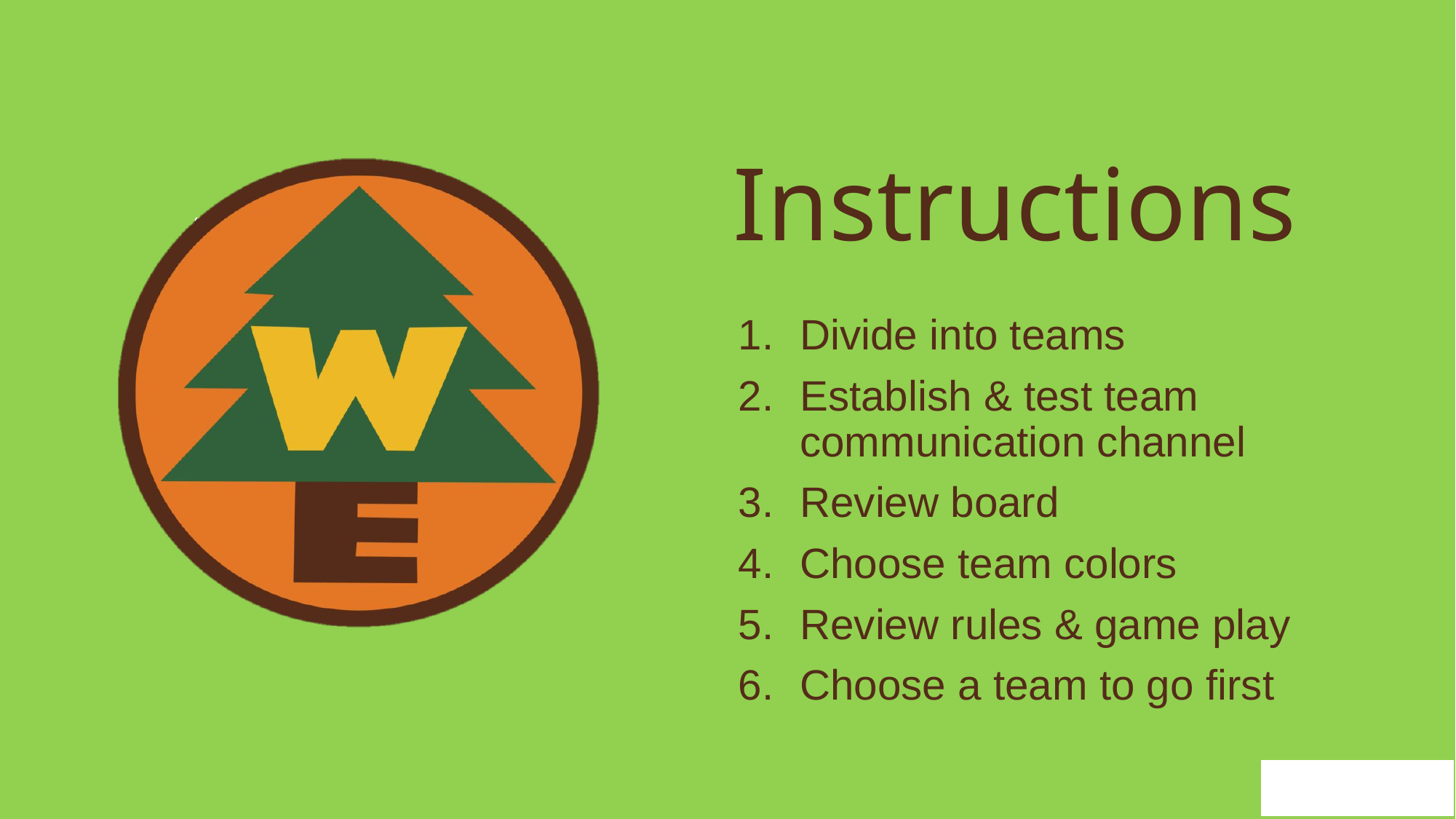

Instructions
Divide into teams
Establish & test team communication channel
Review board
Choose team colors
Review rules & game play
Choose a team to go first

## Slide 3
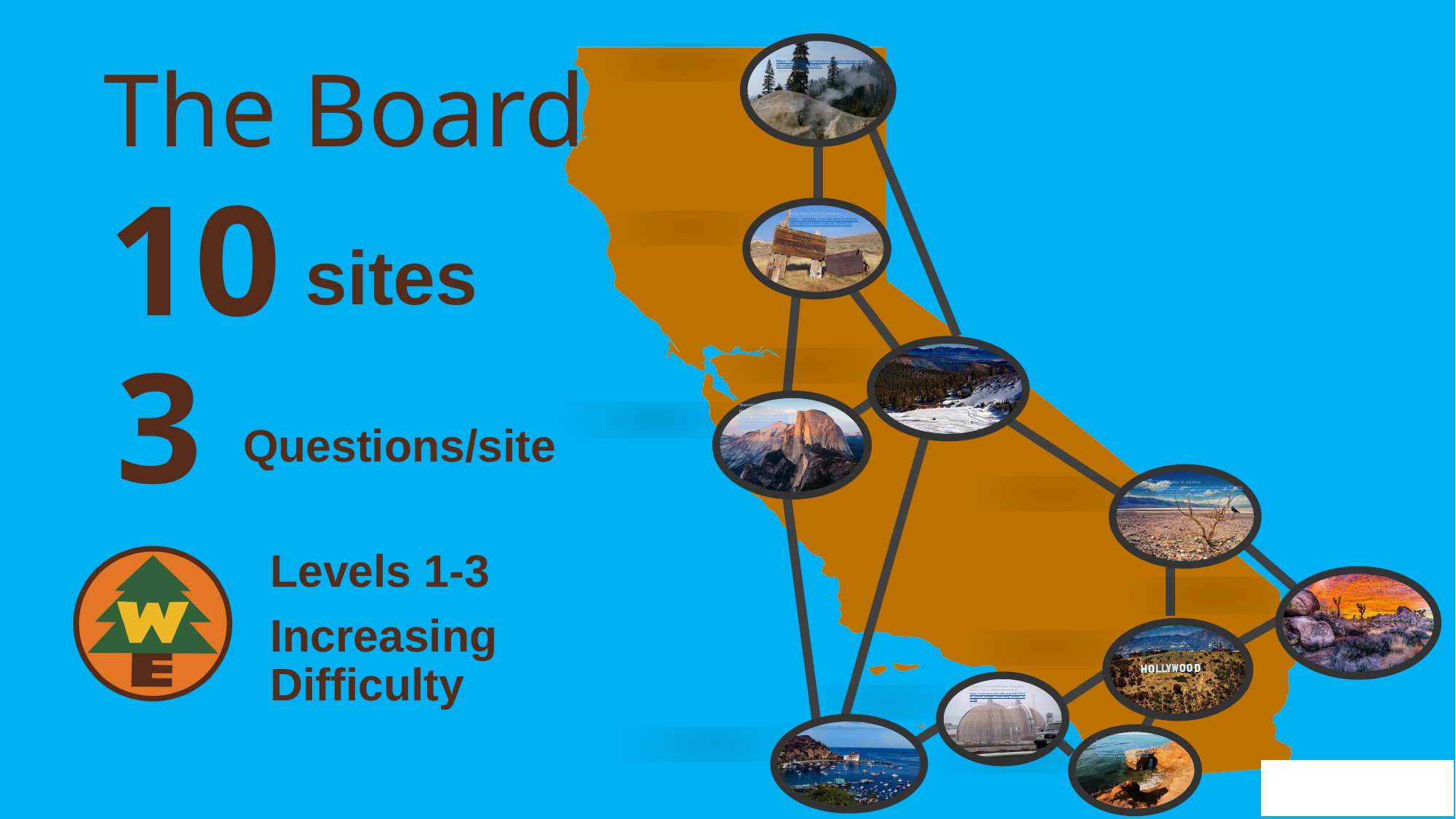

Lassen Volcano National Park. In:pixabay. https://pixabay.com/photos/volcano-lassen-volcano-national-park-57487/. Accessed May 5, 2021. CC0.
The Board
10
Bodie State Park. In: pixabay. https://pixabay.com/photos/outdoors-abandoned-farm-nature-3344219/. Accessed May 8, 2021. CC0.
sites
Mammoth Lakes. In: pixabay. https://pixabay.com/photos/mammoth-lakes-california-winter-1594375/. Accessed May 8, 2021. CC0
3
Yosemite National Park. In: pixabay. https://pixabay.com/photos/half-dome-yosemite-national-park-768806. Accessed May 8, 2021. CC0..
Questions/site
Death Valley. In: pixabay. https://pixabay.com/photos/death-valley-landscape-bush-broom-4254872/. Accessed May 8, 2021. CC0
Levels 1-3
Increasing Difficulty
Joshua Tree. In: pixabay. https://pixabay.com/photos/joshua-tree-national-park-california-4701762/. Accessed May 8, 2021.CC0
Los Angeles. In: pixabay.https://pixabay.com/photos/hollywood-sign-los-angeles-hollywood-1598473/. Accessed May 8, 2021. CC0
Dacanay, R. San Onofre Nuclear Generating Station, 2001. In: Wikimedia commons. https://commons.wikimedia.org/wiki/File:San_Onofre_Nuclear_Generating_Station,_2001.jpg. Published December 30, 2001. Accessed May 8, 2021. CC-BY 2.0.
Catalina Island. In: pixabay. https://pixabay.com/photos/catalina-island-california-landscape-1587272/. Accessed May 8, 2021. CC0.
La Jolla. In: pixabay. https://pixabay.com/photos/la-jolla-la-jolla-cove-san-diego-4798031/. Accessed May 8, 2021. CC0

## Slide 4
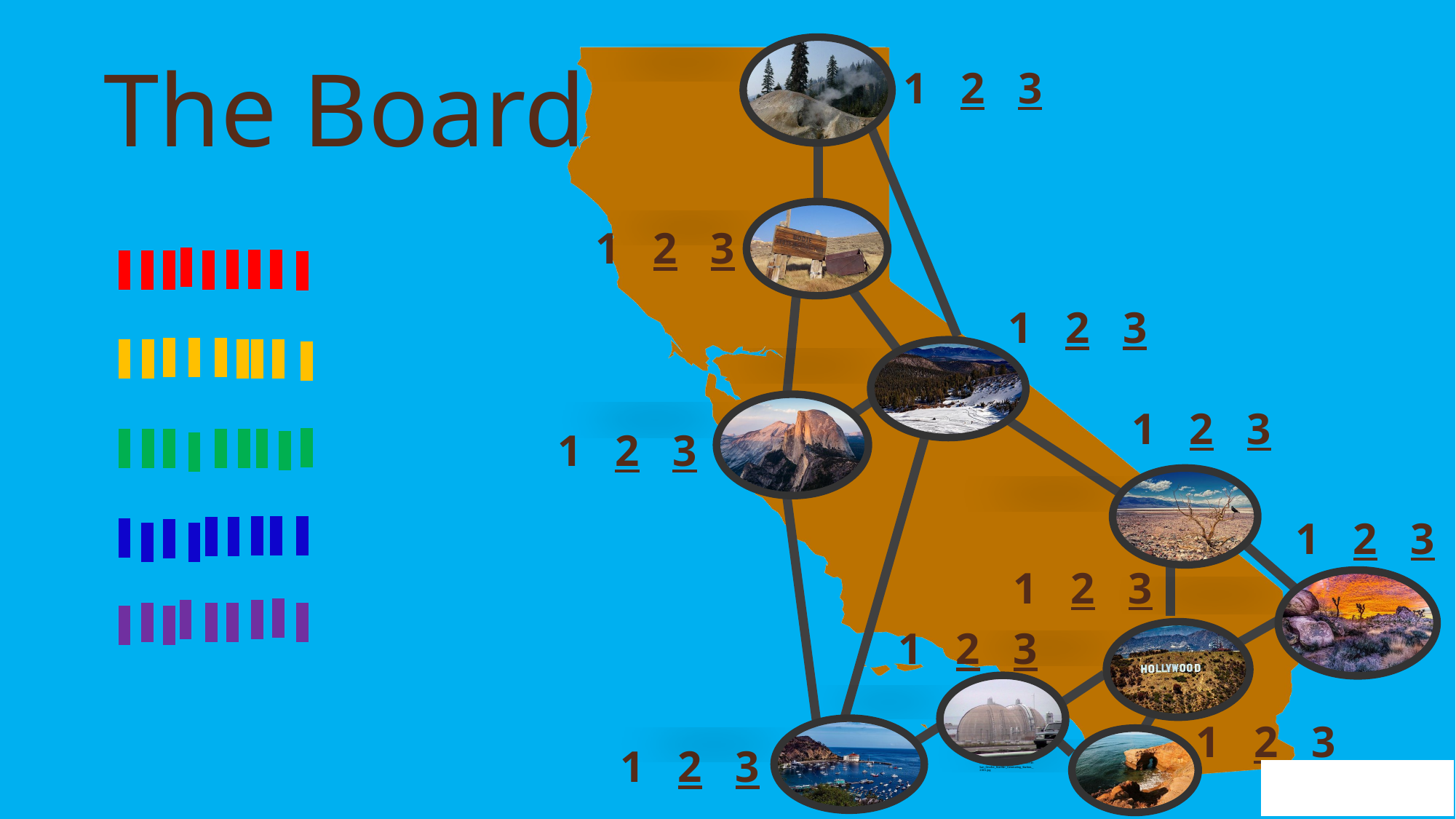

The Board
1 2 3
1 2 3
1 2 3
1 2 3
1 2 3
1 2 3
1 2 3
1 2 3
Credit: Radcliffe Dacanay https://commons.wikimedia.org/wiki/File:San_Onofre_Nuclear_Generating_Station,_2001.jpg
1 2 3
1 2 3

## Slide 5
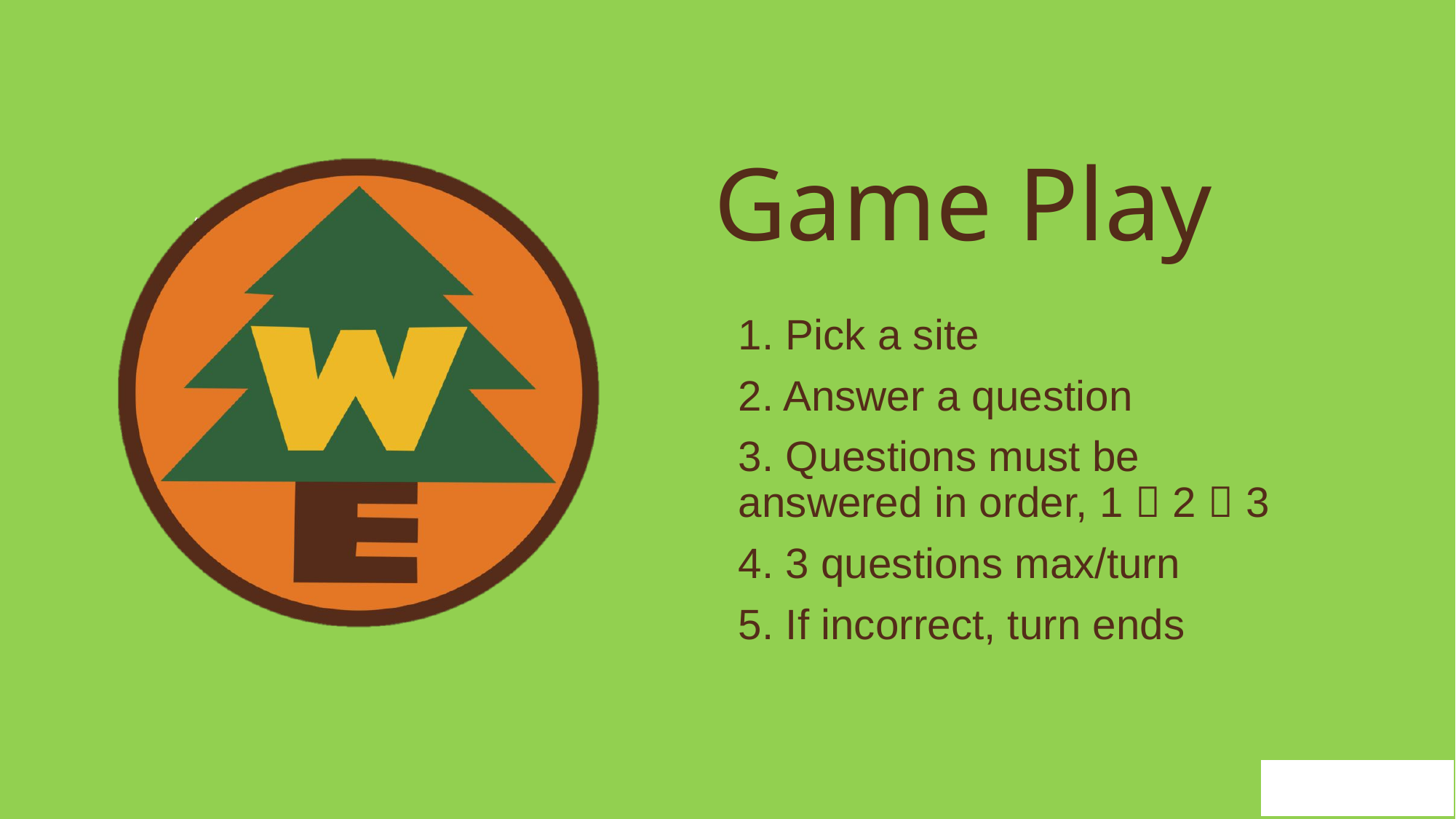

Game Play
1. Pick a site​
2. Answer a question
3. Questions must be answered in order, 1  2  3​
4. 3 questions max/turn​
5. If incorrect, turn ends​

## Slide 6
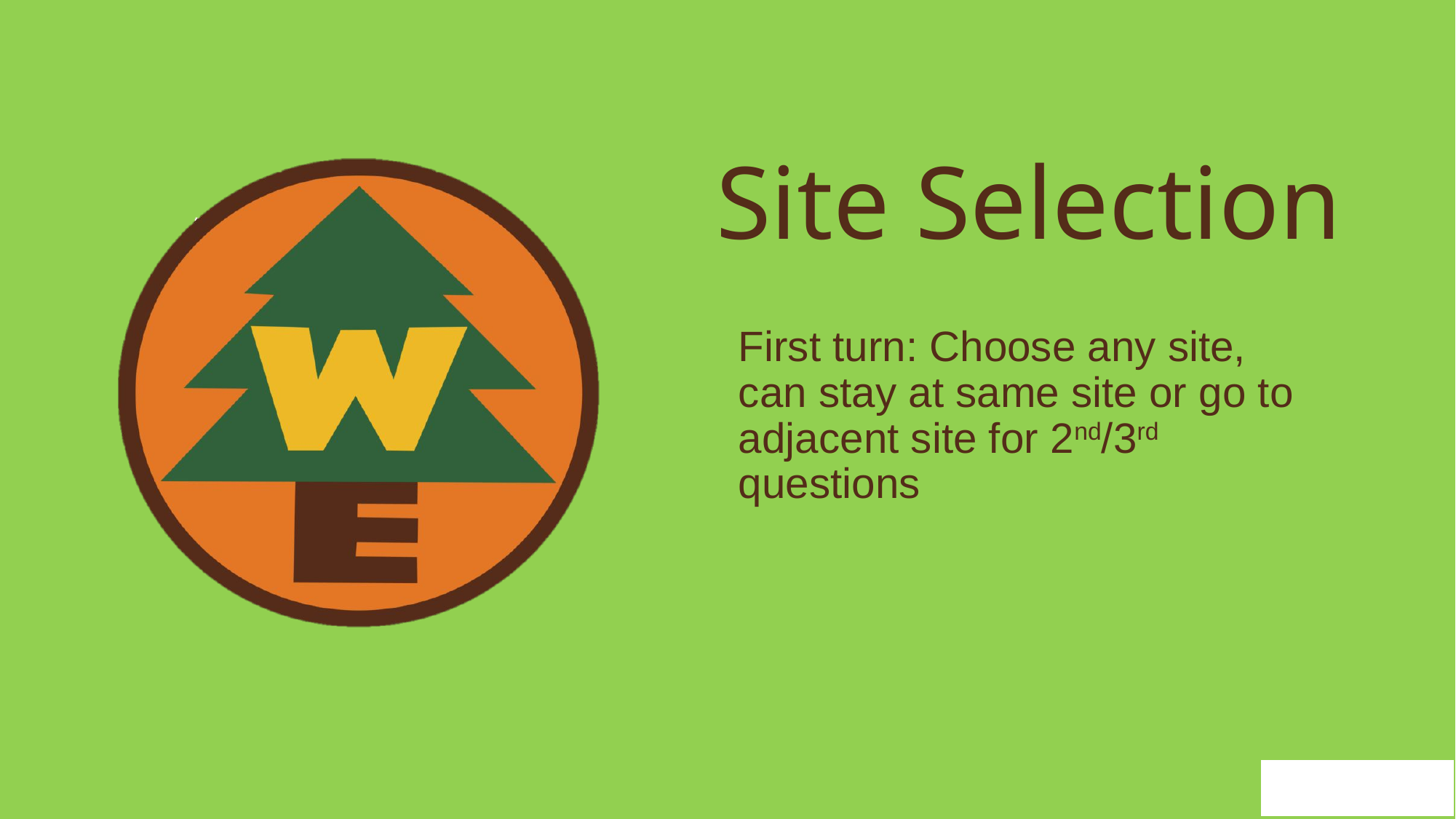

Site Selection
First turn: Choose any site, can stay at same site or go to adjacent site for 2nd/3rd questions

## Slide 7
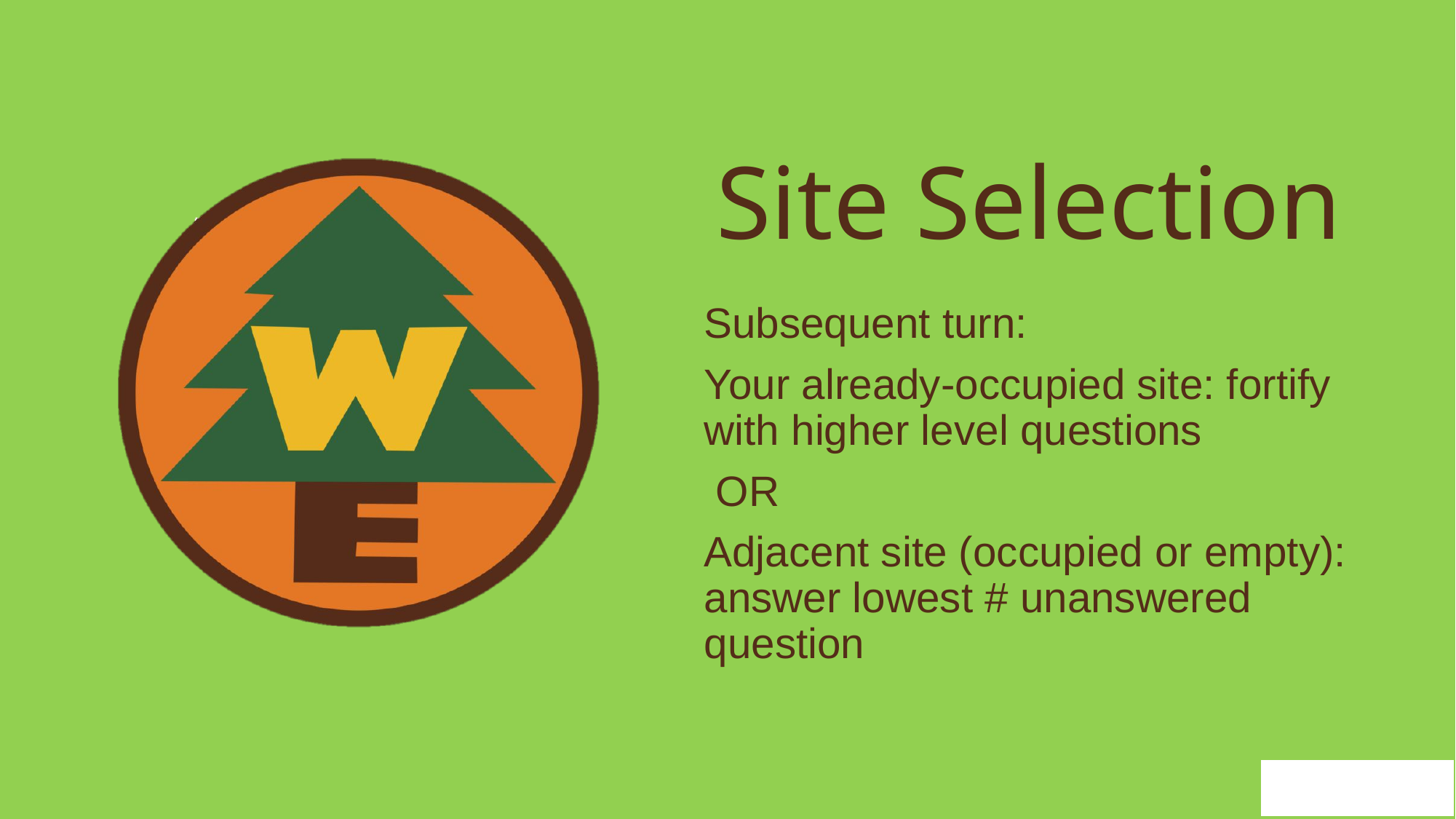

Site Selection
Subsequent turn:
Your already-occupied site: fortify with higher level questions​
 OR
Adjacent site (occupied or empty): answer lowest # unanswered question

## Slide 8
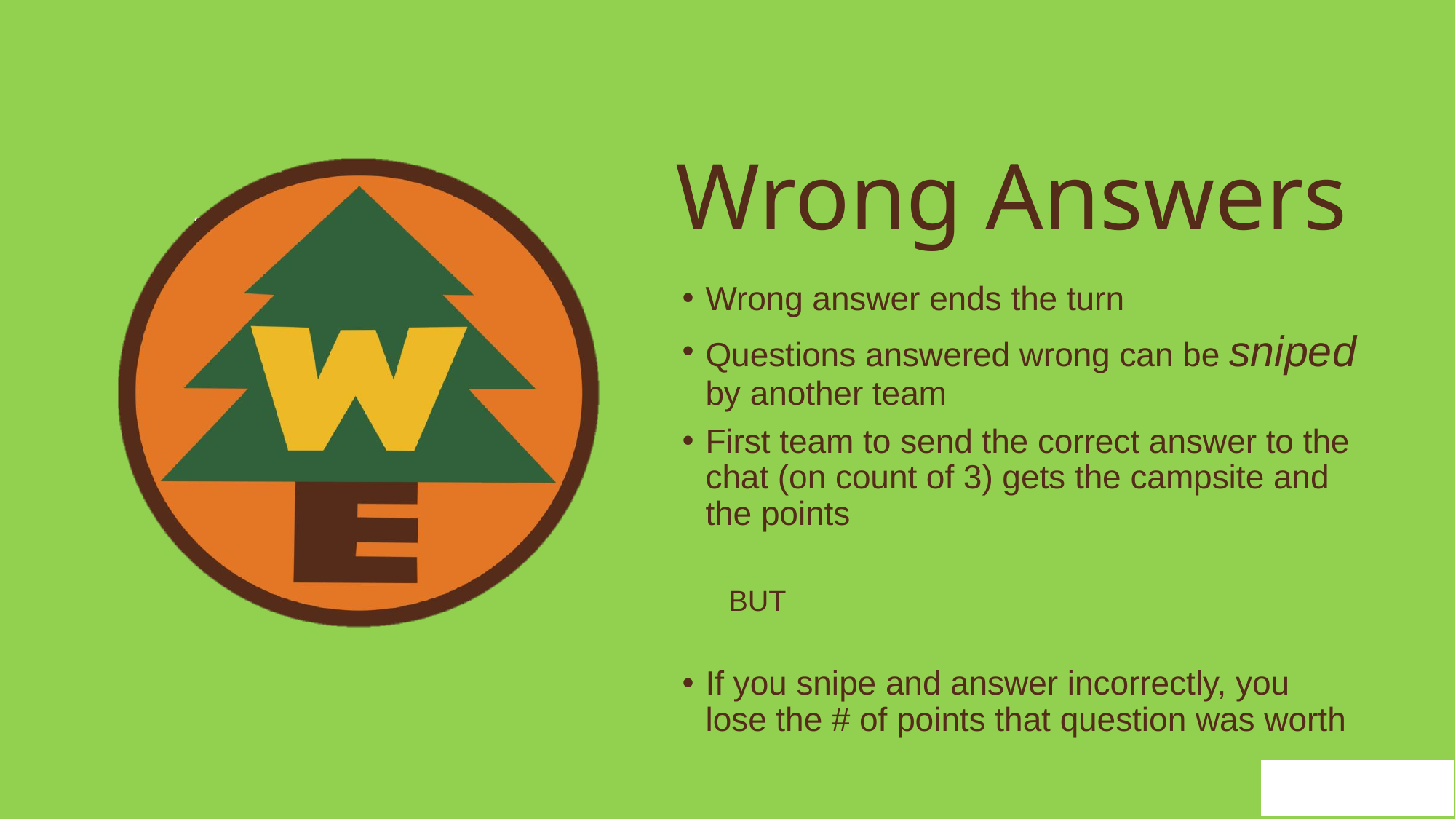

Wrong Answers
Wrong answer ends the turn
Questions answered wrong can be sniped by another team
First team to send the correct answer to the chat (on count of 3) gets the campsite and the points
BUT
If you snipe and answer incorrectly, you lose the # of points that question was worth

## Slide 9
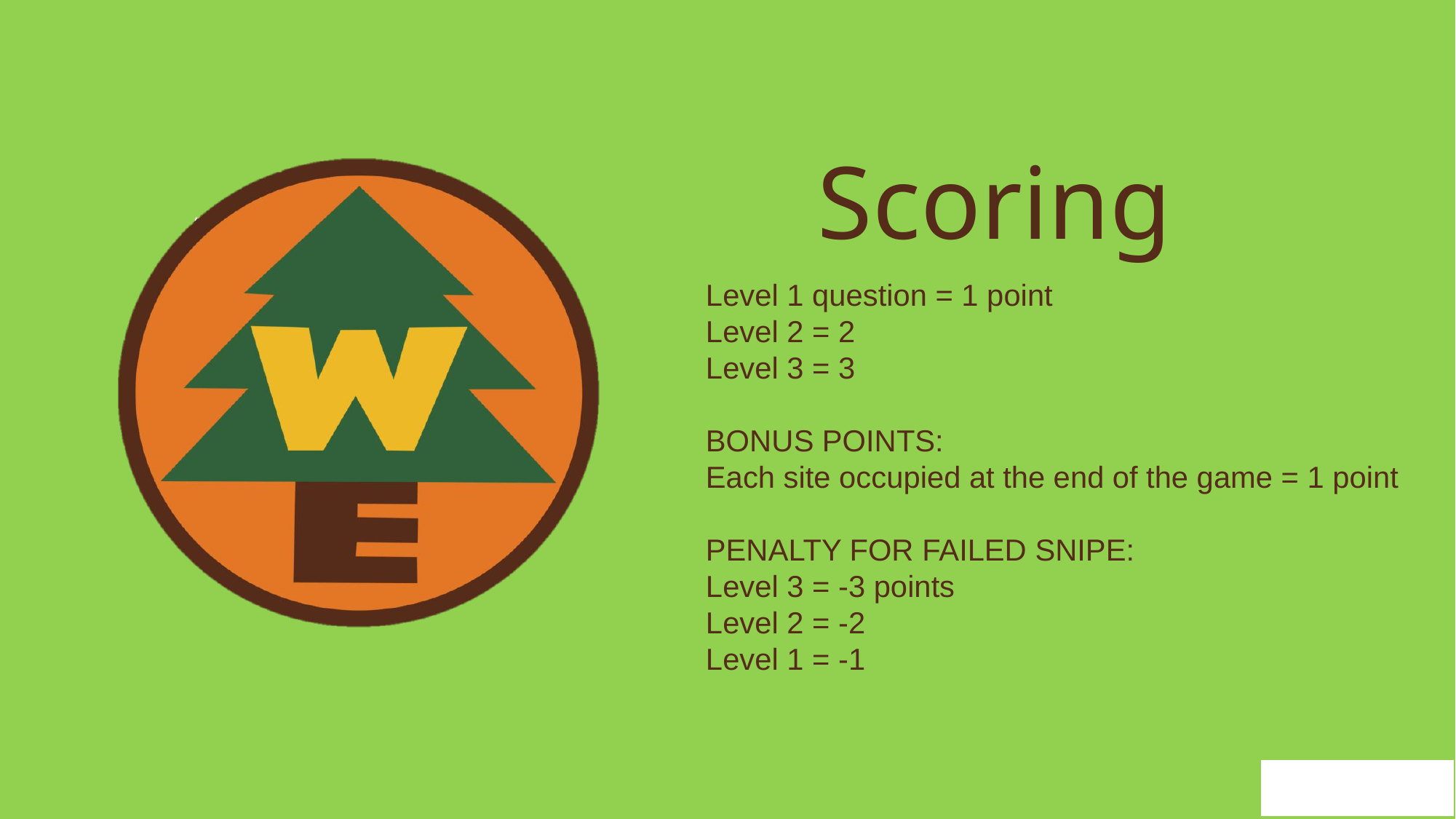

Scoring
Level 1 question = 1 point
Level 2 = 2
Level 3 = 3
BONUS POINTS:
Each site occupied at the end of the game = 1 point
PENALTY FOR FAILED SNIPE:
Level 3 = -3 points
Level 2 = -2
Level 1 = -1

## Slide 10
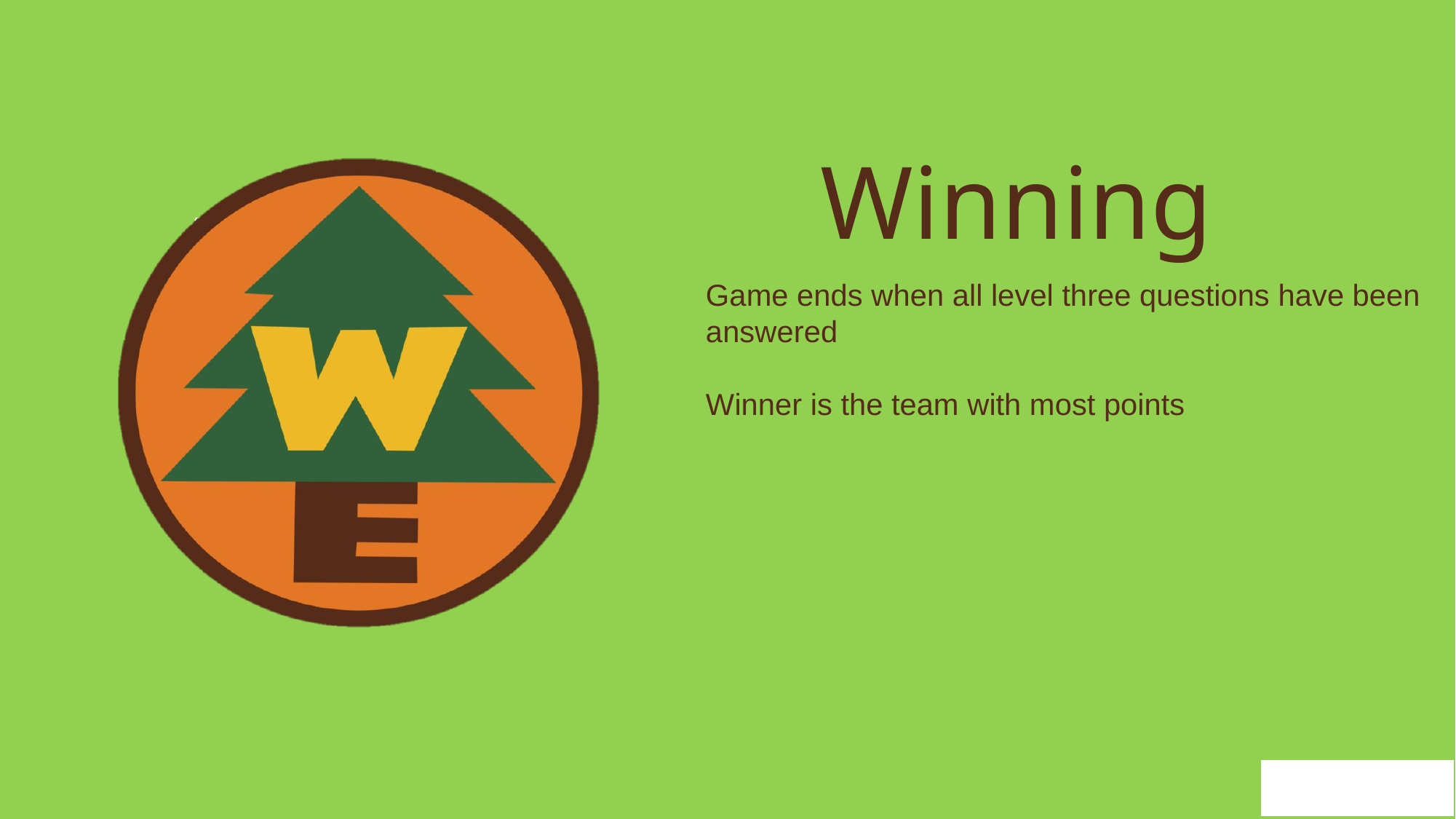

Winning
Game ends when all level three questions have been answered
Winner is the team with most points

## Slide 11
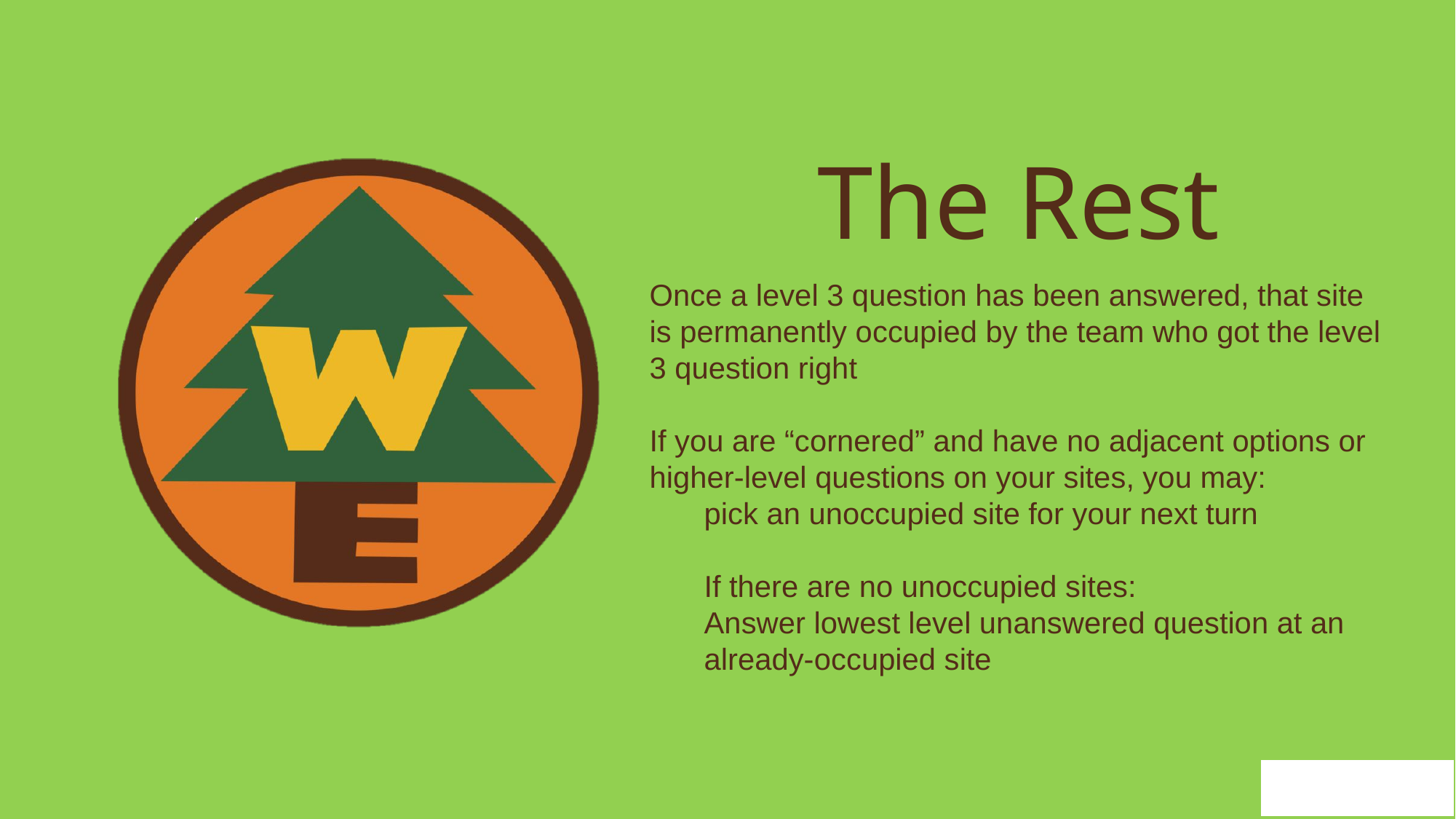

The Rest
Once a level 3 question has been answered, that site is permanently occupied by the team who got the level 3 question right
If you are “cornered” and have no adjacent options or higher-level questions on your sites, you may:
pick an unoccupied site for your next turn
If there are no unoccupied sites:
Answer lowest level unanswered question at an already-occupied site

## Slide 12
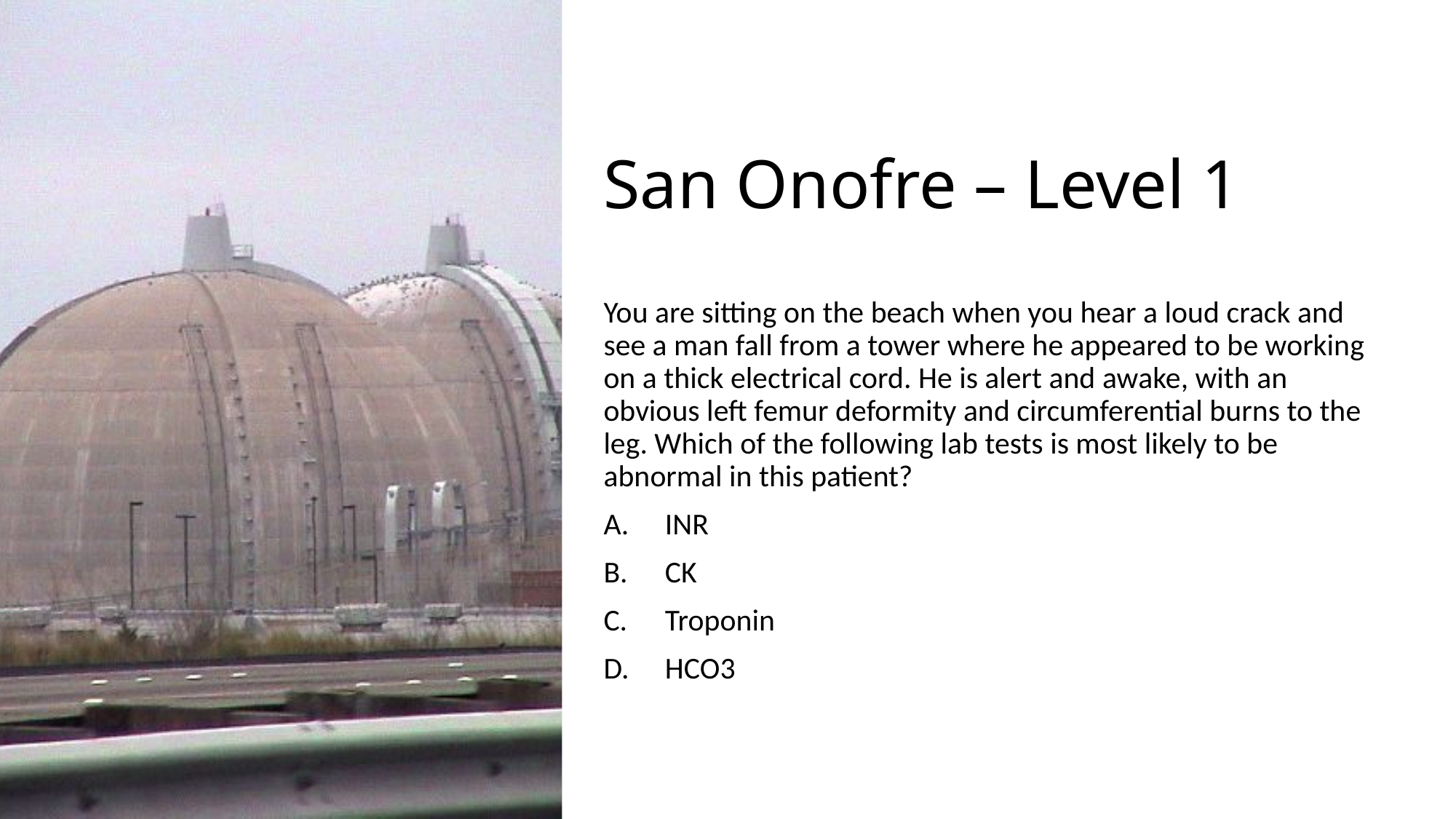

# San Onofre – Level 1
You are sitting on the beach when you hear a loud crack and see a man fall from a tower where he appeared to be working on a thick electrical cord. He is alert and awake, with an obvious left femur deformity and circumferential burns to the leg. Which of the following lab tests is most likely to be abnormal in this patient?
INR
CK
Troponin
HCO3

## Slide 13
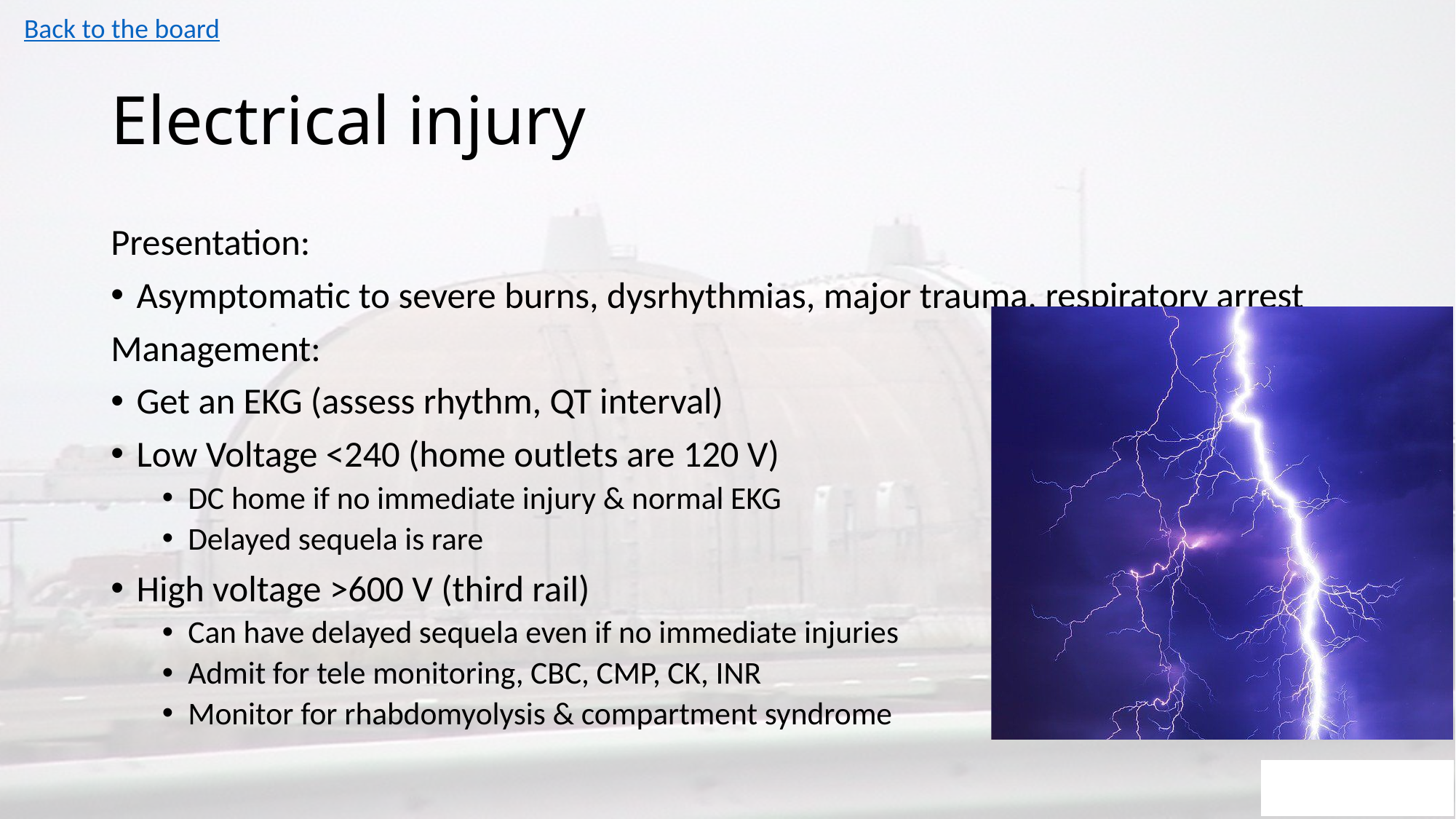

Back to the board
# Electrical injury
Presentation:
Asymptomatic to severe burns, dysrhythmias, major trauma, respiratory arrest
Management:
Get an EKG (assess rhythm, QT interval)
Low Voltage <240 (home outlets are 120 V)
DC home if no immediate injury & normal EKG
Delayed sequela is rare
High voltage >600 V (third rail)
Can have delayed sequela even if no immediate injuries
Admit for tele monitoring, CBC, CMP, CK, INR
Monitor for rhabdomyolysis & compartment syndrome

## Slide 14
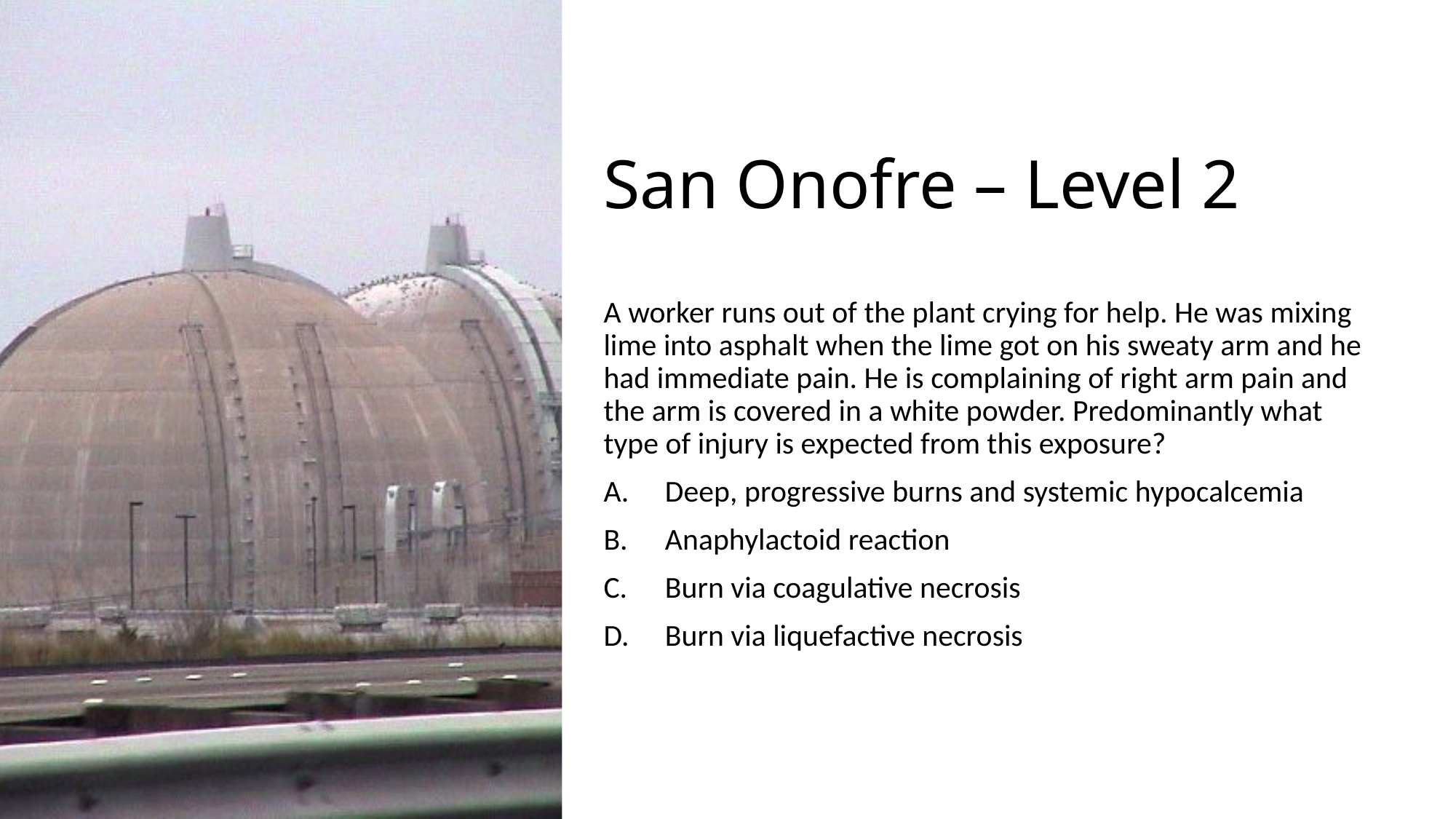

# San Onofre – Level 2
A worker runs out of the plant crying for help. He was mixing lime into asphalt when the lime got on his sweaty arm and he had immediate pain. He is complaining of right arm pain and the arm is covered in a white powder. Predominantly what type of injury is expected from this exposure?
Deep, progressive burns and systemic hypocalcemia
Anaphylactoid reaction
Burn via coagulative necrosis
Burn via liquefactive necrosis

## Slide 15
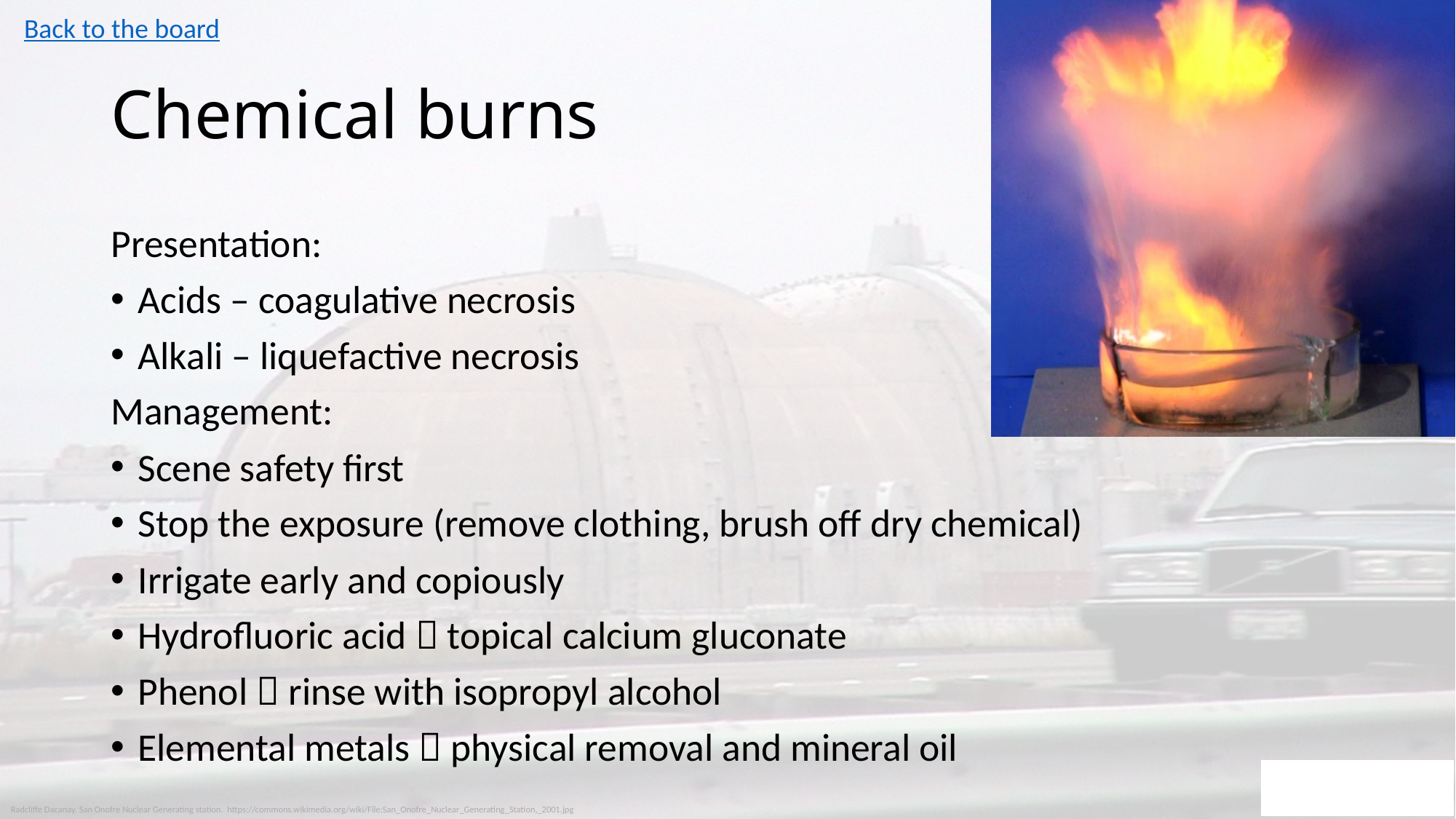

Back to the board
# Chemical burns
Presentation:
Acids – coagulative necrosis
Alkali – liquefactive necrosis
Management:
Scene safety first
Stop the exposure (remove clothing, brush off dry chemical)
Irrigate early and copiously
Hydrofluoric acid  topical calcium gluconate
Phenol  rinse with isopropyl alcohol
Elemental metals  physical removal and mineral oil
Radcliffe Dacanay. San Onofre Nuclear Generating station. https://commons.wikimedia.org/wiki/File:San_Onofre_Nuclear_Generating_Station,_2001.jpg

## Slide 16
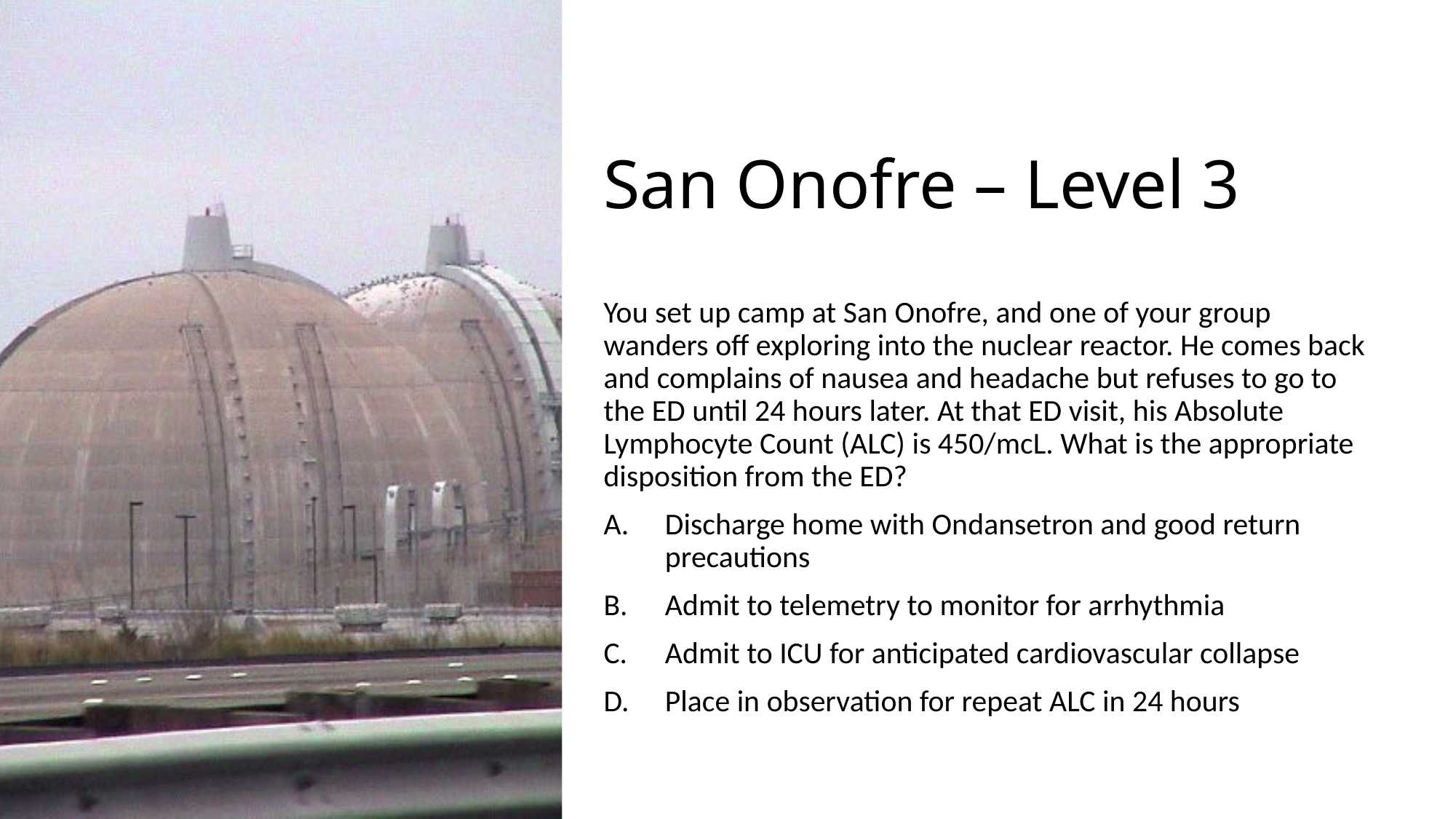

# San Onofre – Level 3
You set up camp at San Onofre, and one of your group wanders off exploring into the nuclear reactor. He comes back and complains of nausea and headache but refuses to go to the ED until 24 hours later. At that ED visit, his Absolute Lymphocyte Count (ALC) is 450/mcL. What is the appropriate disposition from the ED?
Discharge home with Ondansetron and good return precautions
Admit to telemetry to monitor for arrhythmia
Admit to ICU for anticipated cardiovascular collapse
Place in observation for repeat ALC in 24 hours

## Slide 17
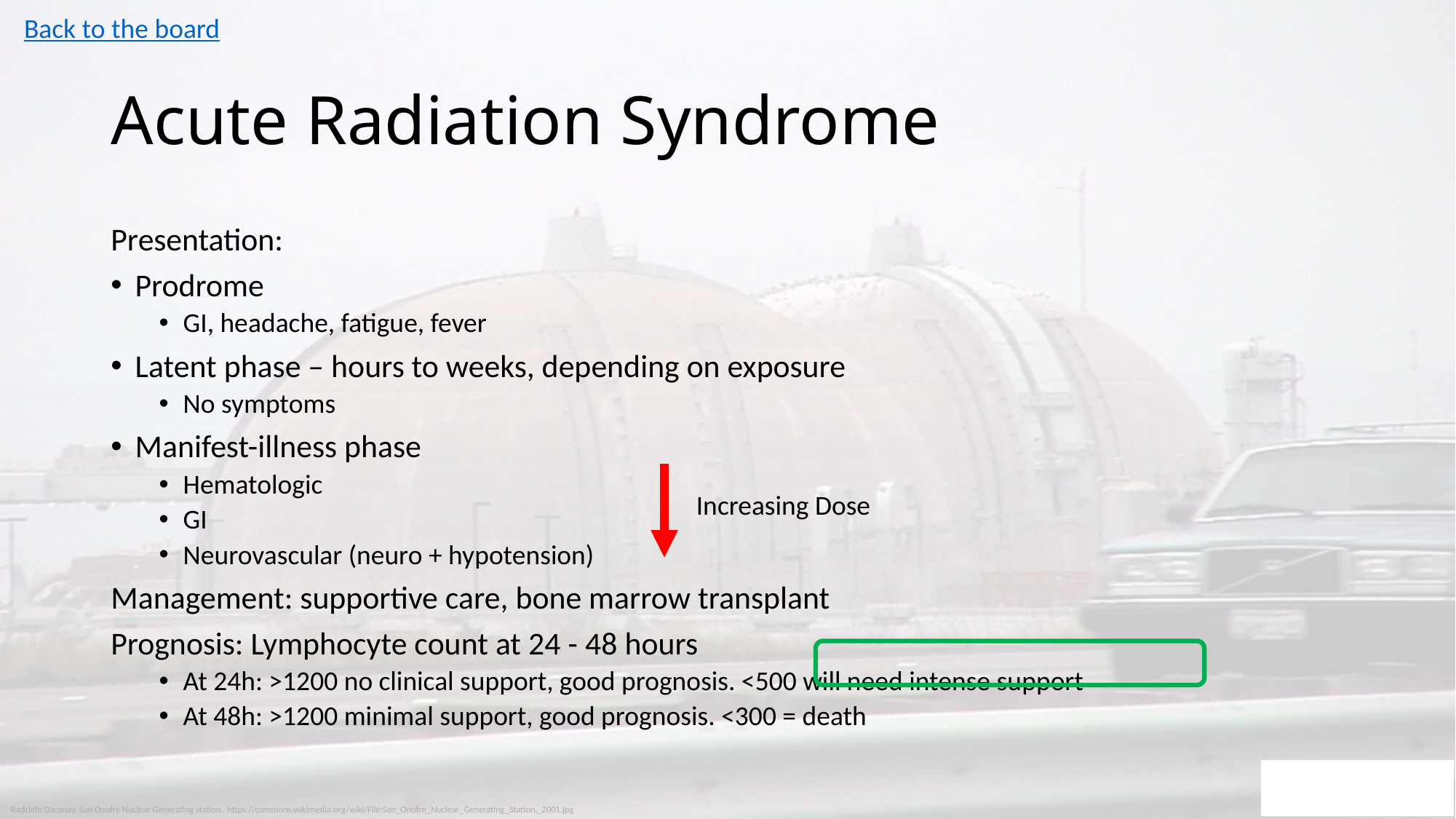

Back to the board
# Acute Radiation Syndrome
Presentation:
Prodrome
GI, headache, fatigue, fever
Latent phase – hours to weeks, depending on exposure
No symptoms
Manifest-illness phase
Hematologic
GI
Neurovascular (neuro + hypotension)
Management: supportive care, bone marrow transplant
Prognosis: Lymphocyte count at 24 - 48 hours
At 24h: >1200 no clinical support, good prognosis. <500 will need intense support
At 48h: >1200 minimal support, good prognosis. <300 = death
Increasing Dose
Radcliffe Dacanay. San Onofre Nuclear Generating station. https://commons.wikimedia.org/wiki/File:San_Onofre_Nuclear_Generating_Station,_2001.jpg

## Slide 18
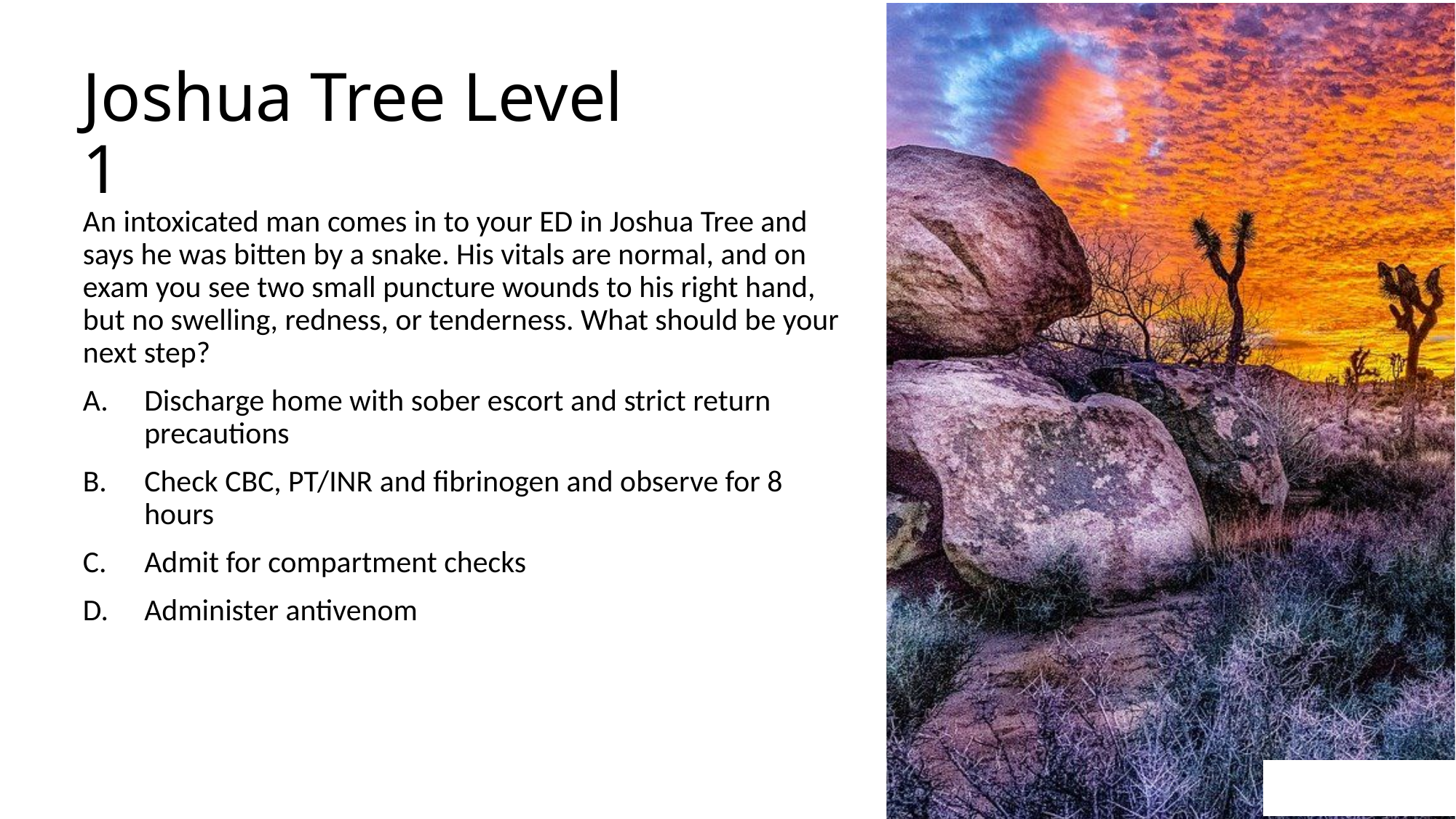

# Joshua Tree Level 1
An intoxicated man comes in to your ED in Joshua Tree and says he was bitten by a snake. His vitals are normal, and on exam you see two small puncture wounds to his right hand, but no swelling, redness, or tenderness. What should be your next step?
Discharge home with sober escort and strict return precautions
Check CBC, PT/INR and fibrinogen and observe for 8 hours
Admit for compartment checks
Administer antivenom

## Slide 19
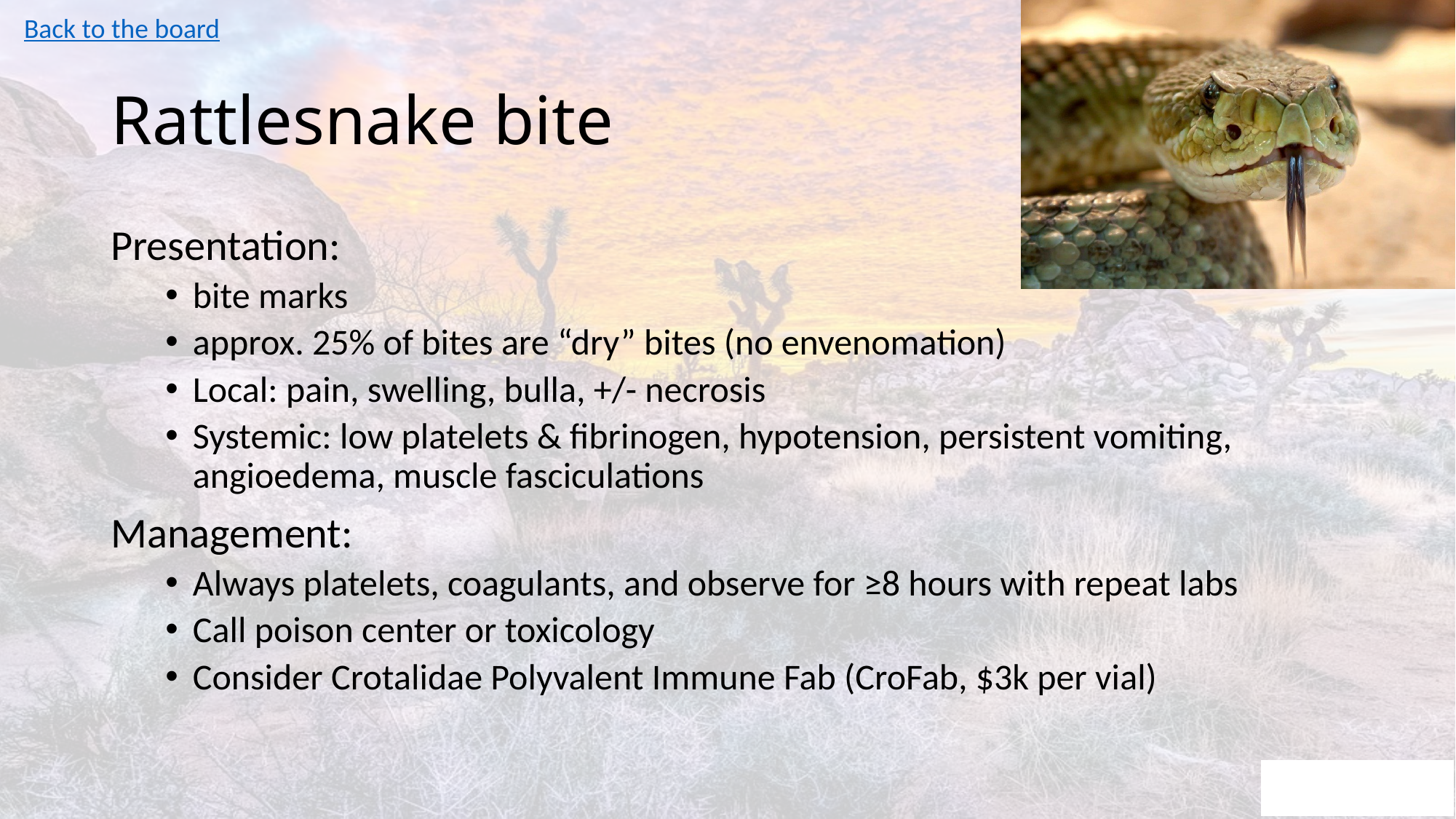

Back to the board
# Rattlesnake bite
Presentation:
bite marks
approx. 25% of bites are “dry” bites (no envenomation)
Local: pain, swelling, bulla, +/- necrosis
Systemic: low platelets & fibrinogen, hypotension, persistent vomiting, angioedema, muscle fasciculations
Management:
Always platelets, coagulants, and observe for ≥8 hours with repeat labs
Call poison center or toxicology
Consider Crotalidae Polyvalent Immune Fab (CroFab, $3k per vial)

## Slide 20
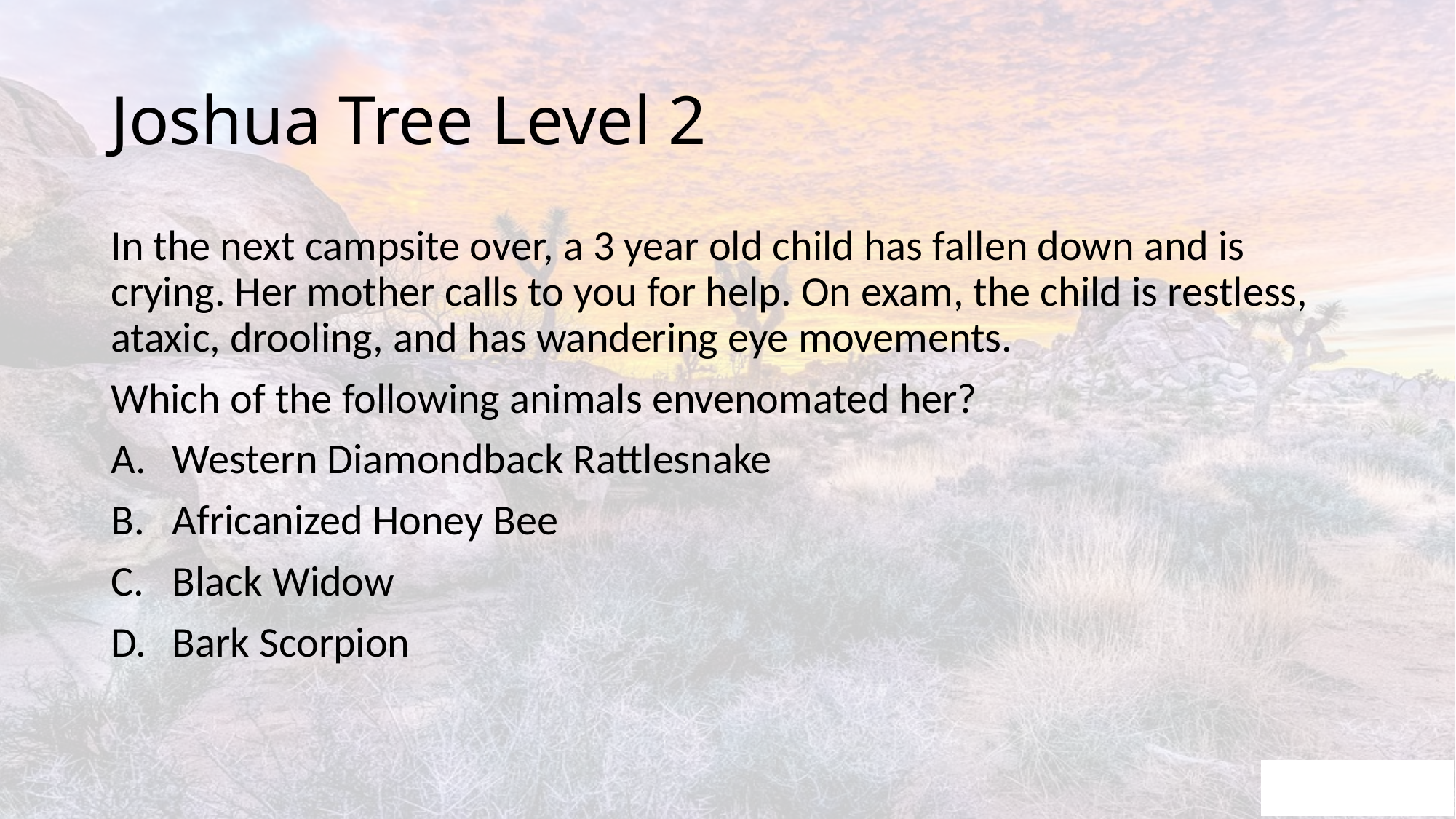

# Joshua Tree Level 2
In the next campsite over, a 3 year old child has fallen down and is crying. Her mother calls to you for help. On exam, the child is restless, ataxic, drooling, and has wandering eye movements.
Which of the following animals envenomated her?
Western Diamondback Rattlesnake
Africanized Honey Bee
Black Widow
Bark Scorpion

## Slide 21
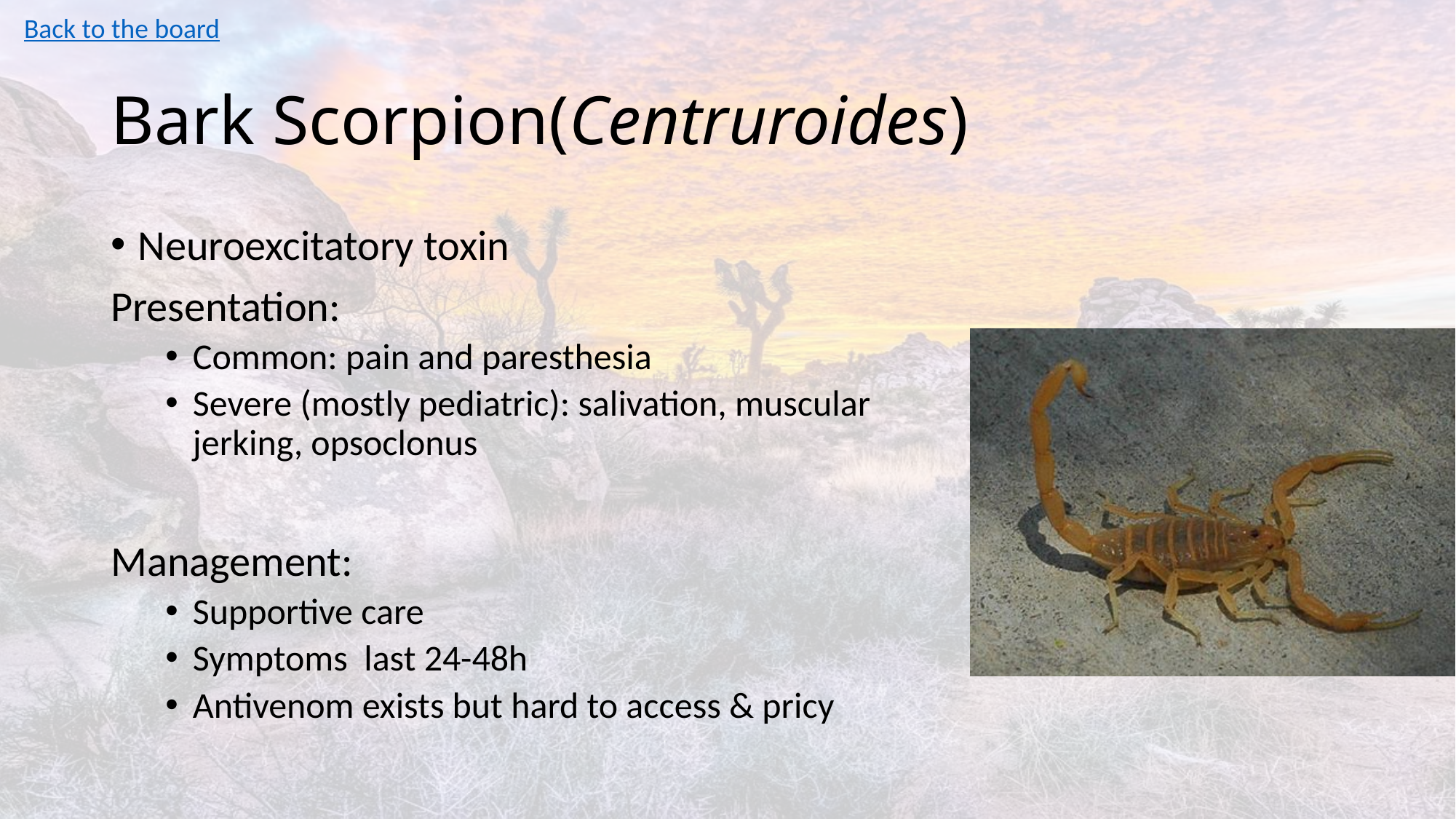

Back to the board
# Bark Scorpion(Centruroides)
Neuroexcitatory toxin
Presentation:
Common: pain and paresthesia
Severe (mostly pediatric): salivation, muscular jerking, opsoclonus
Management:
Supportive care
Symptoms last 24-48h
Antivenom exists but hard to access & pricy

## Slide 22
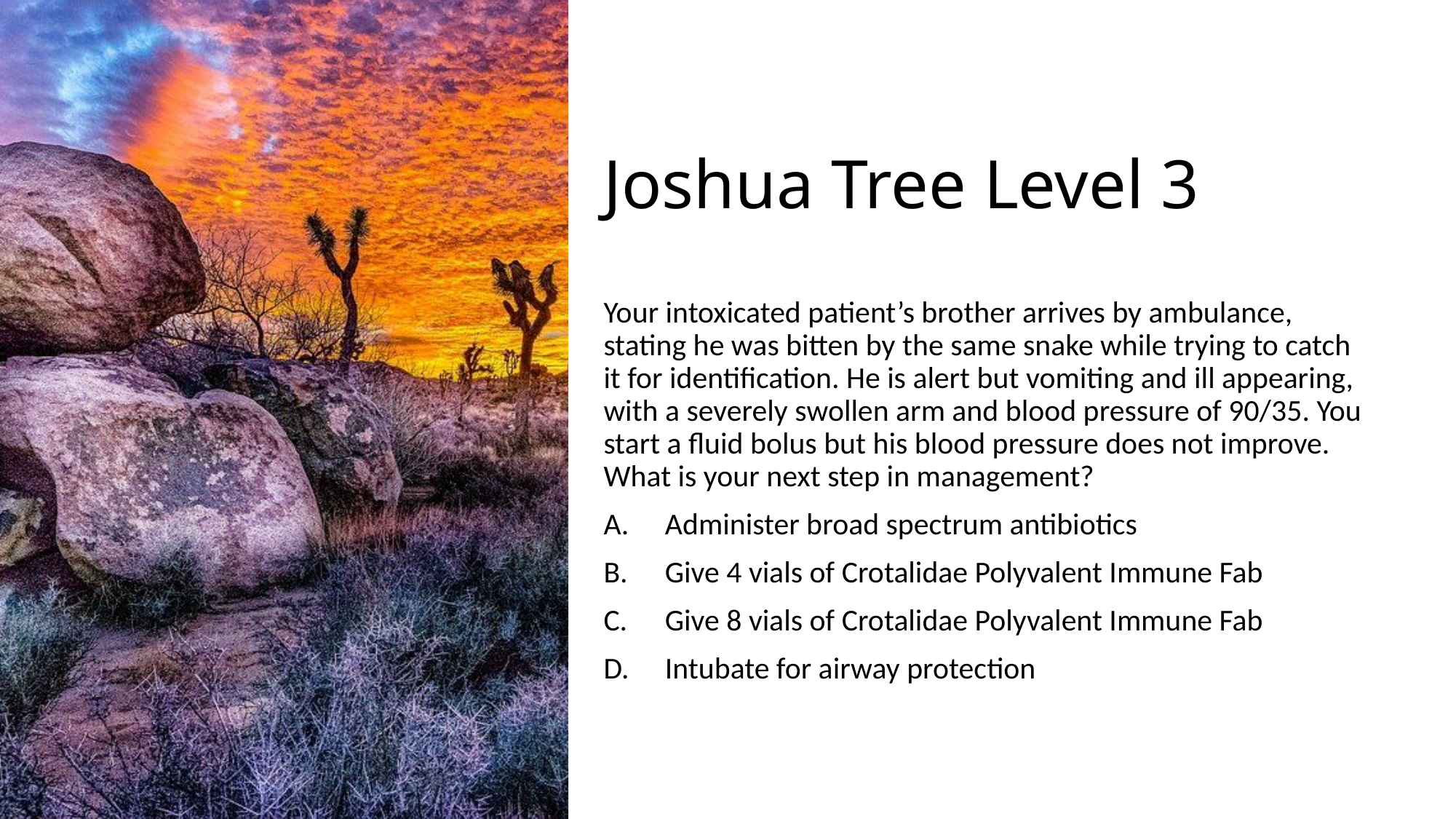

# Joshua Tree Level 3
Your intoxicated patient’s brother arrives by ambulance, stating he was bitten by the same snake while trying to catch it for identification. He is alert but vomiting and ill appearing, with a severely swollen arm and blood pressure of 90/35. You start a fluid bolus but his blood pressure does not improve. What is your next step in management?
Administer broad spectrum antibiotics
Give 4 vials of Crotalidae Polyvalent Immune Fab
Give 8 vials of Crotalidae Polyvalent Immune Fab
Intubate for airway protection

## Slide 23
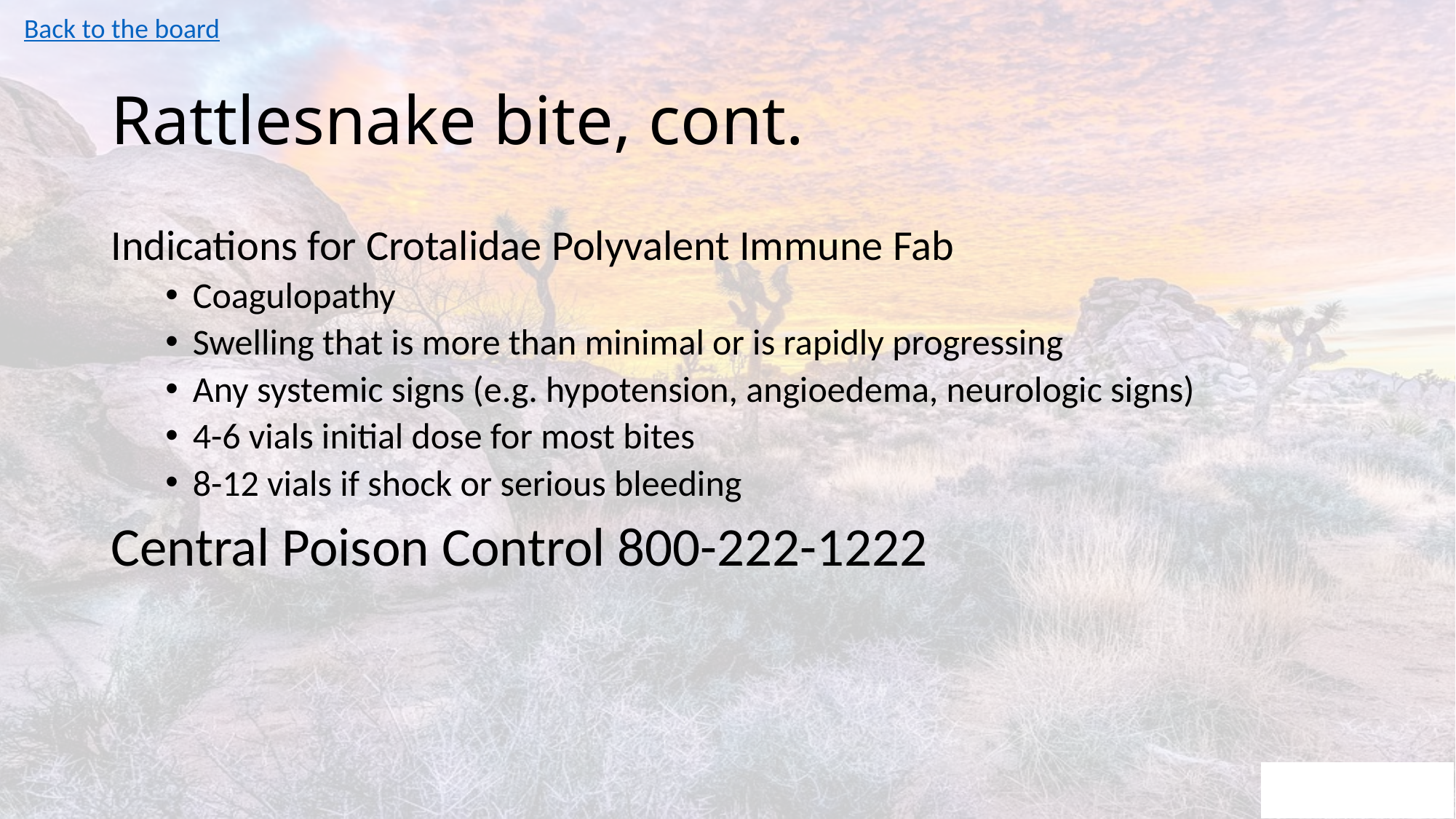

Back to the board
# Rattlesnake bite, cont.
Indications for Crotalidae Polyvalent Immune Fab
Coagulopathy
Swelling that is more than minimal or is rapidly progressing
Any systemic signs (e.g. hypotension, angioedema, neurologic signs)
4-6 vials initial dose for most bites
8-12 vials if shock or serious bleeding
Central Poison Control 800-222-1222

## Slide 24
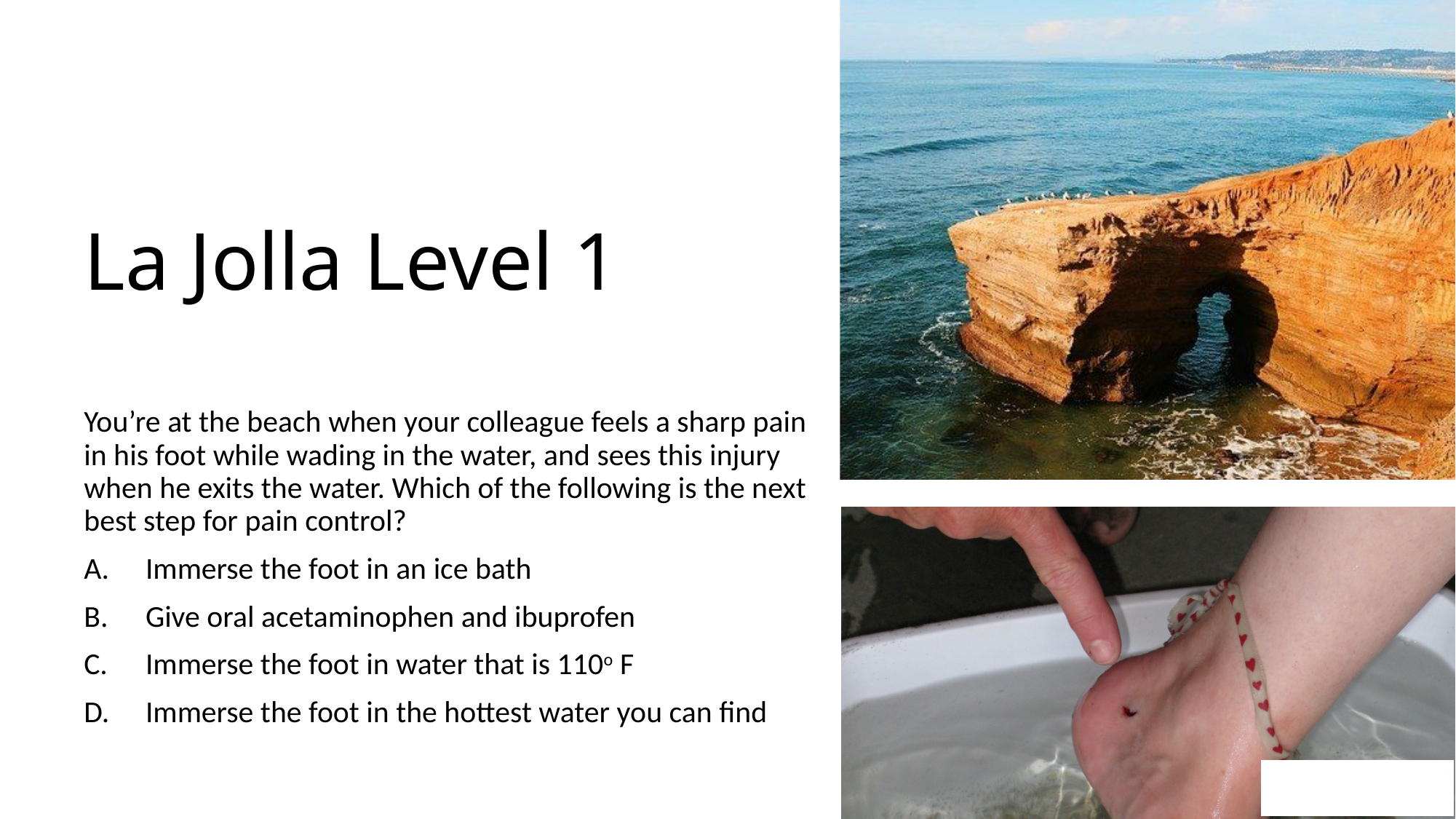

# La Jolla Level 1
You’re at the beach when your colleague feels a sharp pain in his foot while wading in the water, and sees this injury when he exits the water. Which of the following is the next best step for pain control?
Immerse the foot in an ice bath
Give oral acetaminophen and ibuprofen
Immerse the foot in water that is 110o F
Immerse the foot in the hottest water you can find

## Slide 25
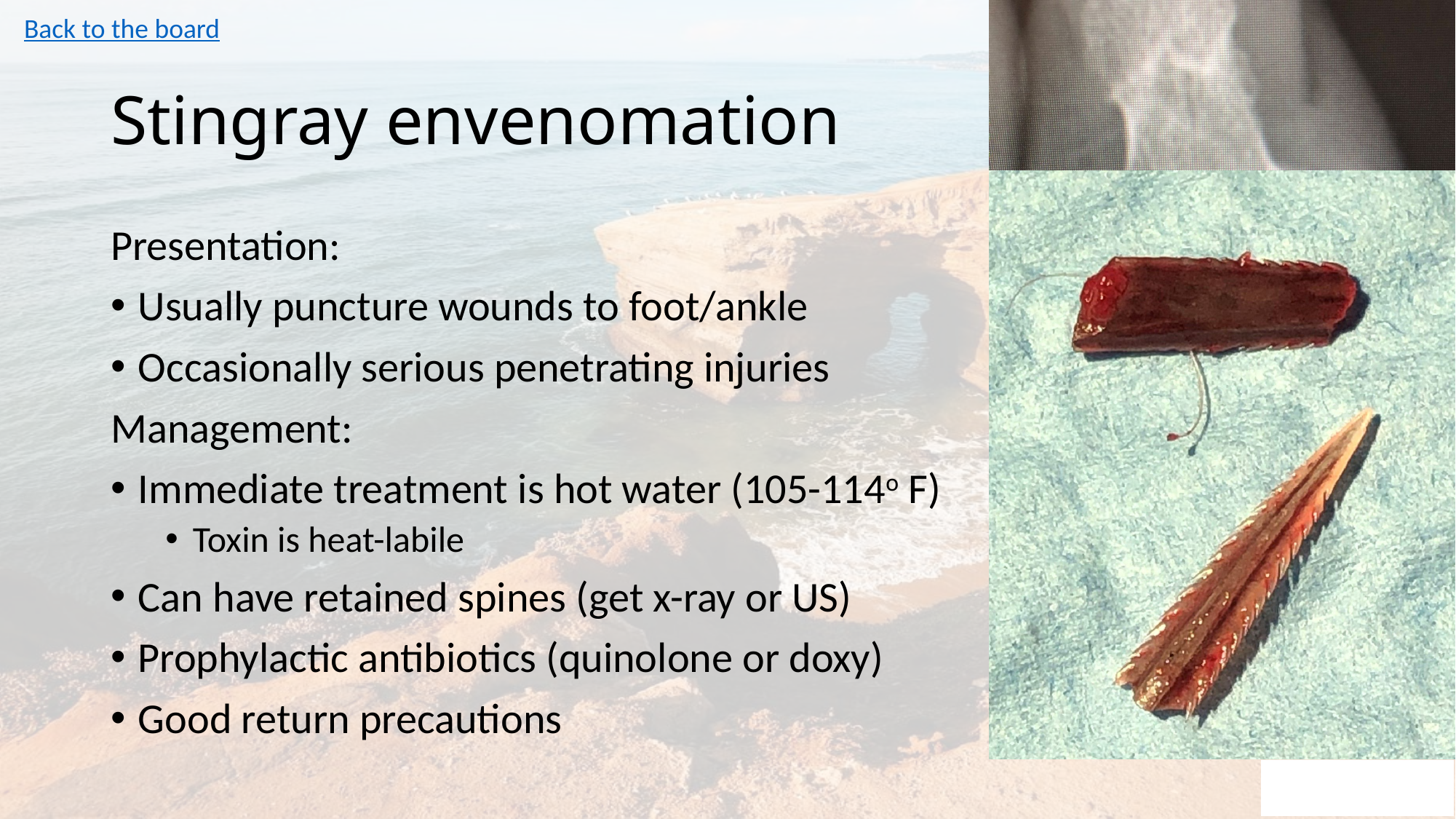

Back to the board
# Stingray envenomation
Presentation:
Usually puncture wounds to foot/ankle
Occasionally serious penetrating injuries
Management:
Immediate treatment is hot water (105-114o F)
Toxin is heat-labile
Can have retained spines (get x-ray or US)
Prophylactic antibiotics (quinolone or doxy)
Good return precautions

## Slide 26
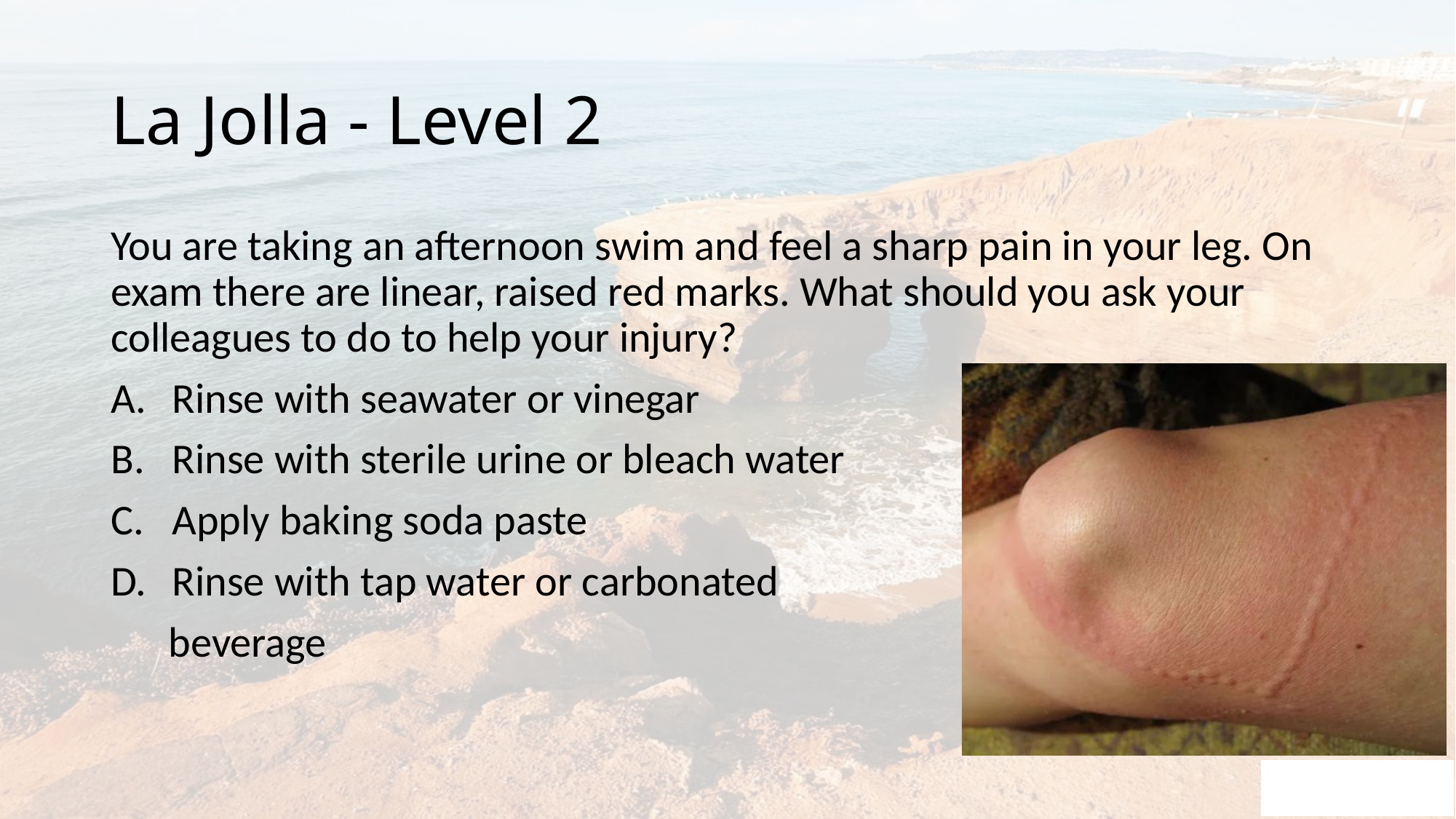

# La Jolla - Level 2
You are taking an afternoon swim and feel a sharp pain in your leg. On exam there are linear, raised red marks. What should you ask your colleagues to do to help your injury?
Rinse with seawater or vinegar
Rinse with sterile urine or bleach water
Apply baking soda paste
Rinse with tap water or carbonated
 beverage

## Slide 27
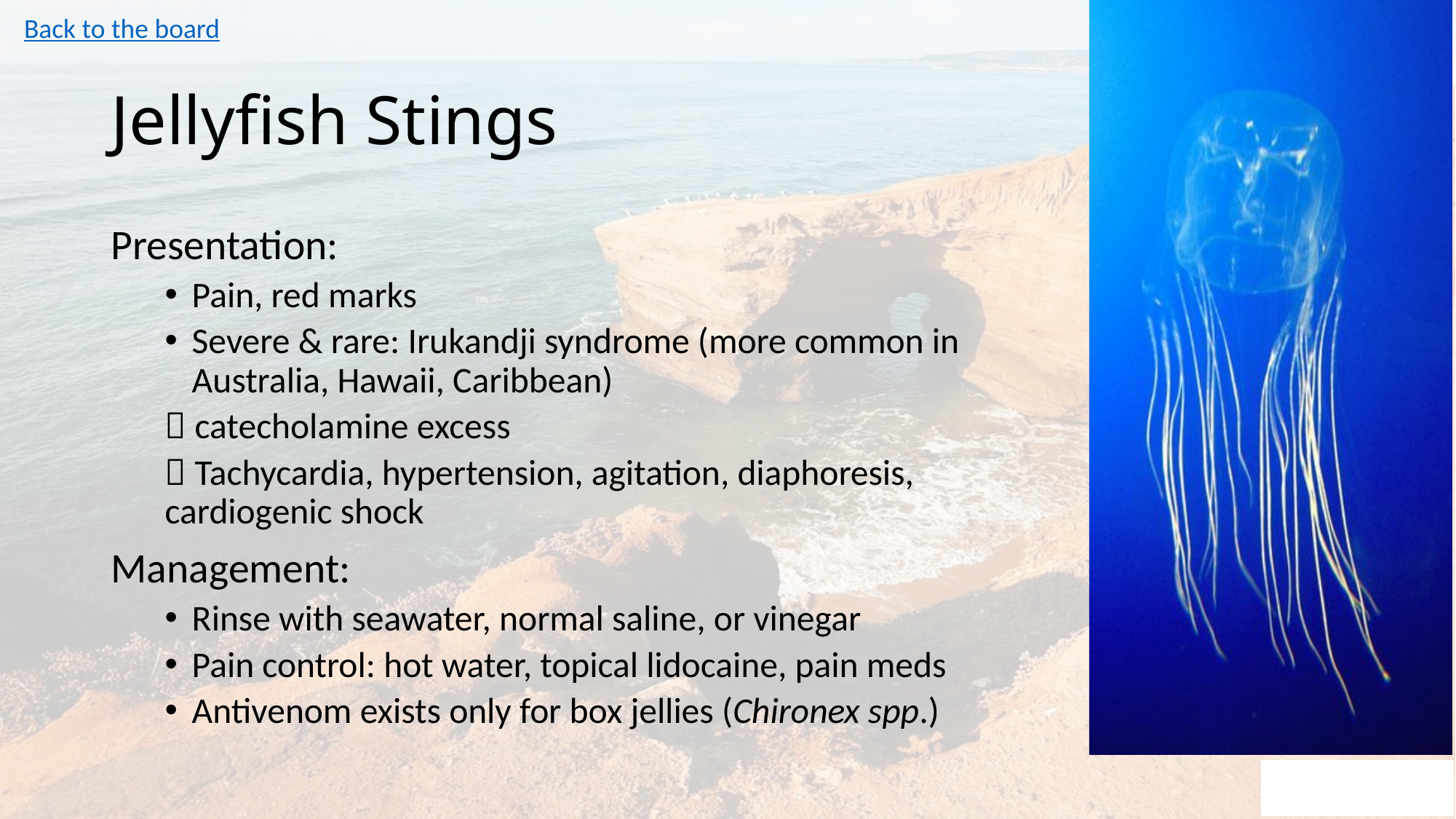

Back to the board
# Jellyfish Stings
Presentation:
Pain, red marks
Severe & rare: Irukandji syndrome (more common in Australia, Hawaii, Caribbean)
	 catecholamine excess
	 Tachycardia, hypertension, agitation, diaphoresis, cardiogenic shock
Management:
Rinse with seawater, normal saline, or vinegar
Pain control: hot water, topical lidocaine, pain meds
Antivenom exists only for box jellies (Chironex spp.)

## Slide 28
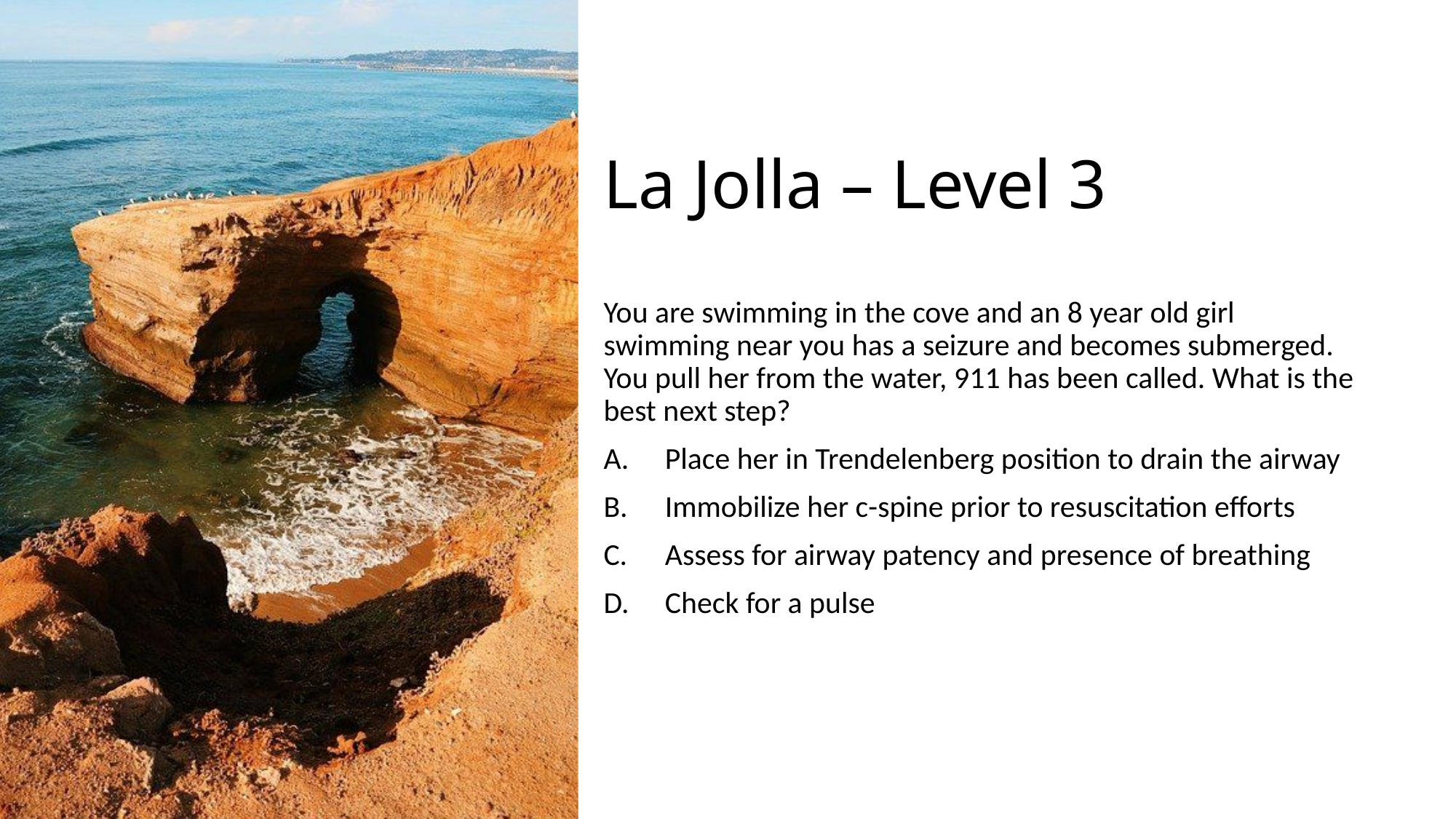

# La Jolla – Level 3
You are swimming in the cove and an 8 year old girl swimming near you has a seizure and becomes submerged. You pull her from the water, 911 has been called. What is the best next step?
Place her in Trendelenberg position to drain the airway
Immobilize her c-spine prior to resuscitation efforts
Assess for airway patency and presence of breathing
Check for a pulse

## Slide 29
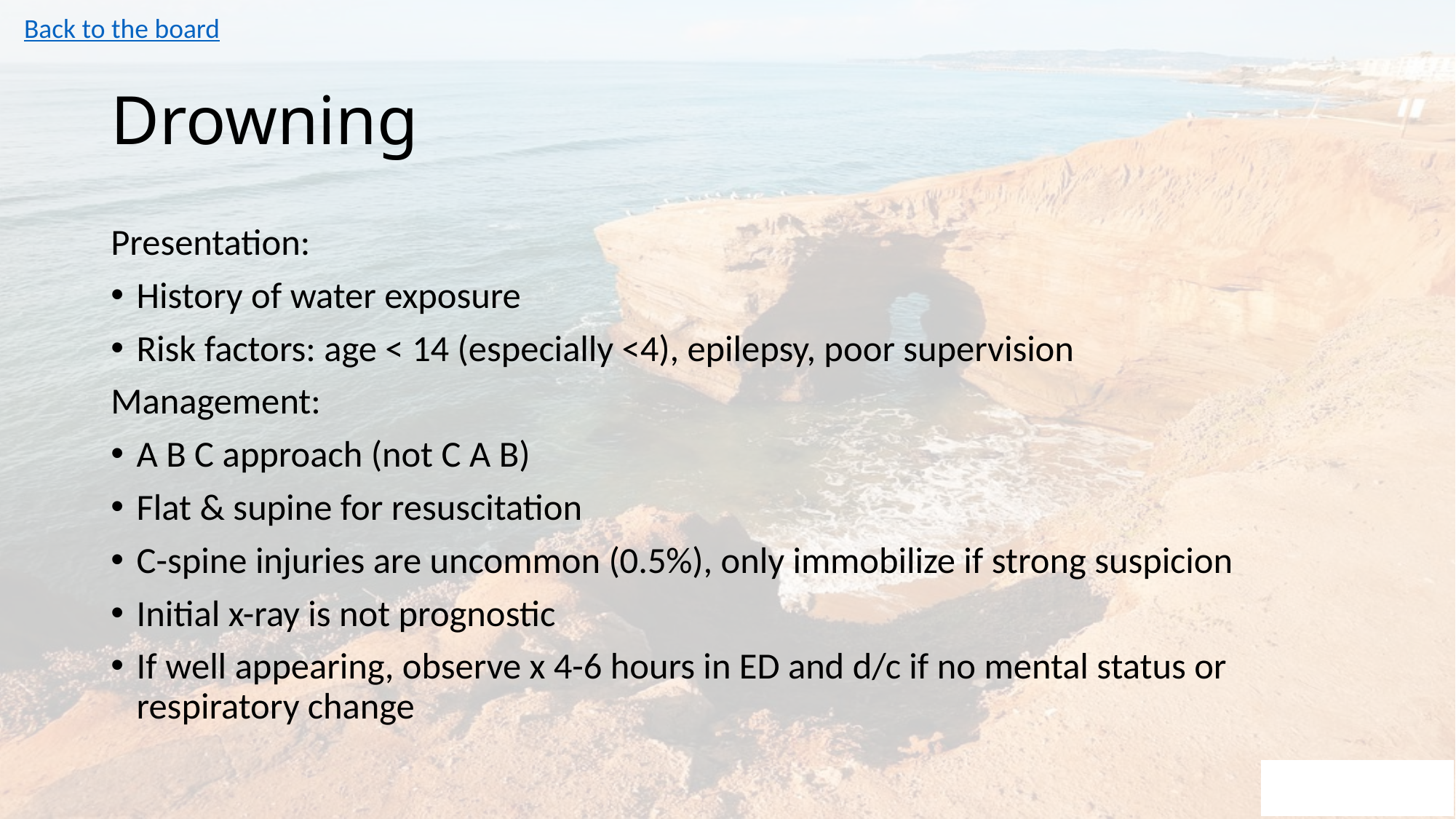

Back to the board
# Drowning
Presentation:
History of water exposure
Risk factors: age < 14 (especially <4), epilepsy, poor supervision
Management:
A B C approach (not C A B)
Flat & supine for resuscitation
C-spine injuries are uncommon (0.5%), only immobilize if strong suspicion
Initial x-ray is not prognostic
If well appearing, observe x 4-6 hours in ED and d/c if no mental status or respiratory change

## Slide 30
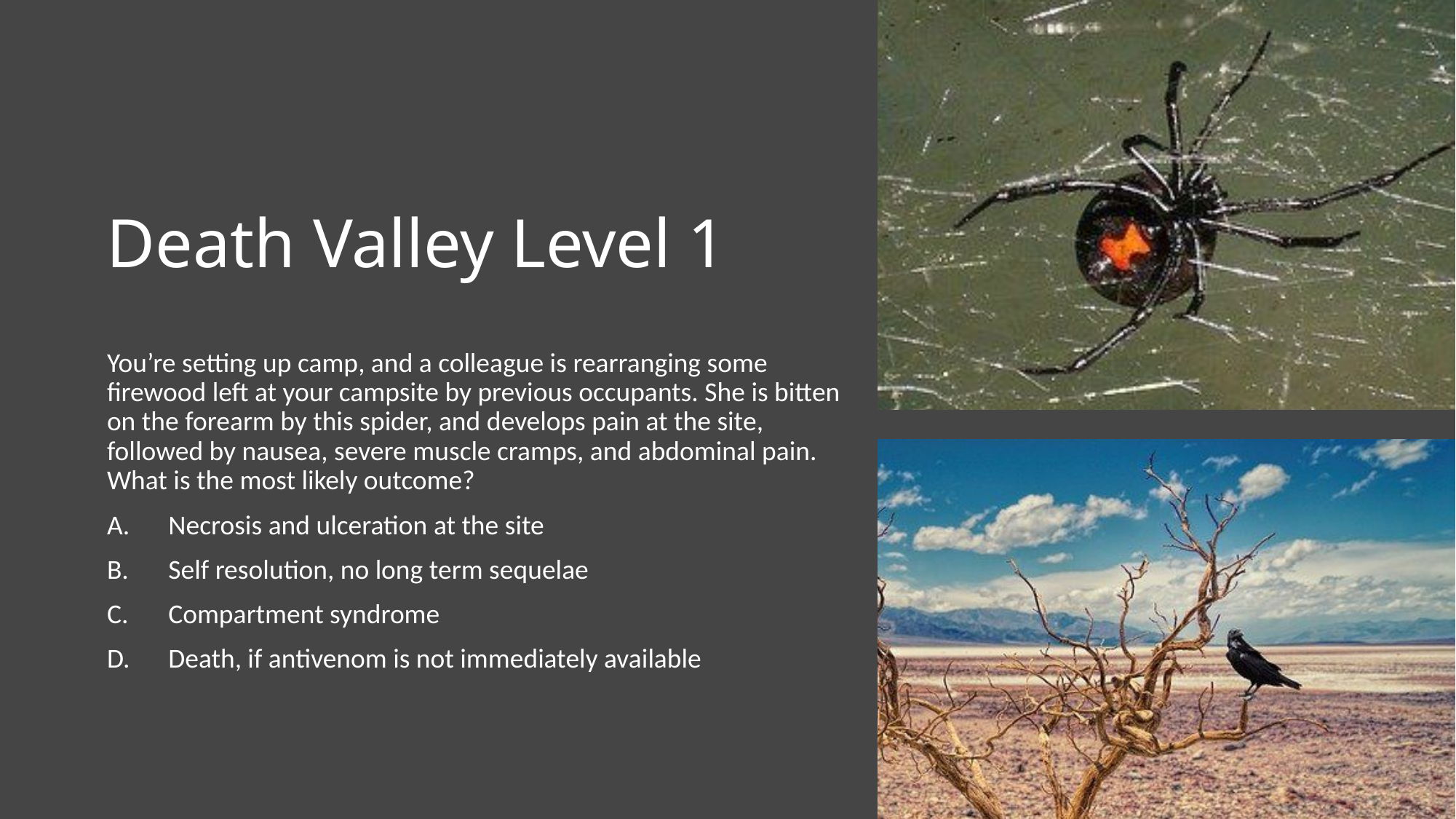

# Death Valley Level 1
You’re setting up camp, and a colleague is rearranging some firewood left at your campsite by previous occupants. She is bitten on the forearm by this spider, and develops pain at the site, followed by nausea, severe muscle cramps, and abdominal pain. What is the most likely outcome?
Necrosis and ulceration at the site
Self resolution, no long term sequelae
Compartment syndrome
Death, if antivenom is not immediately available

## Slide 31
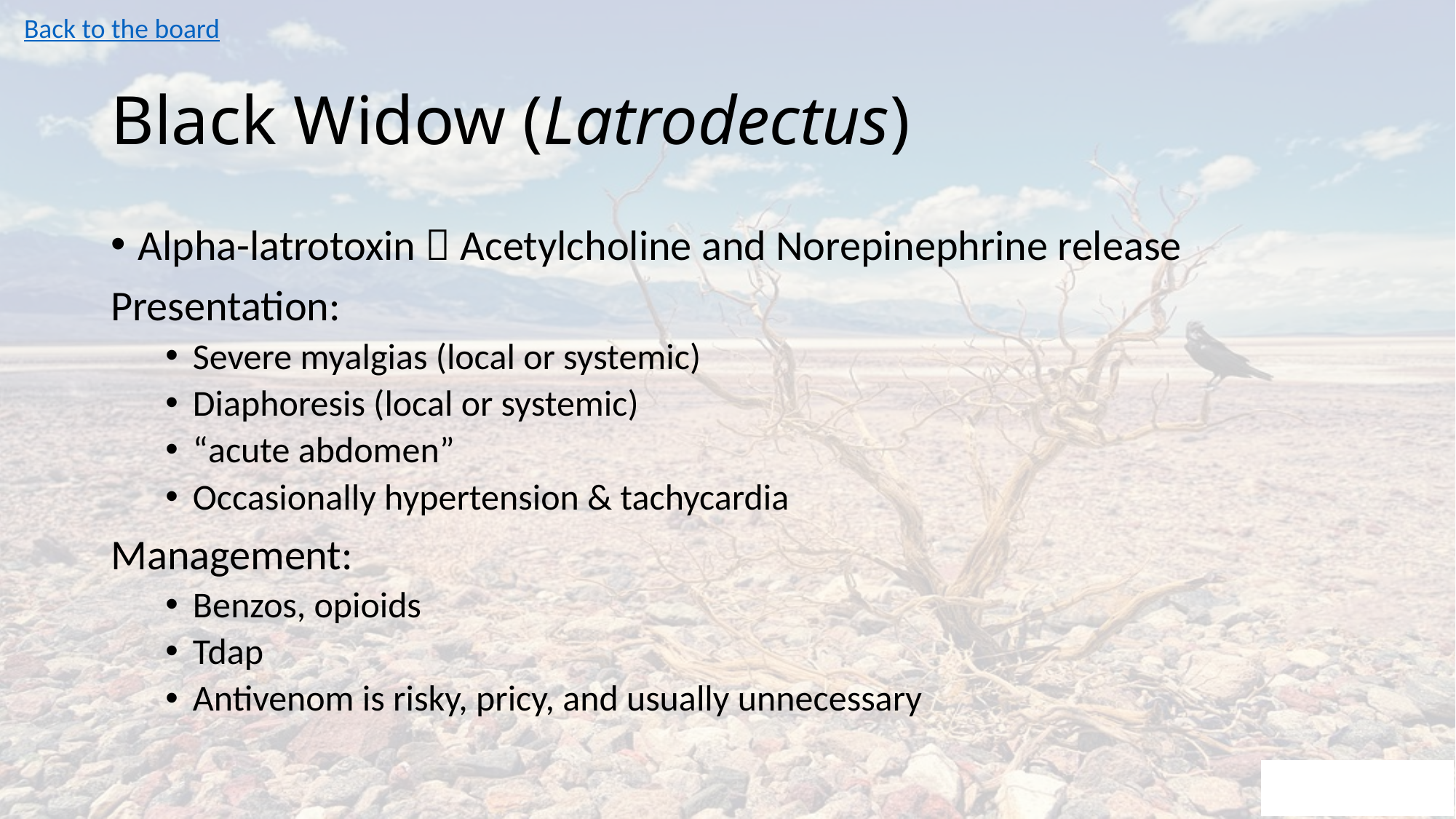

Back to the board
# Black Widow (Latrodectus)
Alpha-latrotoxin  Acetylcholine and Norepinephrine release
Presentation:
Severe myalgias (local or systemic)
Diaphoresis (local or systemic)
“acute abdomen”
Occasionally hypertension & tachycardia
Management:
Benzos, opioids
Tdap
Antivenom is risky, pricy, and usually unnecessary

## Slide 32
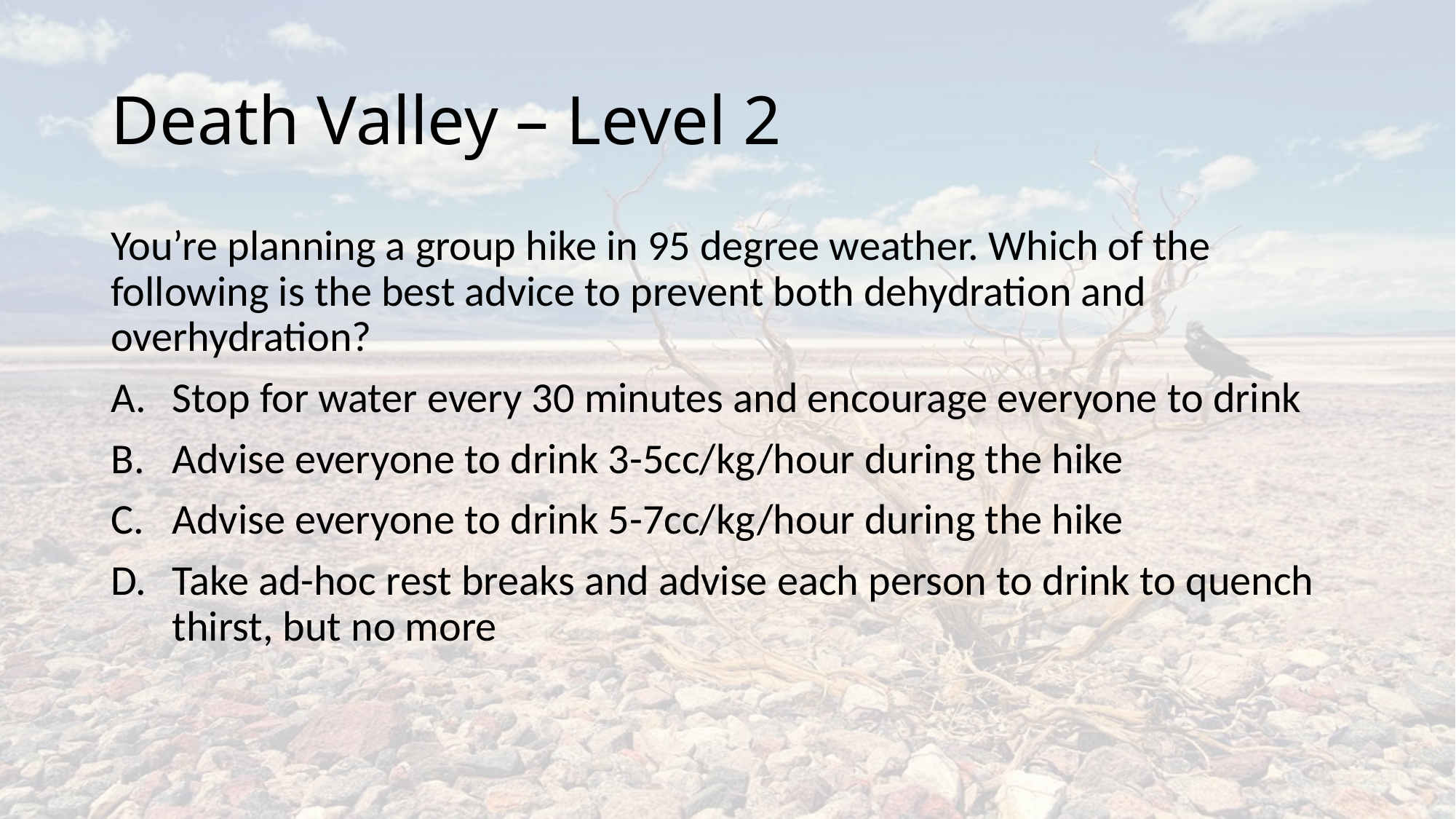

# Death Valley – Level 2
You’re planning a group hike in 95 degree weather. Which of the following is the best advice to prevent both dehydration and overhydration?
Stop for water every 30 minutes and encourage everyone to drink
Advise everyone to drink 3-5cc/kg/hour during the hike
Advise everyone to drink 5-7cc/kg/hour during the hike
Take ad-hoc rest breaks and advise each person to drink to quench thirst, but no more

## Slide 33
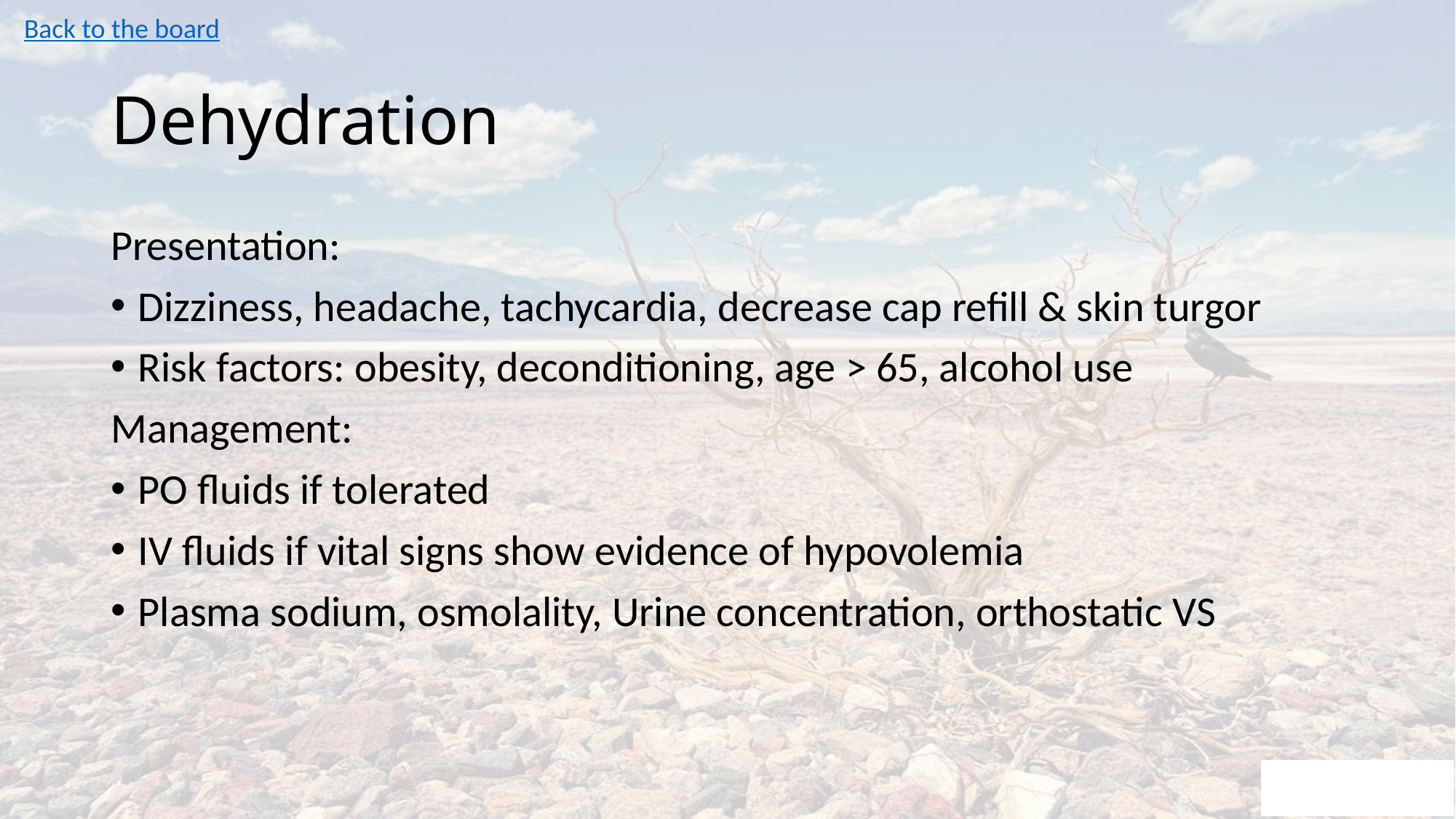

Back to the board
# Dehydration
Presentation:
Dizziness, headache, tachycardia, decrease cap refill & skin turgor
Risk factors: obesity, deconditioning, age > 65, alcohol use
Management:
PO fluids if tolerated
IV fluids if vital signs show evidence of hypovolemia
Plasma sodium, osmolality, Urine concentration, orthostatic VS

## Slide 34
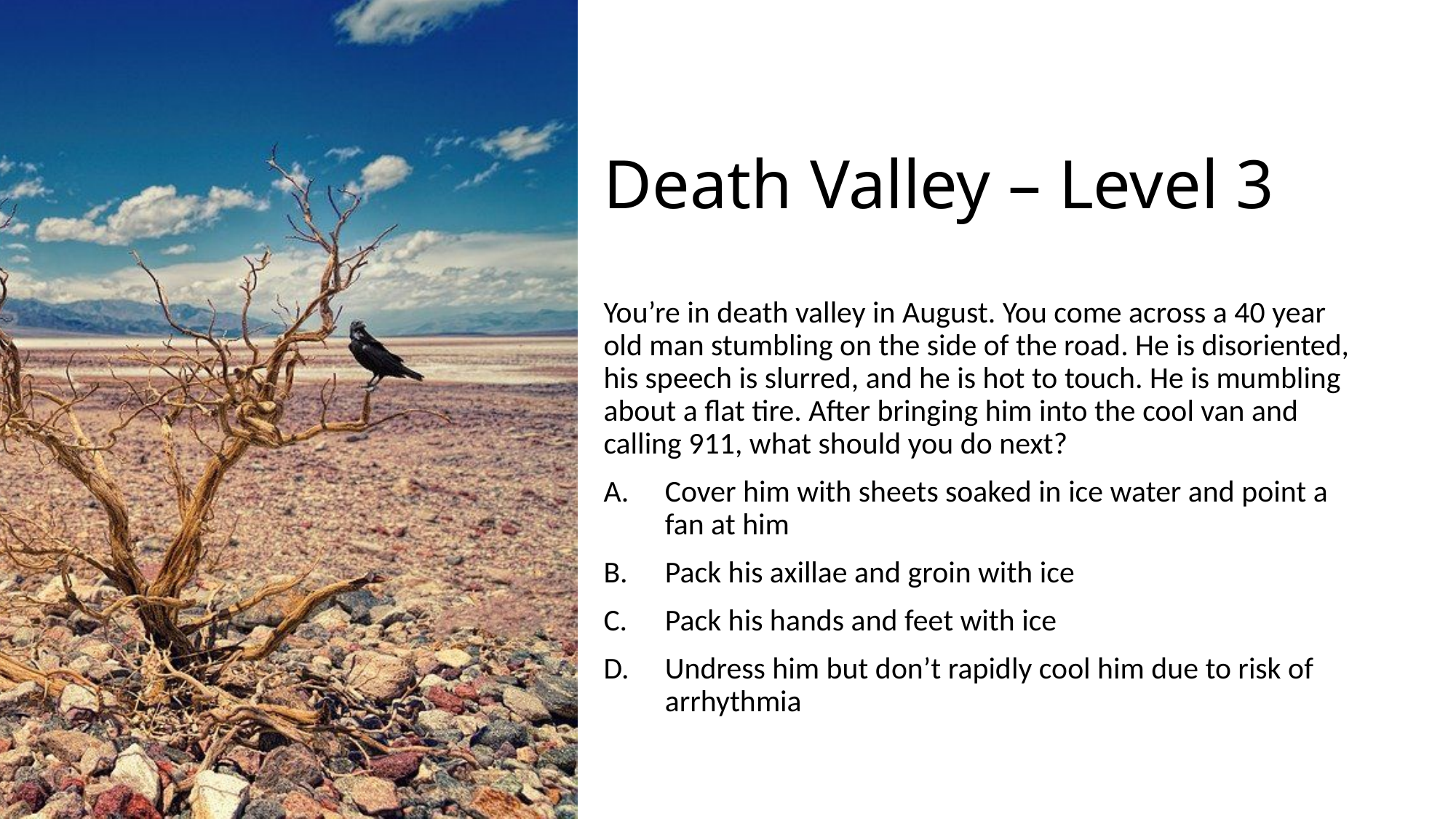

# Death Valley – Level 3
You’re in death valley in August. You come across a 40 year old man stumbling on the side of the road. He is disoriented, his speech is slurred, and he is hot to touch. He is mumbling about a flat tire. After bringing him into the cool van and calling 911, what should you do next?
Cover him with sheets soaked in ice water and point a fan at him
Pack his axillae and groin with ice
Pack his hands and feet with ice
Undress him but don’t rapidly cool him due to risk of arrhythmia

## Slide 35
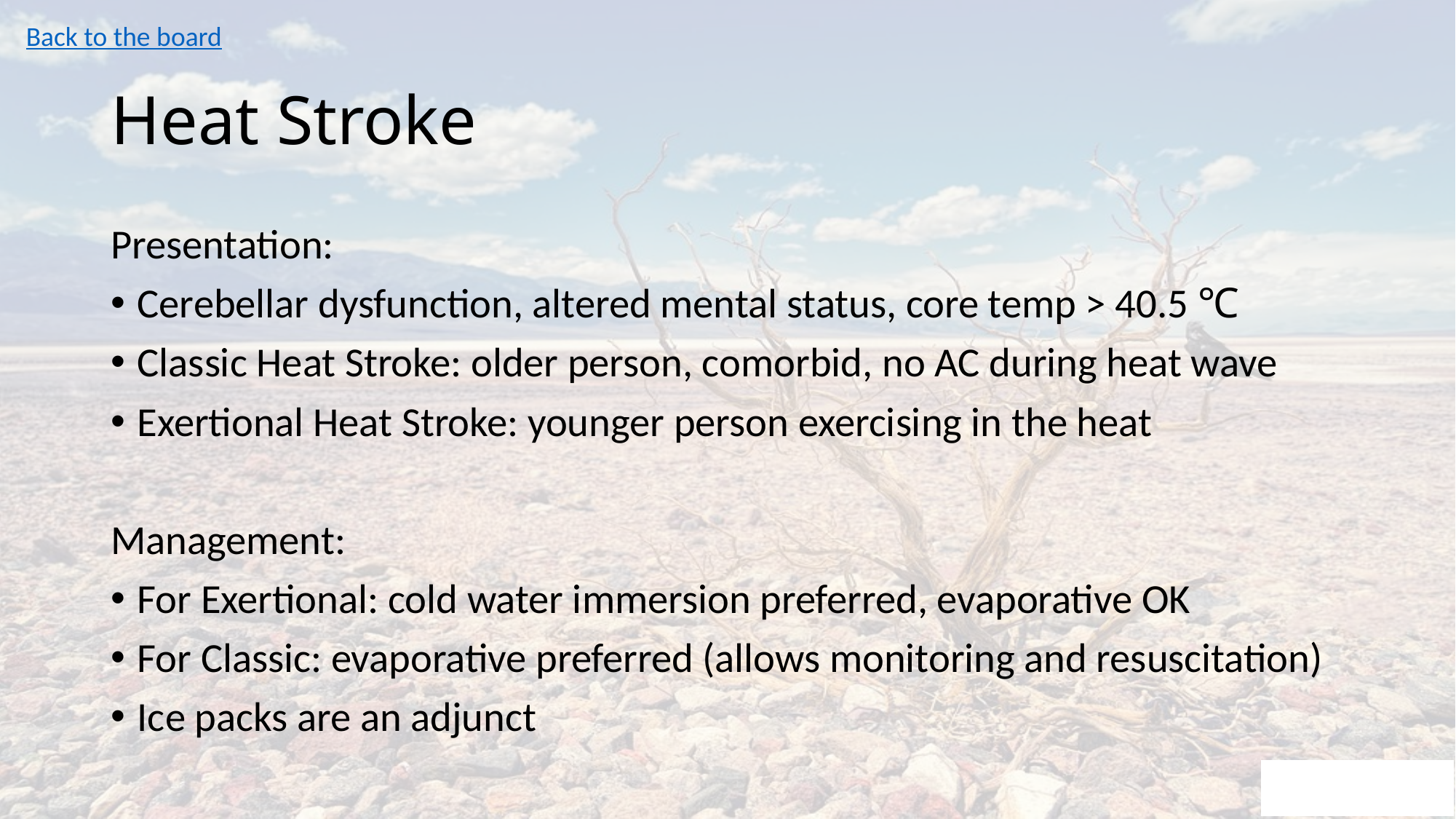

Back to the board
# Heat Stroke
Presentation:
Cerebellar dysfunction, altered mental status, core temp > 40.5 ℃
Classic Heat Stroke: older person, comorbid, no AC during heat wave
Exertional Heat Stroke: younger person exercising in the heat
Management:
For Exertional: cold water immersion preferred, evaporative OK
For Classic: evaporative preferred (allows monitoring and resuscitation)
Ice packs are an adjunct

## Slide 36
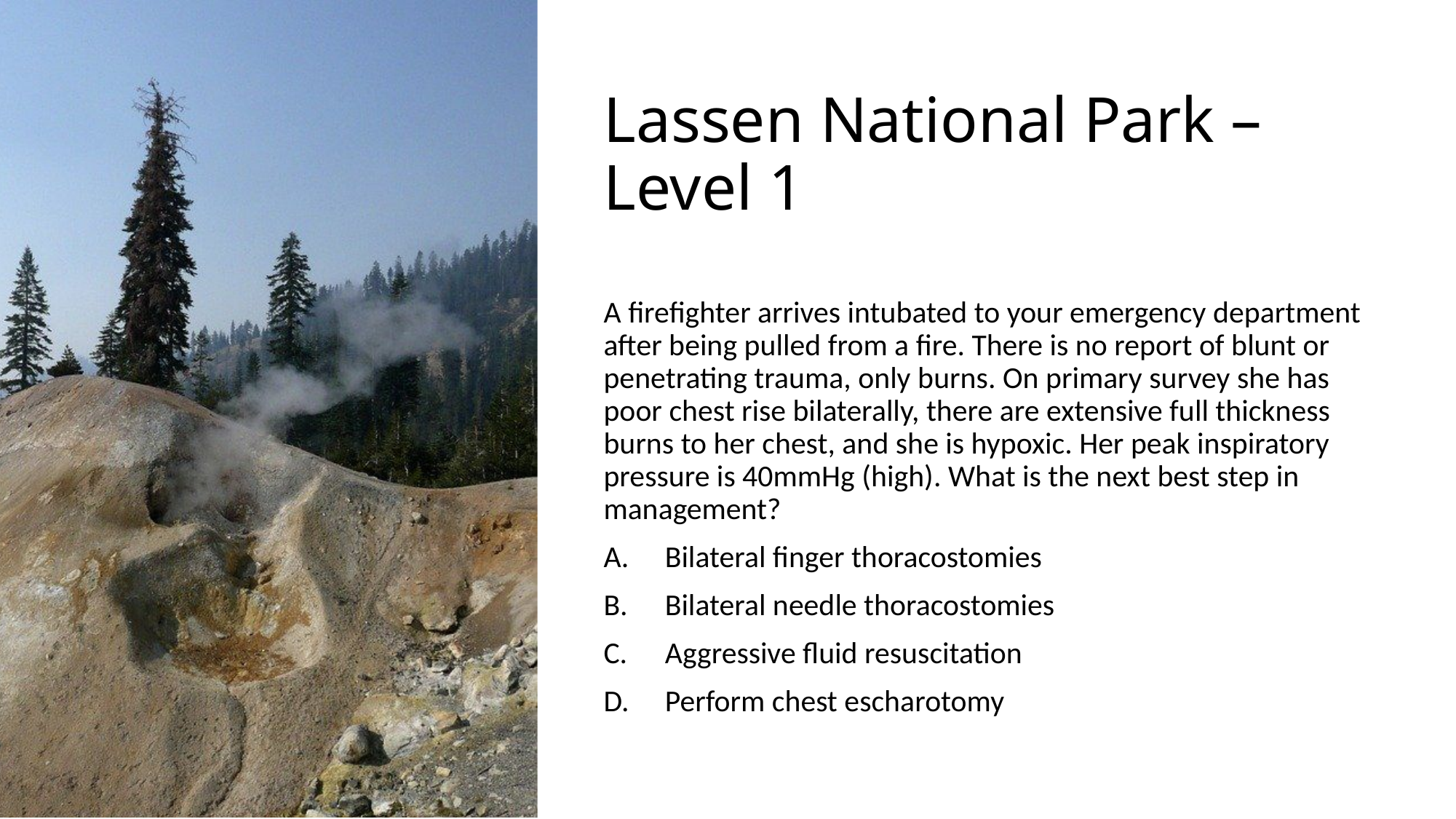

# Lassen National Park – Level 1
A firefighter arrives intubated to your emergency department after being pulled from a fire. There is no report of blunt or penetrating trauma, only burns. On primary survey she has poor chest rise bilaterally, there are extensive full thickness burns to her chest, and she is hypoxic. Her peak inspiratory pressure is 40mmHg (high). What is the next best step in management?
Bilateral finger thoracostomies
Bilateral needle thoracostomies
Aggressive fluid resuscitation
Perform chest escharotomy

## Slide 37
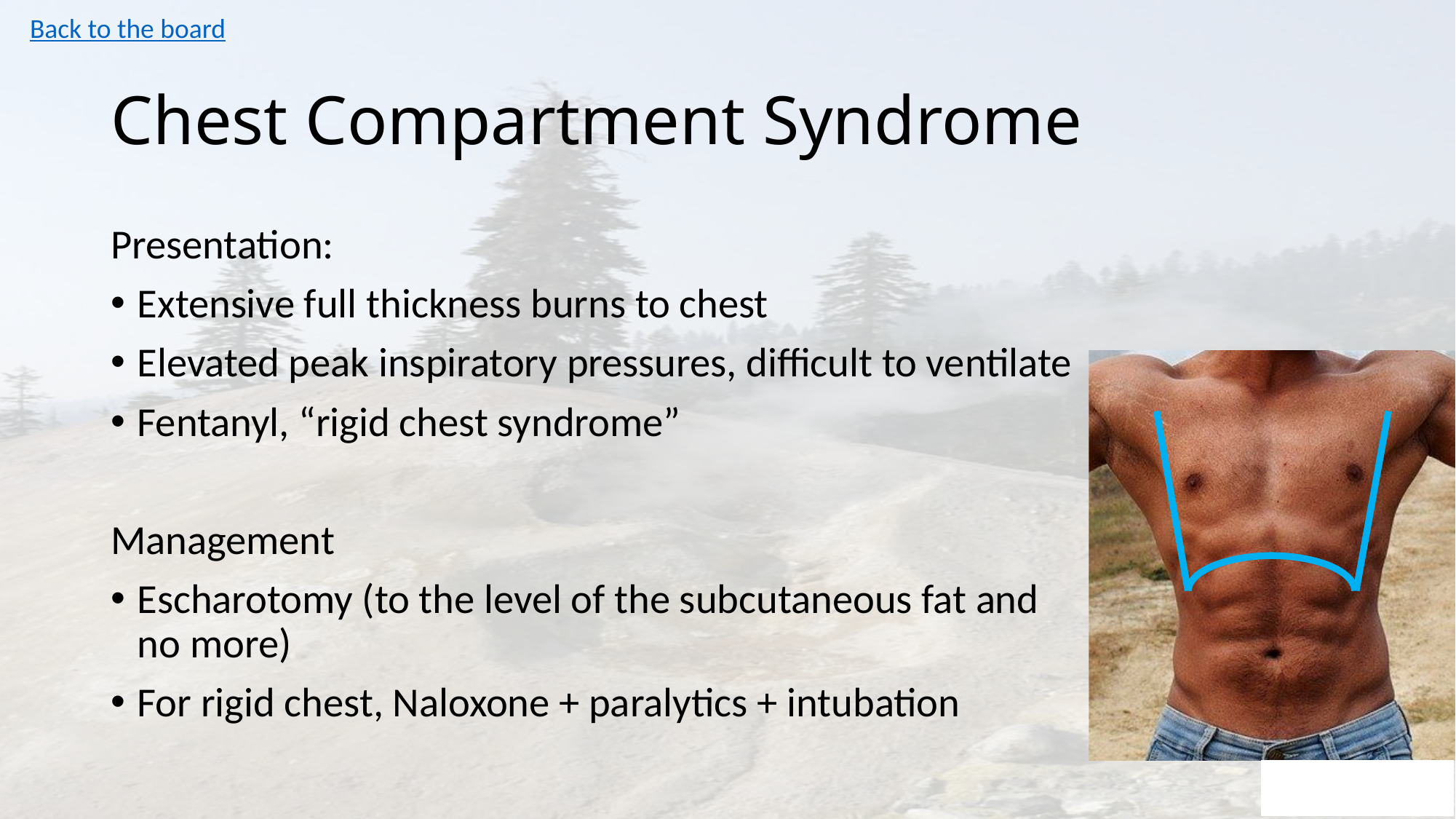

Back to the board
# Chest Compartment Syndrome
Presentation:
Extensive full thickness burns to chest
Elevated peak inspiratory pressures, difficult to ventilate
Fentanyl, “rigid chest syndrome”
Management
Escharotomy (to the level of the subcutaneous fat and no more)
For rigid chest, Naloxone + paralytics + intubation

## Slide 38
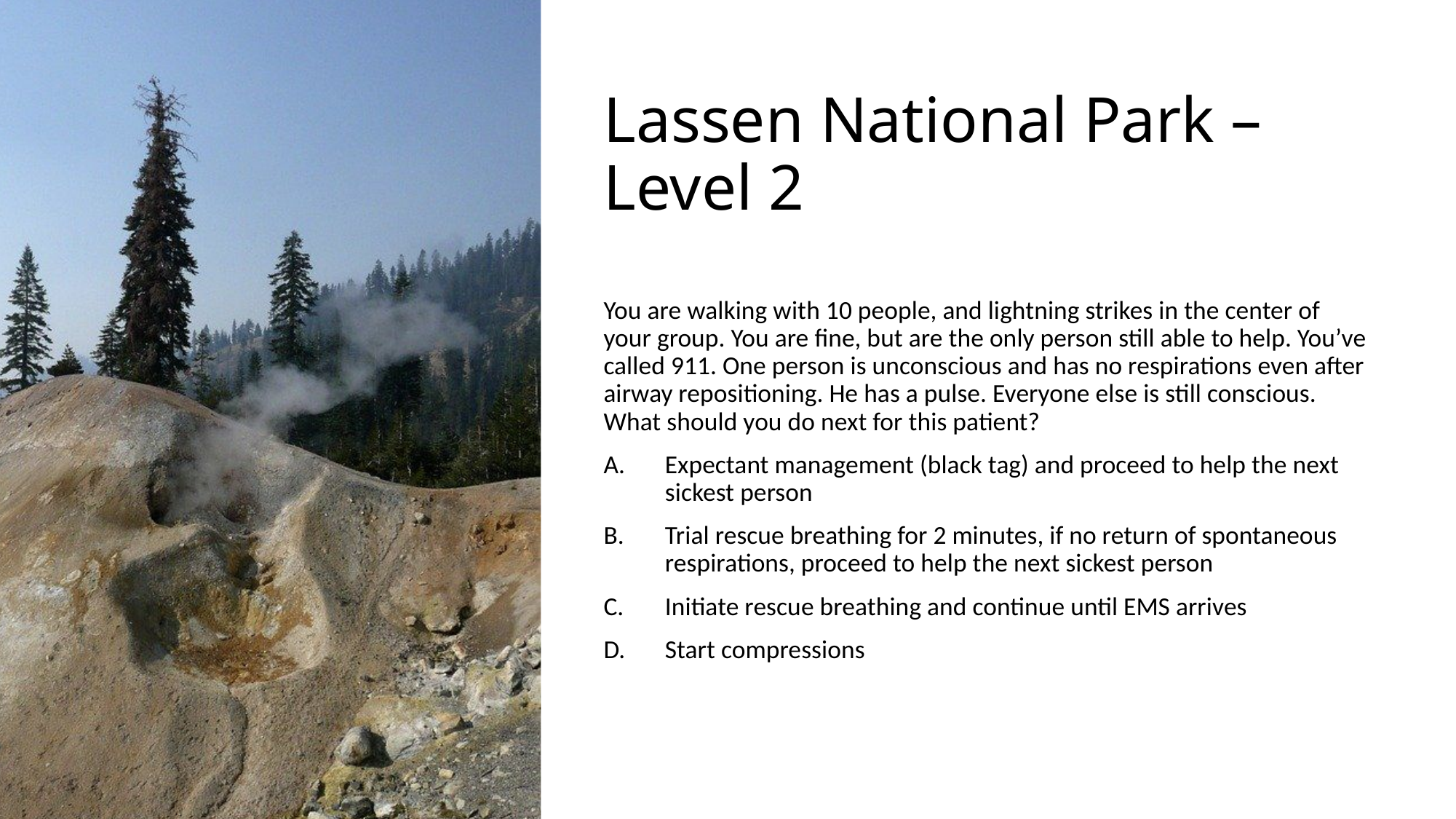

# Lassen National Park – Level 2
You are walking with 10 people, and lightning strikes in the center of your group. You are fine, but are the only person still able to help. You’ve called 911. One person is unconscious and has no respirations even after airway repositioning. He has a pulse. Everyone else is still conscious. What should you do next for this patient?
Expectant management (black tag) and proceed to help the next sickest person
Trial rescue breathing for 2 minutes, if no return of spontaneous respirations, proceed to help the next sickest person
Initiate rescue breathing and continue until EMS arrives
Start compressions

## Slide 39
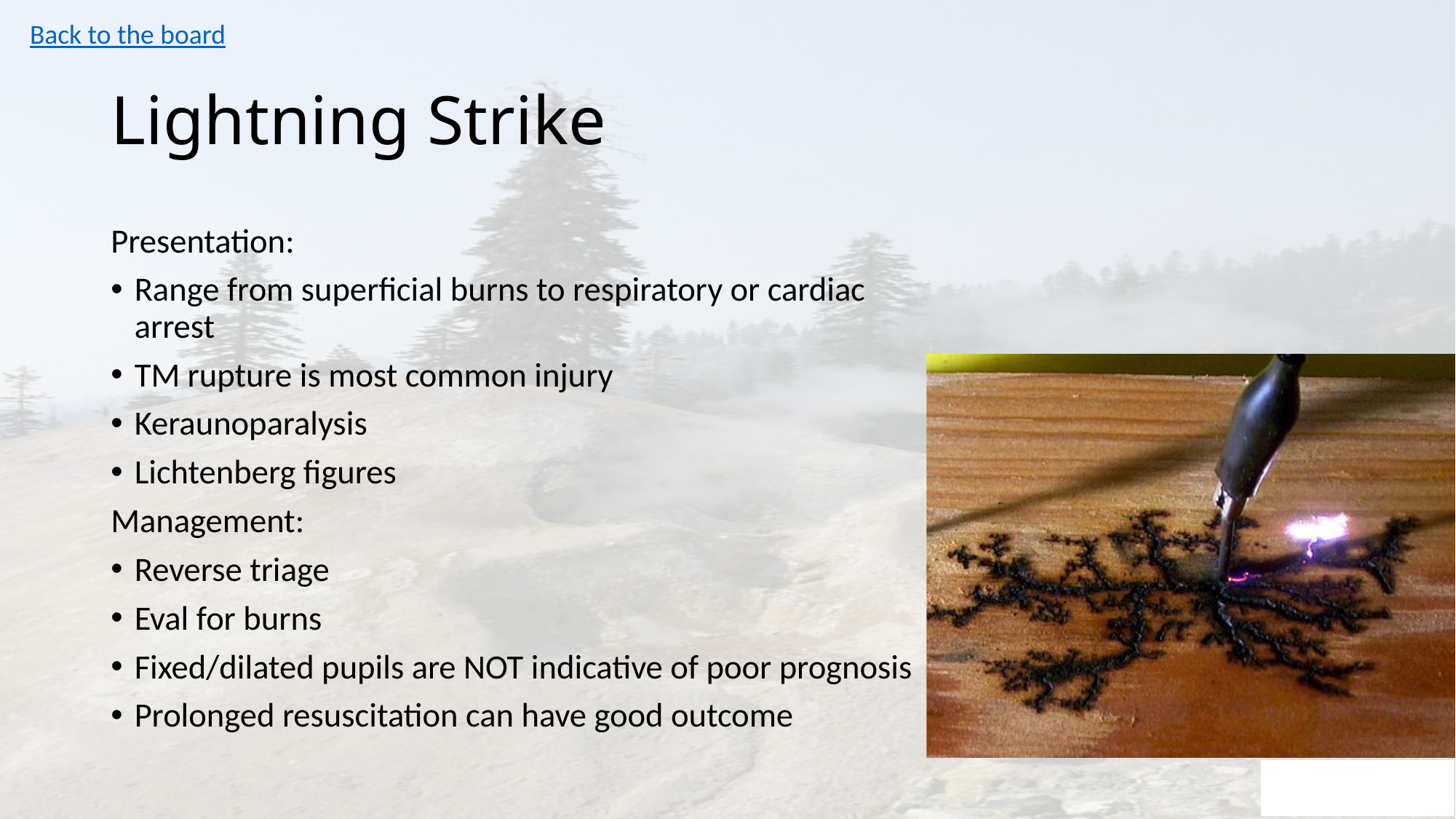

Back to the board
# Lightning Strike
Presentation:
Range from superficial burns to respiratory or cardiac arrest
TM rupture is most common injury
Keraunoparalysis
Lichtenberg figures
Management:
Reverse triage
Eval for burns
Fixed/dilated pupils are NOT indicative of poor prognosis
Prolonged resuscitation can have good outcome

## Slide 40
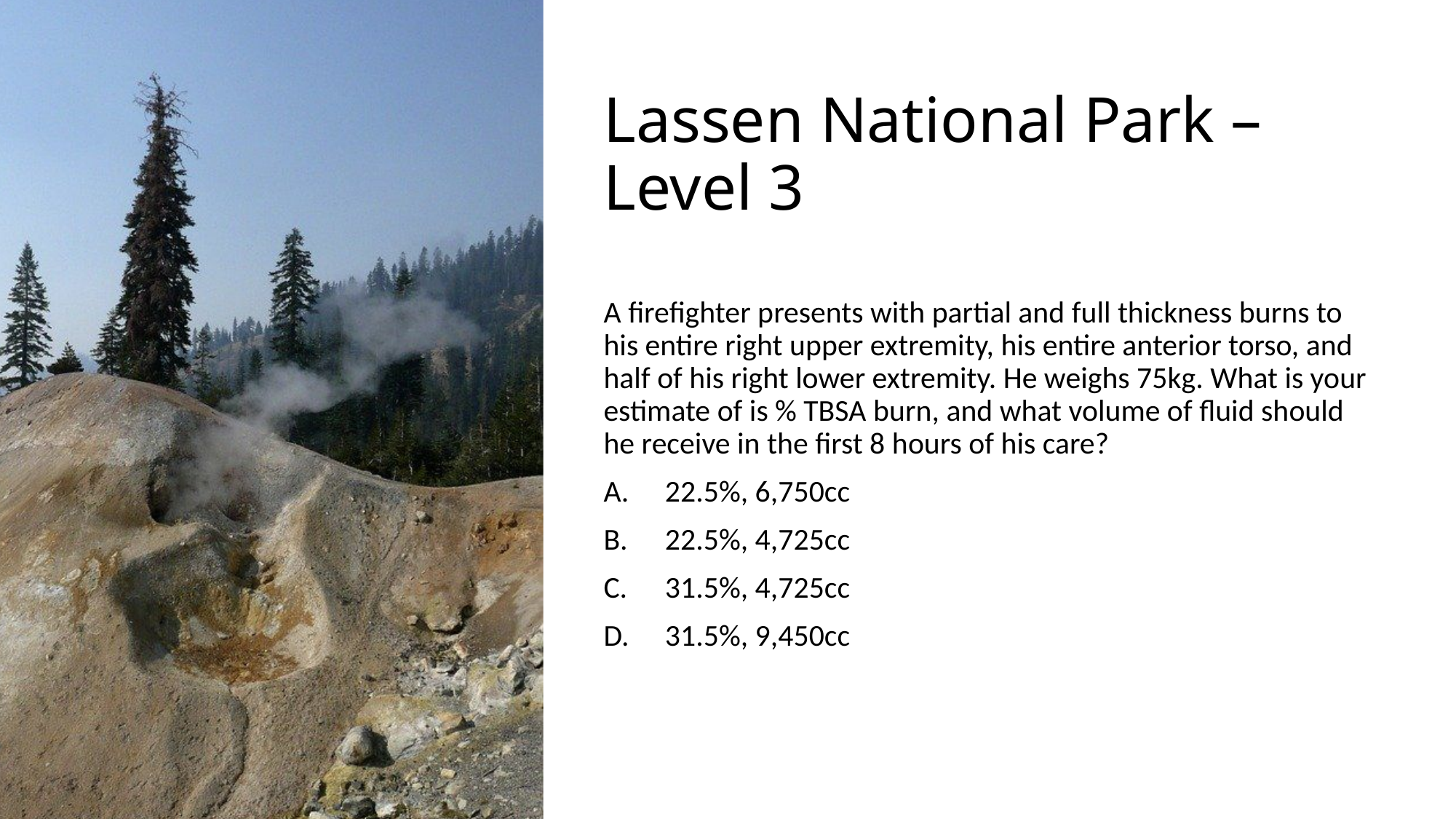

# Lassen National Park – Level 3
A firefighter presents with partial and full thickness burns to his entire right upper extremity, his entire anterior torso, and half of his right lower extremity. He weighs 75kg. What is your estimate of is % TBSA burn, and what volume of fluid should he receive in the first 8 hours of his care?
22.5%, 6,750cc
22.5%, 4,725cc
31.5%, 4,725cc
31.5%, 9,450cc

## Slide 41
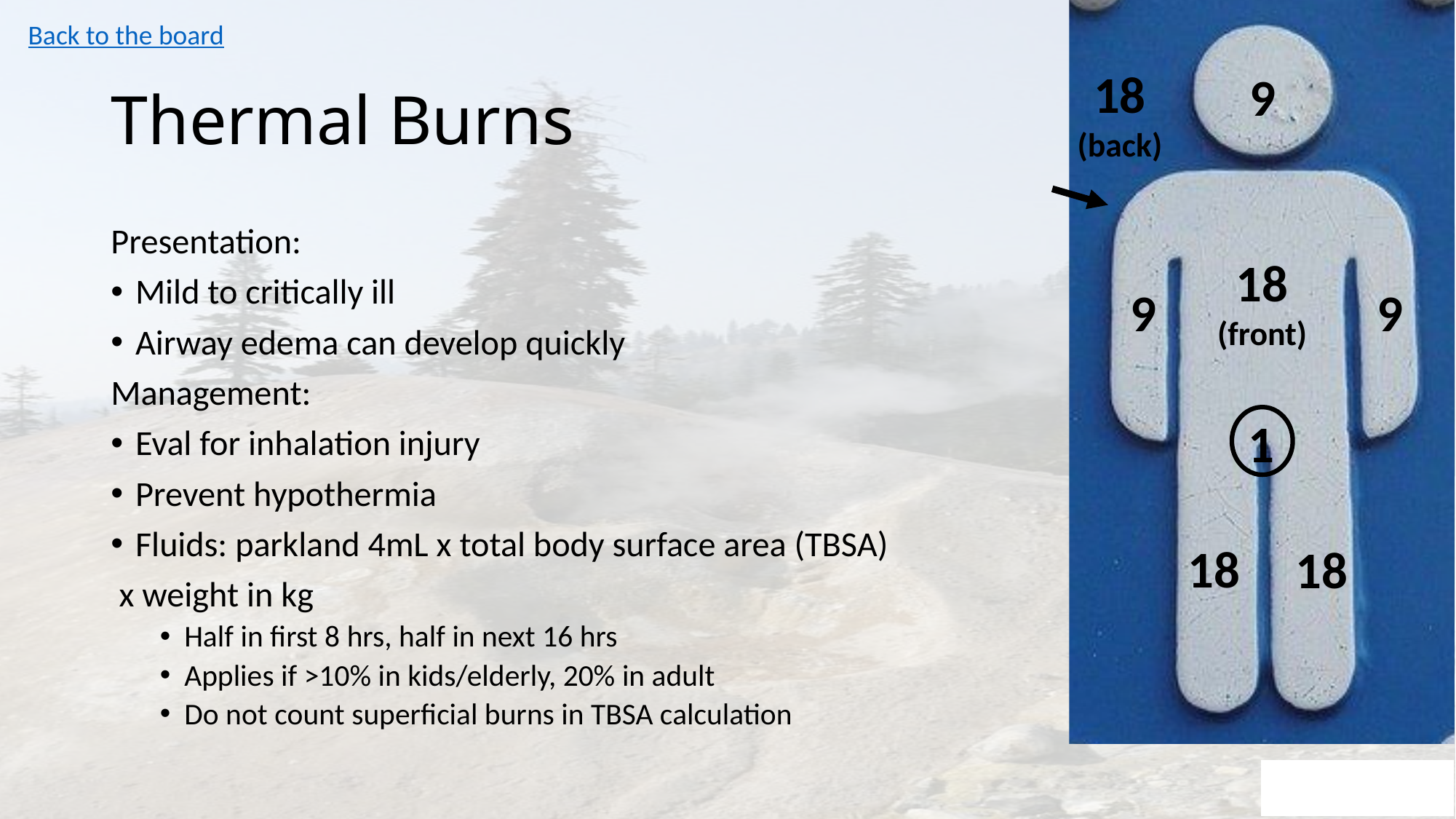

18
(back)
9
18
(front)
9
9
1
18
18
Back to the board
# Thermal Burns
Presentation:
Mild to critically ill
Airway edema can develop quickly
Management:
Eval for inhalation injury
Prevent hypothermia
Fluids: parkland 4mL x total body surface area (TBSA)
 x weight in kg
Half in first 8 hrs, half in next 16 hrs
Applies if >10% in kids/elderly, 20% in adult
Do not count superficial burns in TBSA calculation

## Slide 42
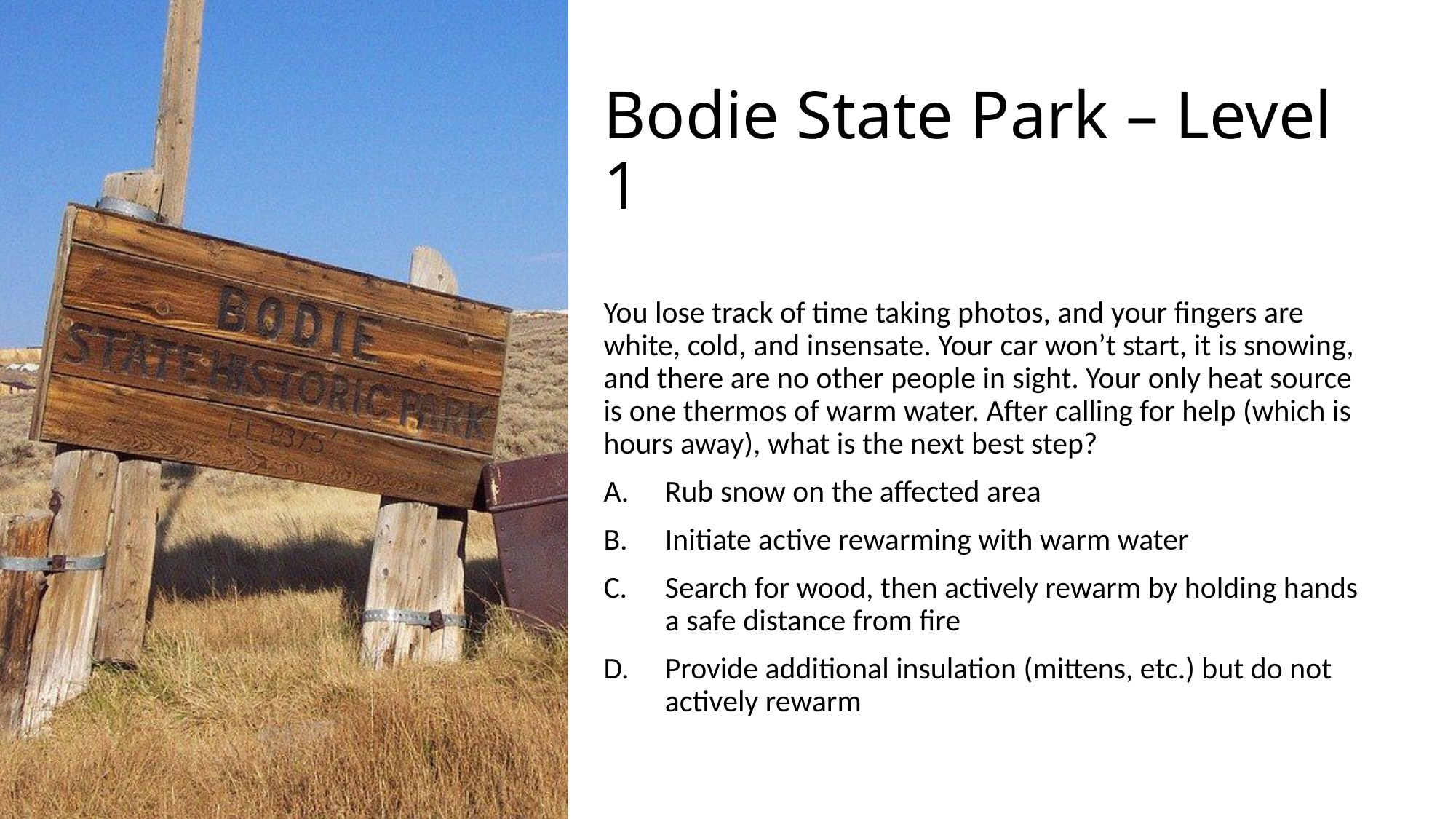

# Bodie State Park – Level 1
You lose track of time taking photos, and your fingers are white, cold, and insensate. Your car won’t start, it is snowing, and there are no other people in sight. Your only heat source is one thermos of warm water. After calling for help (which is hours away), what is the next best step?
Rub snow on the affected area
Initiate active rewarming with warm water
Search for wood, then actively rewarm by holding hands a safe distance from fire
Provide additional insulation (mittens, etc.) but do not actively rewarm

## Slide 43
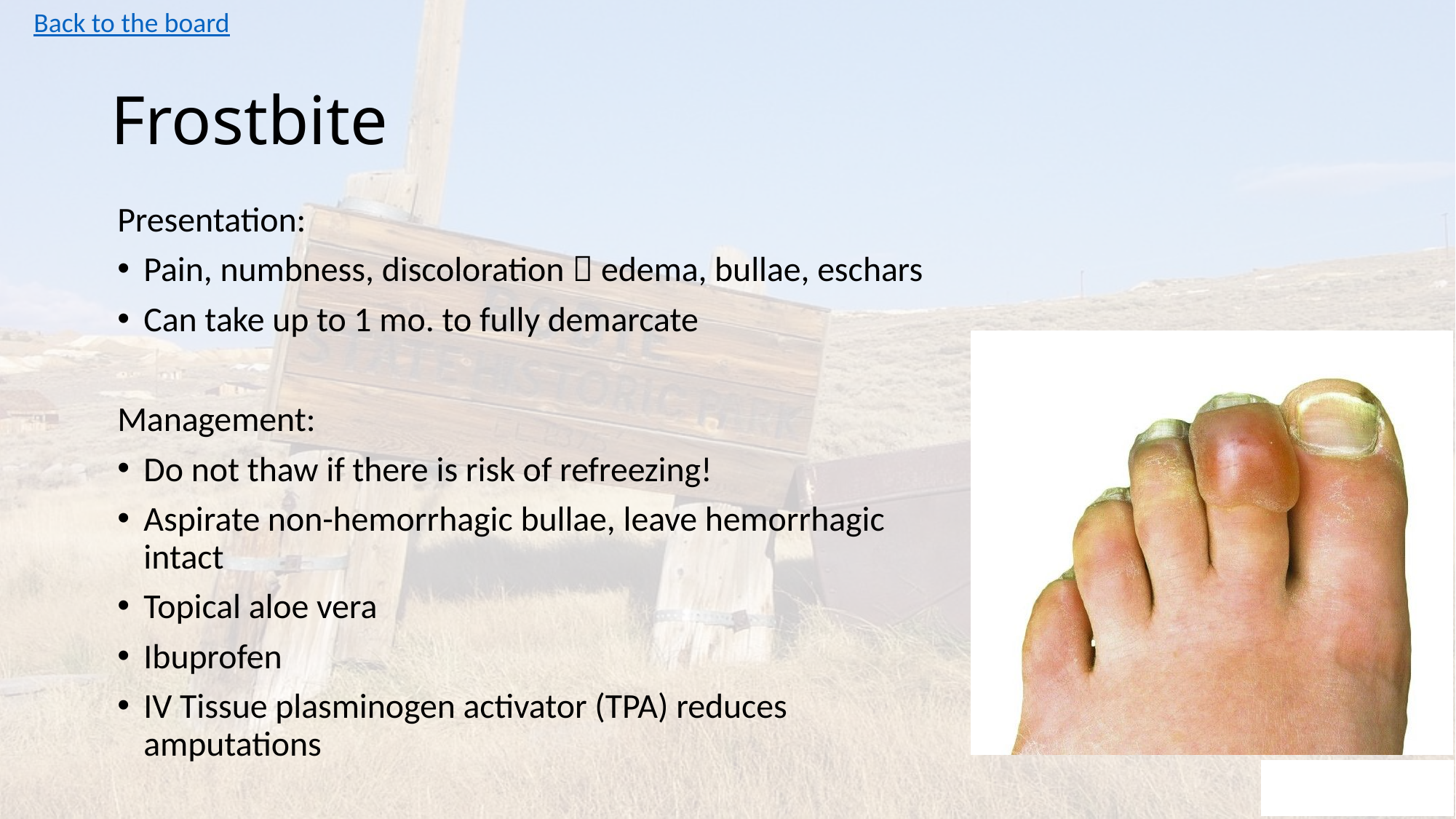

Back to the board
# Frostbite
Presentation:
Pain, numbness, discoloration  edema, bullae, eschars
Can take up to 1 mo. to fully demarcate
Management:
Do not thaw if there is risk of refreezing!
Aspirate non-hemorrhagic bullae, leave hemorrhagic intact
Topical aloe vera
Ibuprofen
IV Tissue plasminogen activator (TPA) reduces amputations

## Slide 44
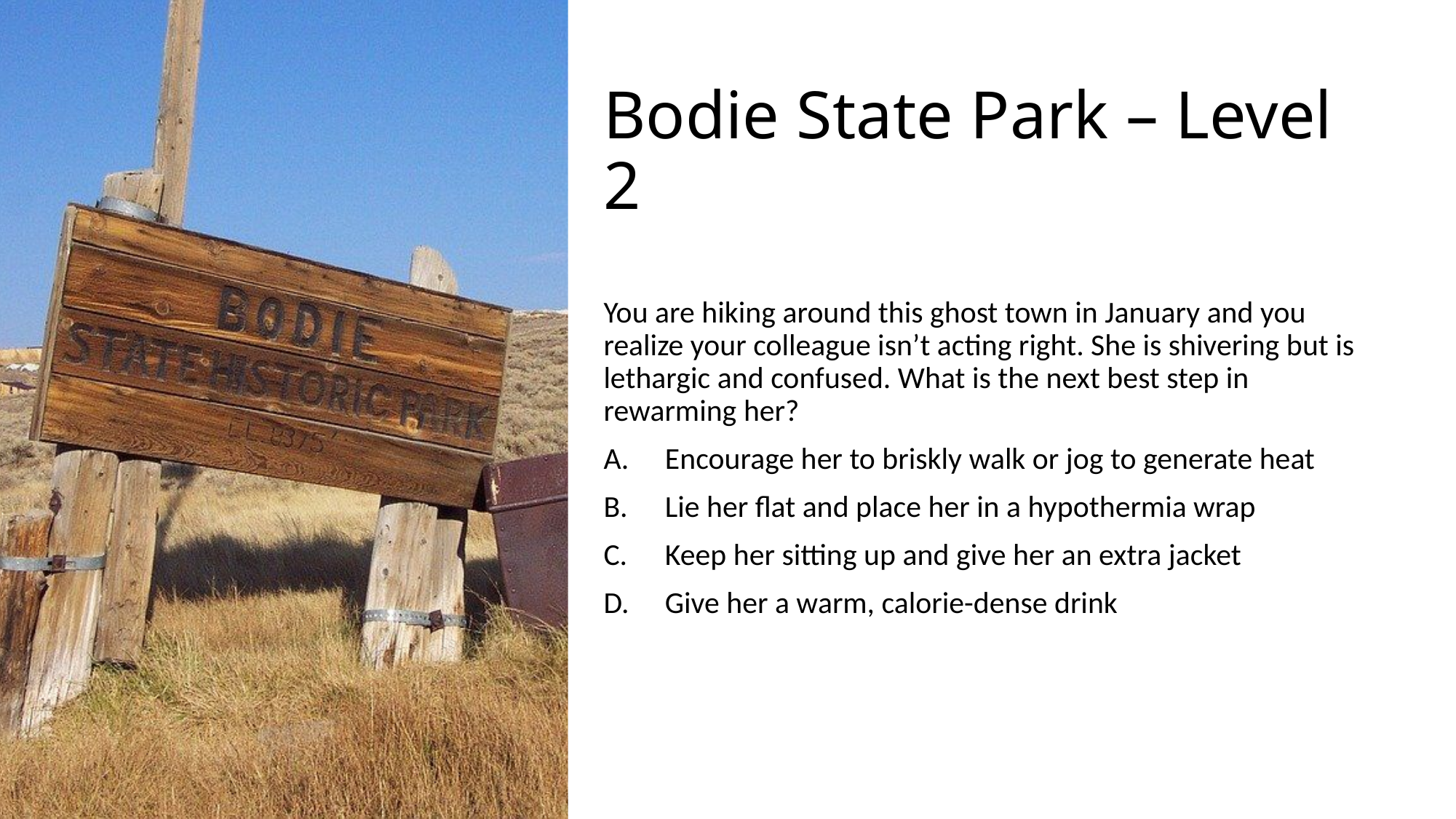

# Bodie State Park – Level 2
You are hiking around this ghost town in January and you realize your colleague isn’t acting right. She is shivering but is lethargic and confused. What is the next best step in rewarming her?
Encourage her to briskly walk or jog to generate heat
Lie her flat and place her in a hypothermia wrap
Keep her sitting up and give her an extra jacket
Give her a warm, calorie-dense drink

## Slide 45
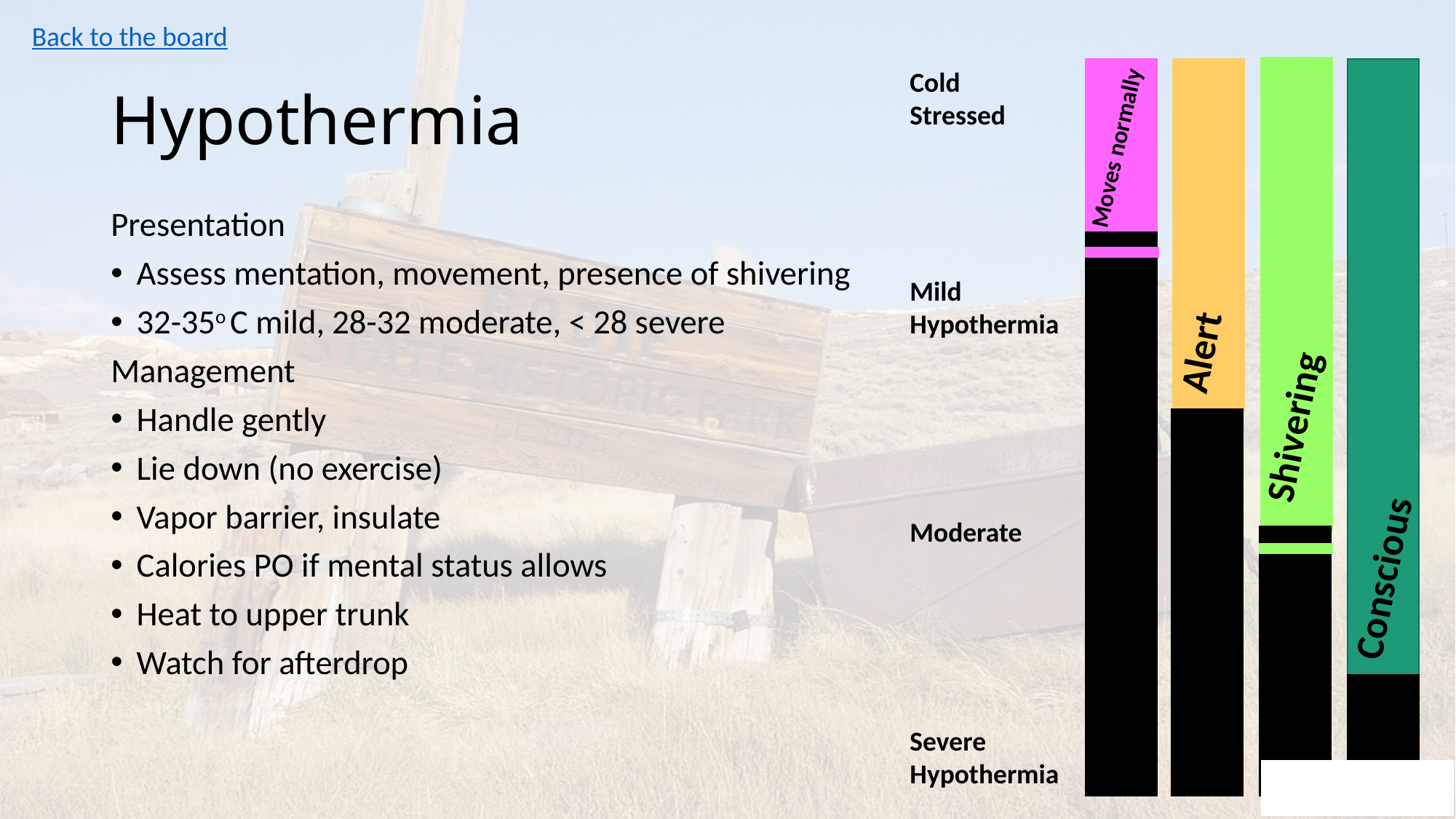

Moves normally
Back to the board
# Hypothermia
Shivering
Alert
Conscious
Cold Stressed
Presentation
Assess mentation, movement, presence of shivering
32-35o C mild, 28-32 moderate, < 28 severe
Management
Handle gently
Lie down (no exercise)
Vapor barrier, insulate
Calories PO if mental status allows
Heat to upper trunk
Watch for afterdrop
Mild Hypothermia
Moderate
Severe Hypothermia

## Slide 46
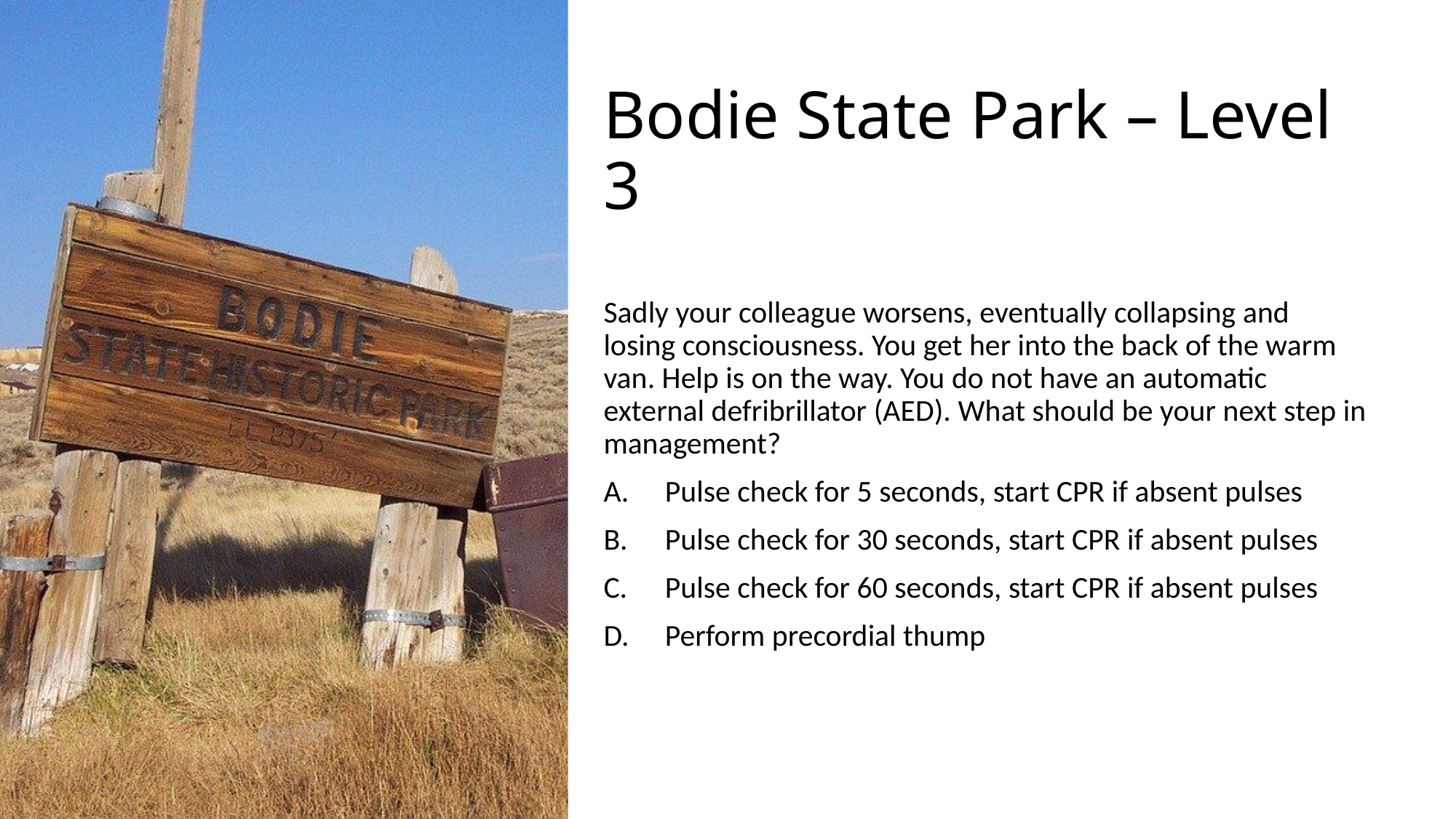

# Bodie State Park – Level 3
Sadly your colleague worsens, eventually collapsing and losing consciousness. You get her into the back of the warm van. Help is on the way. You do not have an automatic external defribrillator (AED). What should be your next step in management?
Pulse check for 5 seconds, start CPR if absent pulses
Pulse check for 30 seconds, start CPR if absent pulses
Pulse check for 60 seconds, start CPR if absent pulses
Perform precordial thump

## Slide 47
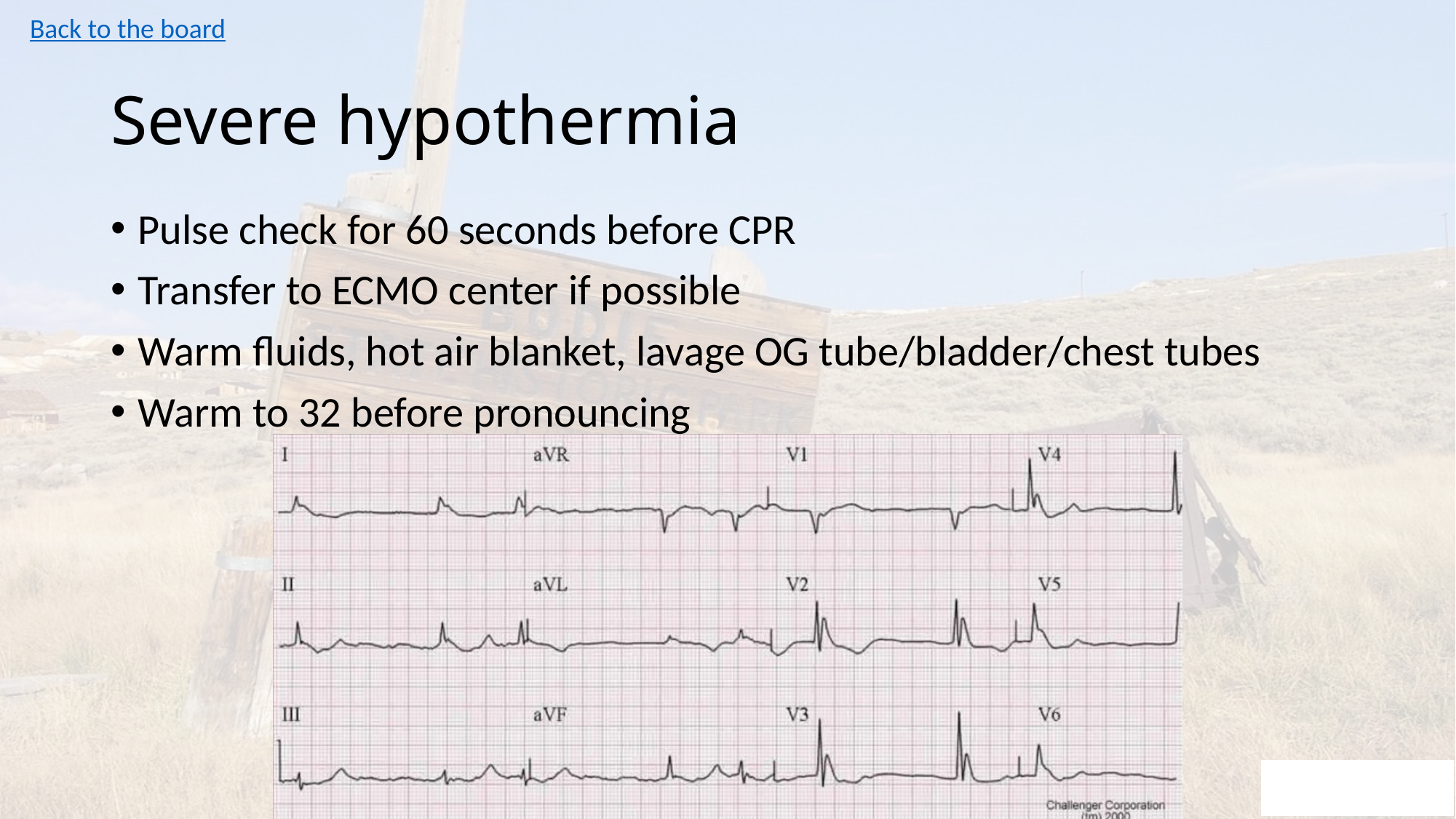

Back to the board
# Severe hypothermia
Pulse check for 60 seconds before CPR
Transfer to ECMO center if possible
Warm fluids, hot air blanket, lavage OG tube/bladder/chest tubes
Warm to 32 before pronouncing

## Slide 48
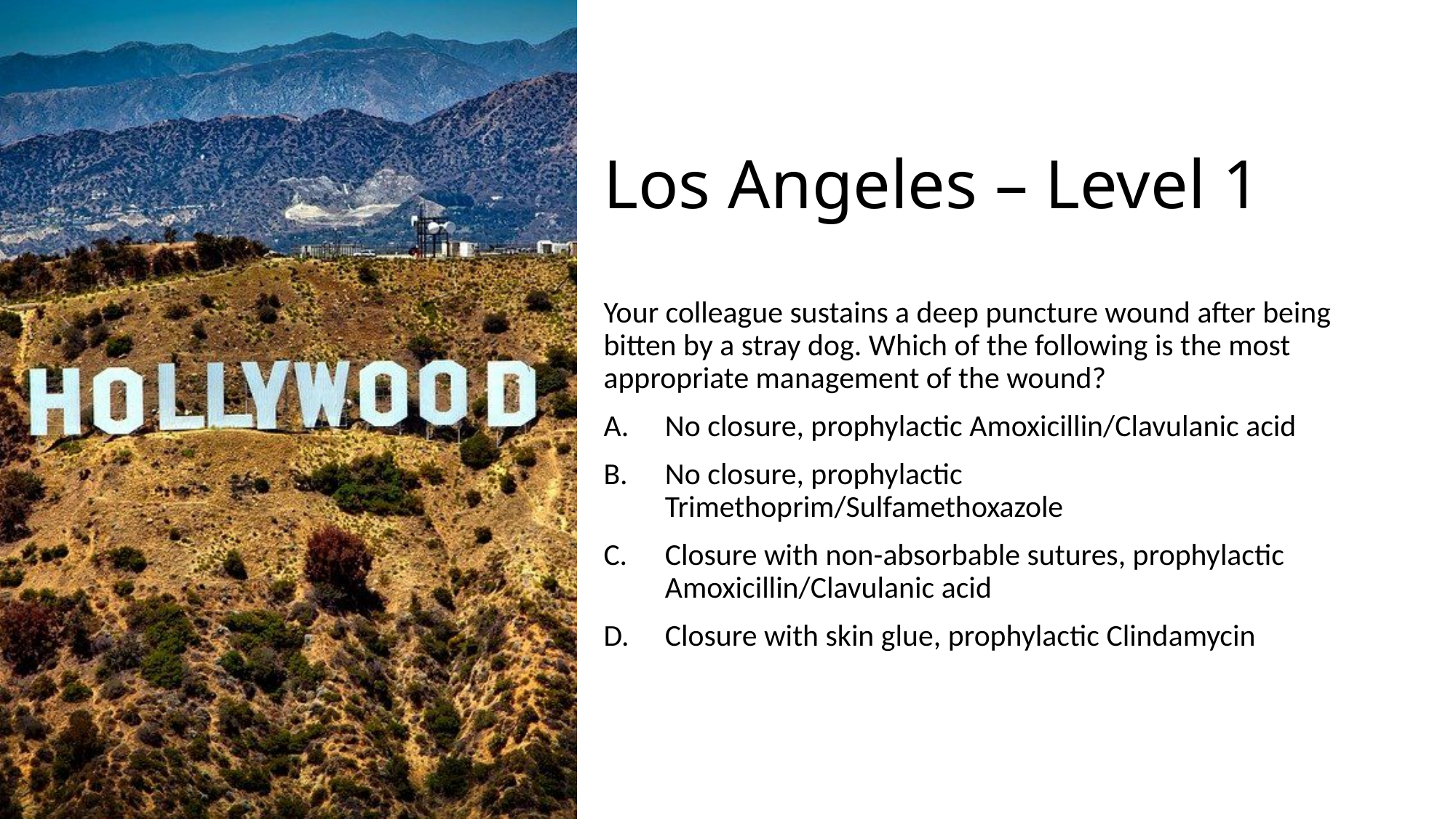

# Los Angeles – Level 1
Your colleague sustains a deep puncture wound after being bitten by a stray dog. Which of the following is the most appropriate management of the wound?
No closure, prophylactic Amoxicillin/Clavulanic acid
No closure, prophylactic Trimethoprim/Sulfamethoxazole
Closure with non-absorbable sutures, prophylactic Amoxicillin/Clavulanic acid
Closure with skin glue, prophylactic Clindamycin

## Slide 49
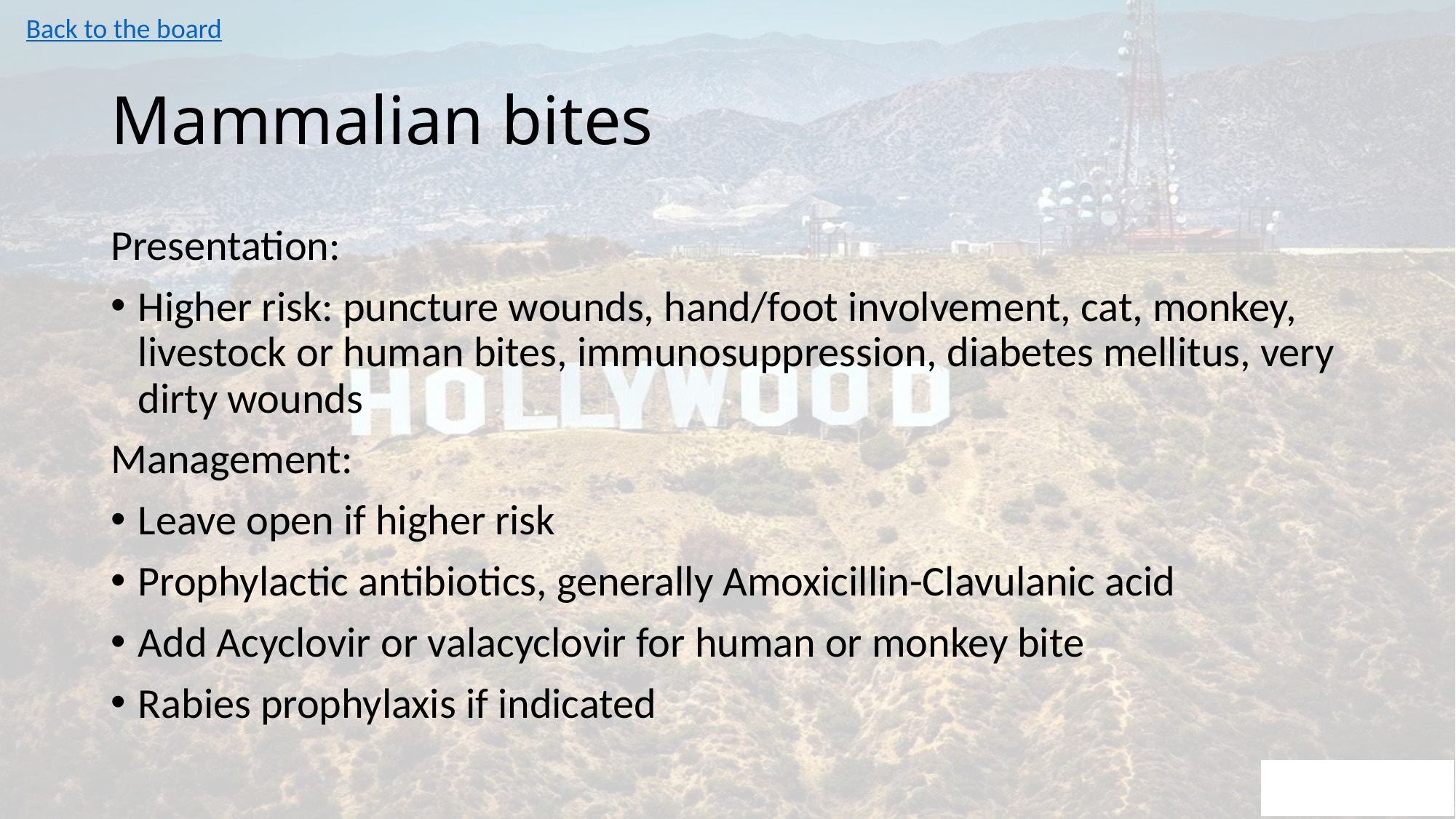

Back to the board
# Mammalian bites
Presentation:
Higher risk: puncture wounds, hand/foot involvement, cat, monkey, livestock or human bites, immunosuppression, diabetes mellitus, very dirty wounds
Management:
Leave open if higher risk
Prophylactic antibiotics, generally Amoxicillin-Clavulanic acid
Add Acyclovir or valacyclovir for human or monkey bite
Rabies prophylaxis if indicated

## Slide 50
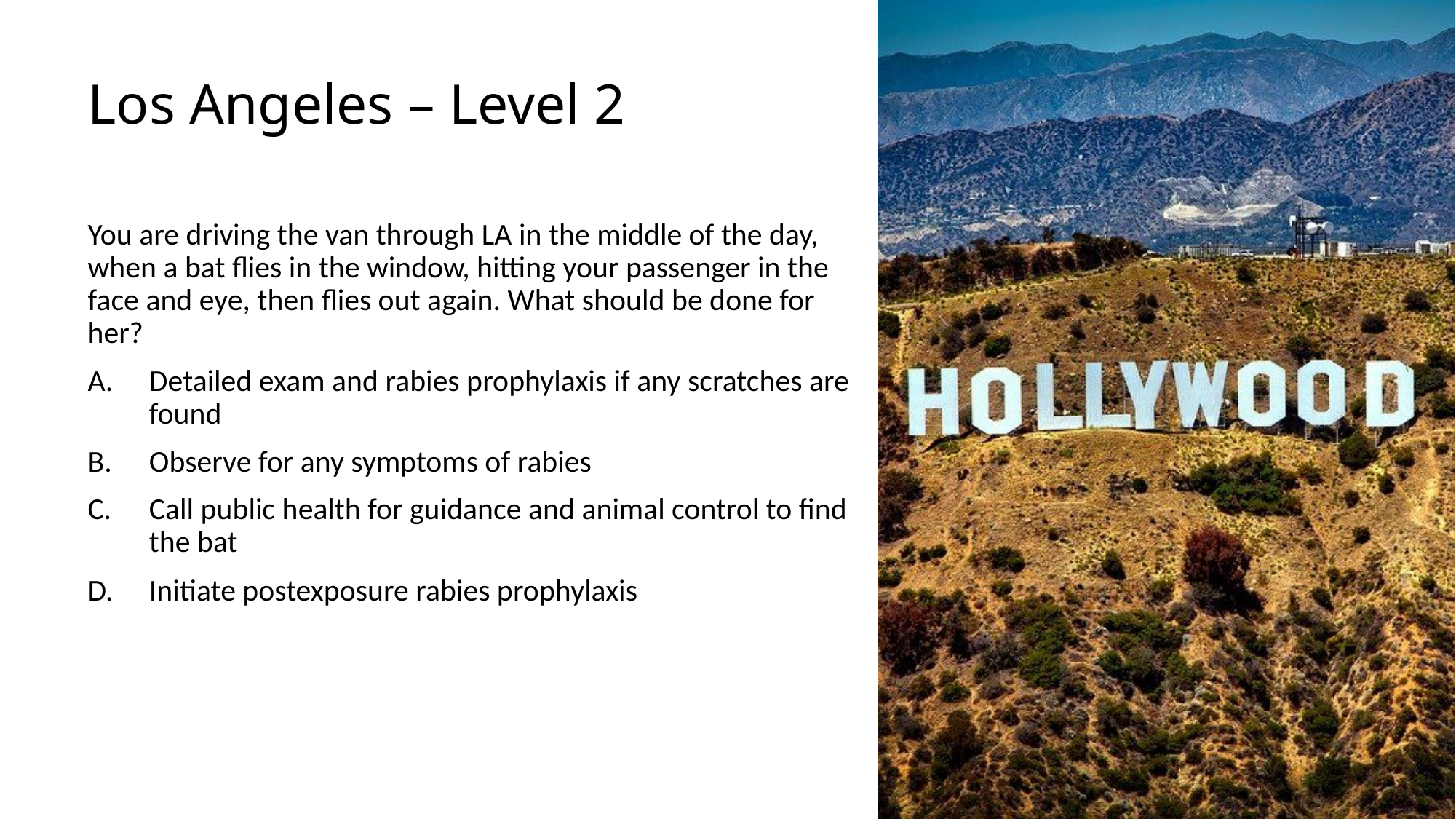

# Los Angeles – Level 2
You are driving the van through LA in the middle of the day, when a bat flies in the window, hitting your passenger in the face and eye, then flies out again. What should be done for her?
Detailed exam and rabies prophylaxis if any scratches are found
Observe for any symptoms of rabies
Call public health for guidance and animal control to find the bat
Initiate postexposure rabies prophylaxis

## Slide 51
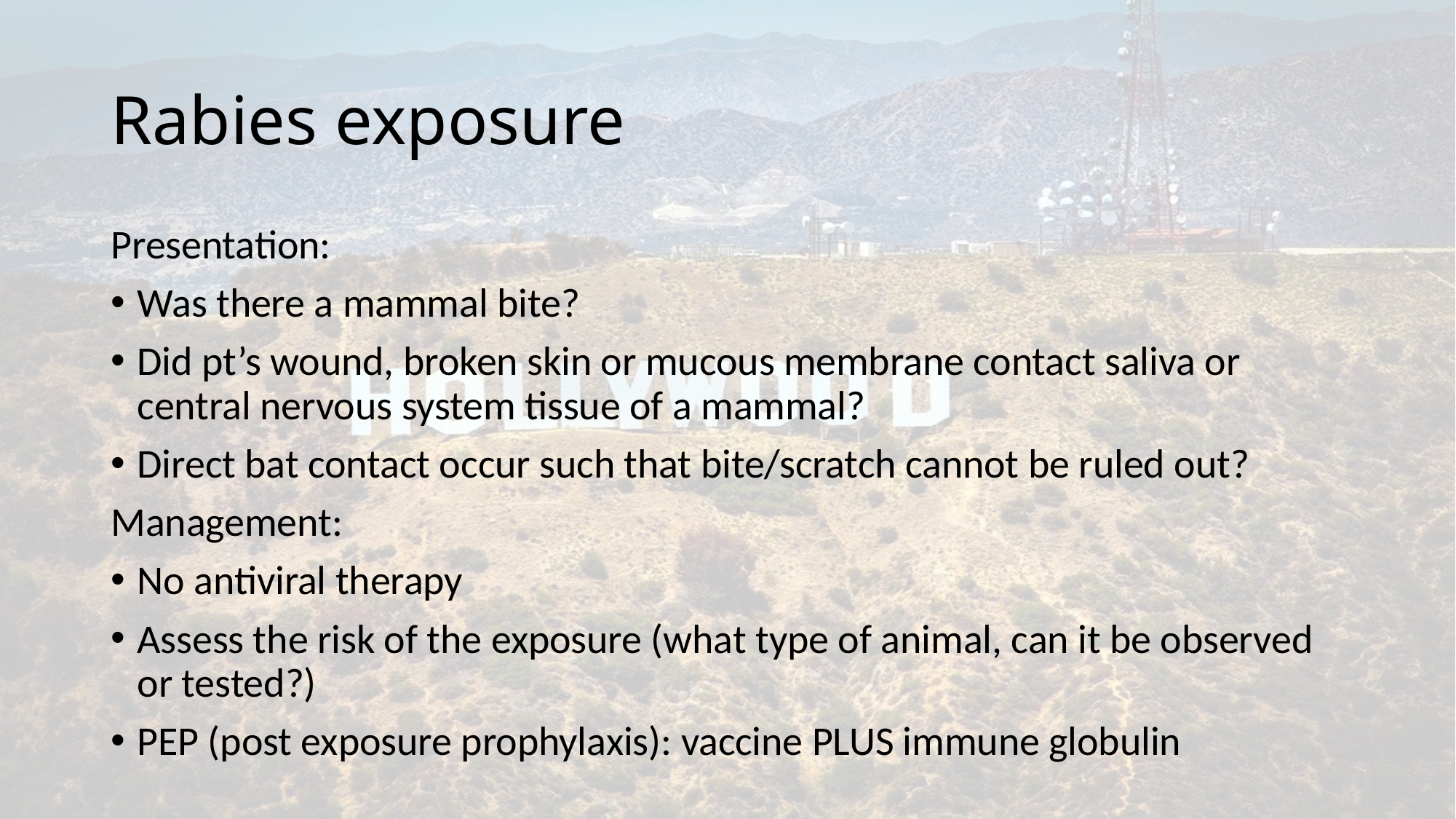

# Rabies exposure
Presentation:
Was there a mammal bite?
Did pt’s wound, broken skin or mucous membrane contact saliva or central nervous system tissue of a mammal?
Direct bat contact occur such that bite/scratch cannot be ruled out?
Management:
No antiviral therapy
Assess the risk of the exposure (what type of animal, can it be observed or tested?)
PEP (post exposure prophylaxis): vaccine PLUS immune globulin

## Slide 52
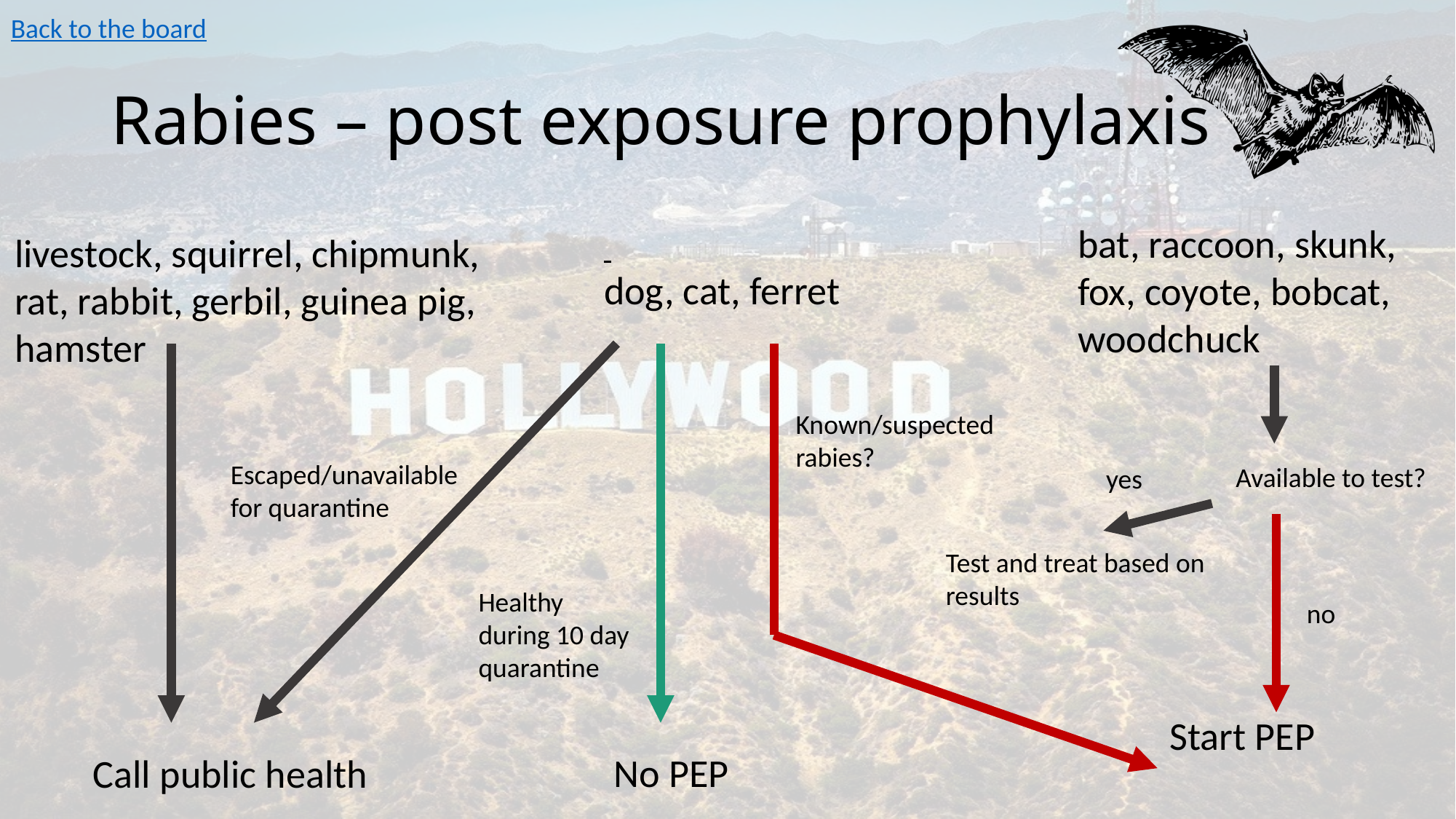

Back to the board
 Bat Dracula animal mammal biology: In: pixabay. https://pixabay.com/vectors/bat-dracula-animal-mammal-biology-147038/
# Rabies – post exposure prophylaxis
bat, raccoon, skunk, fox, coyote, bobcat, woodchuck
dog, cat, ferret
livestock, squirrel, chipmunk, rat, rabbit, gerbil, guinea pig, hamster
Known/suspected rabies?
Escaped/unavailable for quarantine
Available to test?
yes
Test and treat based on results
Healthy during 10 day quarantine
no
Start PEP
No PEP
Call public health
Adapted from the Centers for disease control

## Slide 53
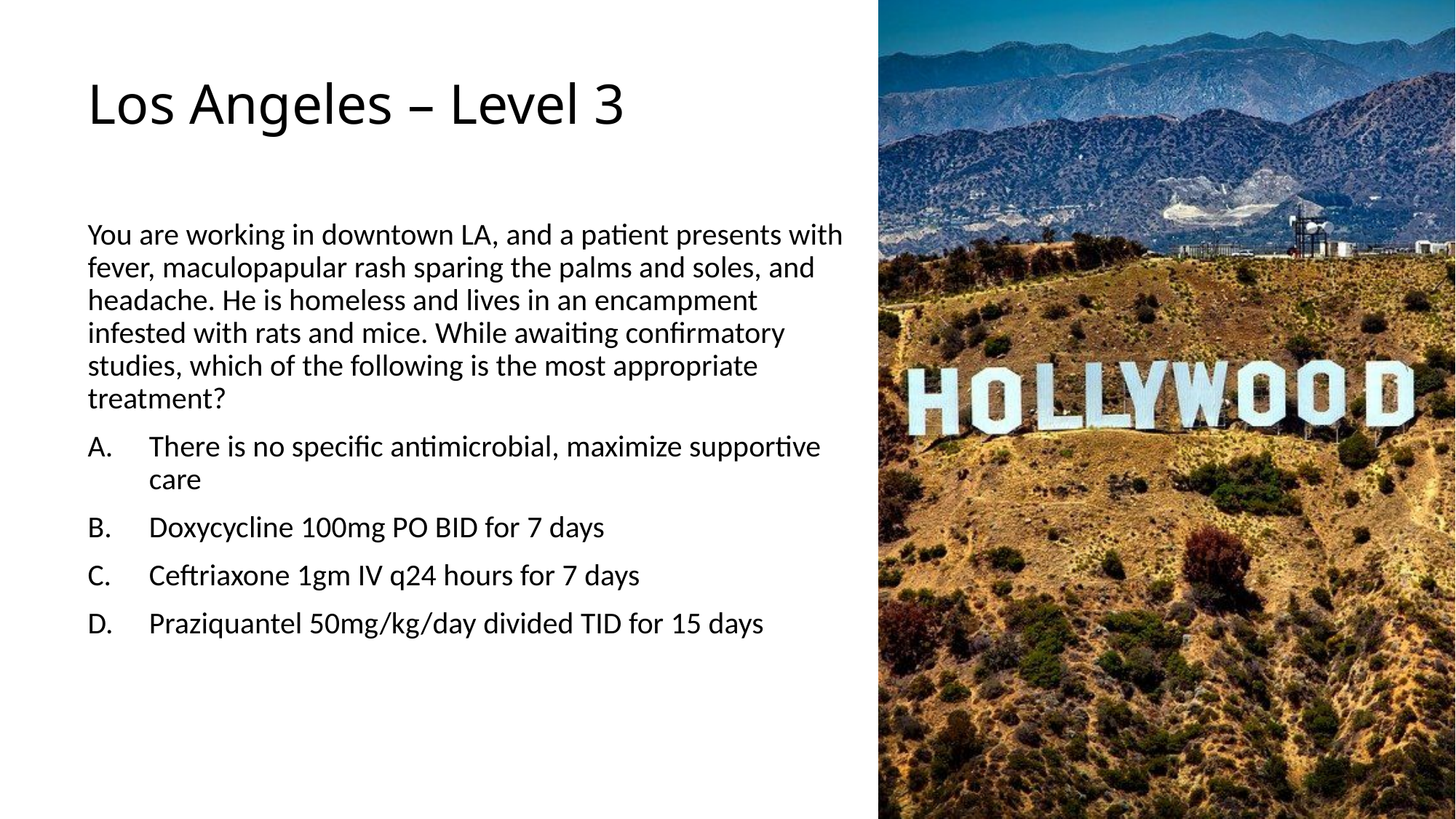

# Los Angeles – Level 3
You are working in downtown LA, and a patient presents with fever, maculopapular rash sparing the palms and soles, and headache. He is homeless and lives in an encampment infested with rats and mice. While awaiting confirmatory studies, which of the following is the most appropriate treatment?
There is no specific antimicrobial, maximize supportive care
Doxycycline 100mg PO BID for 7 days
Ceftriaxone 1gm IV q24 hours for 7 days
Praziquantel 50mg/kg/day divided TID for 15 days

## Slide 54
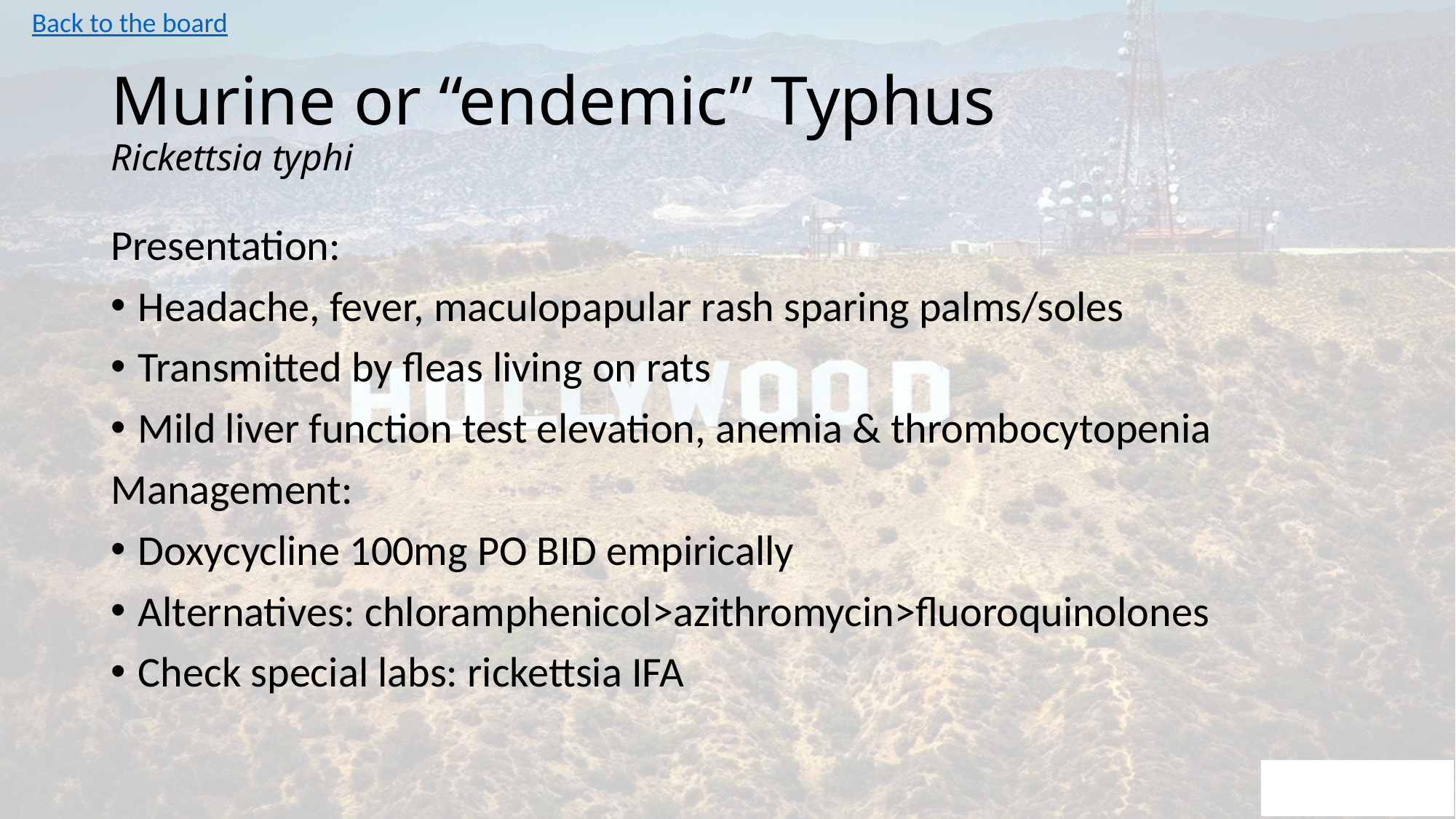

Back to the board
# Murine or “endemic” Typhus Rickettsia typhi
Presentation:
Headache, fever, maculopapular rash sparing palms/soles
Transmitted by fleas living on rats
Mild liver function test elevation, anemia & thrombocytopenia
Management:
Doxycycline 100mg PO BID empirically
Alternatives: chloramphenicol>azithromycin>fluoroquinolones
Check special labs: rickettsia IFA

## Slide 55
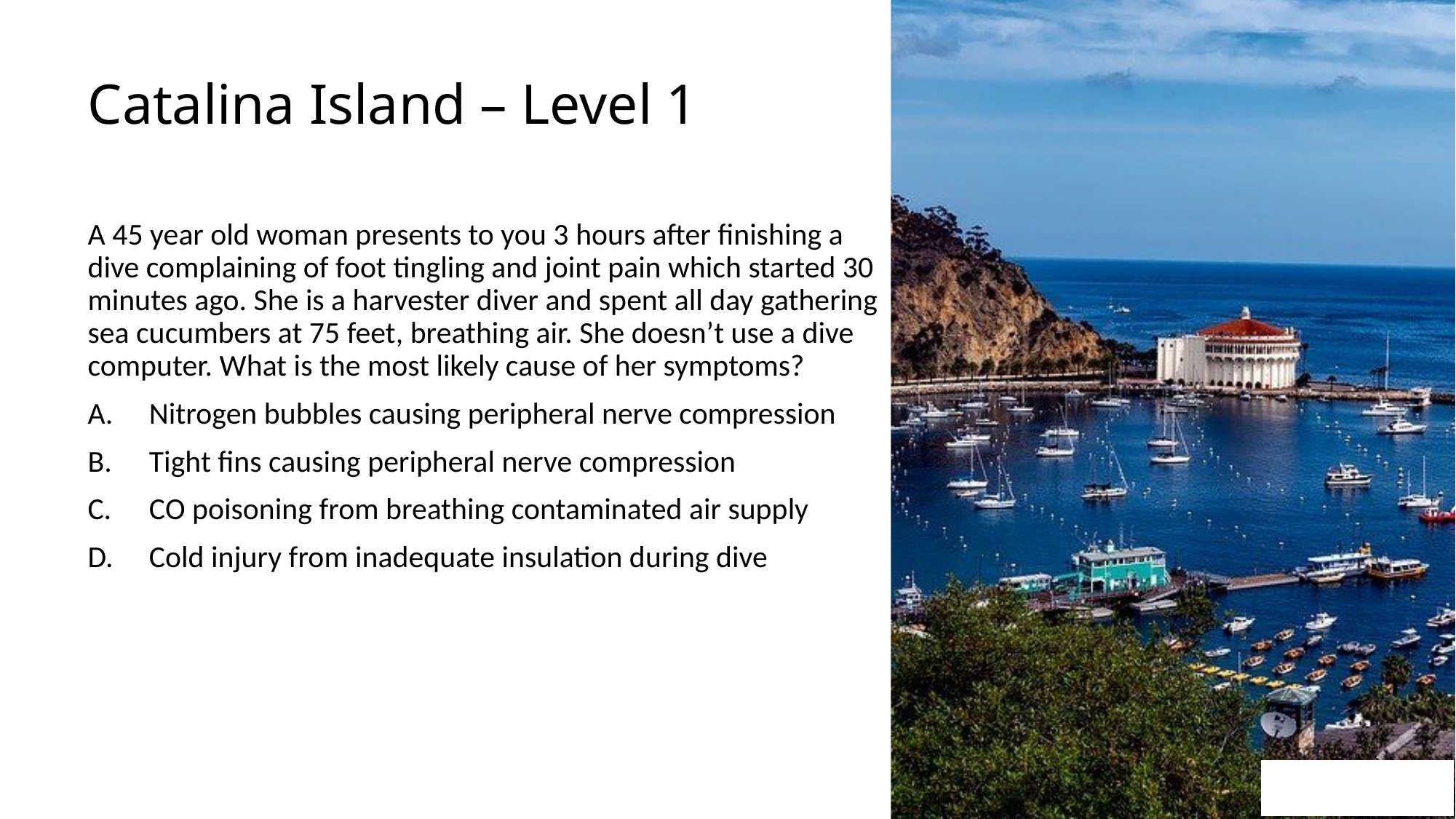

# Catalina Island – Level 1
A 45 year old woman presents to you 3 hours after finishing a dive complaining of foot tingling and joint pain which started 30 minutes ago. She is a harvester diver and spent all day gathering sea cucumbers at 75 feet, breathing air. She doesn’t use a dive computer. What is the most likely cause of her symptoms?
Nitrogen bubbles causing peripheral nerve compression
Tight fins causing peripheral nerve compression
CO poisoning from breathing contaminated air supply
Cold injury from inadequate insulation during dive

## Slide 56
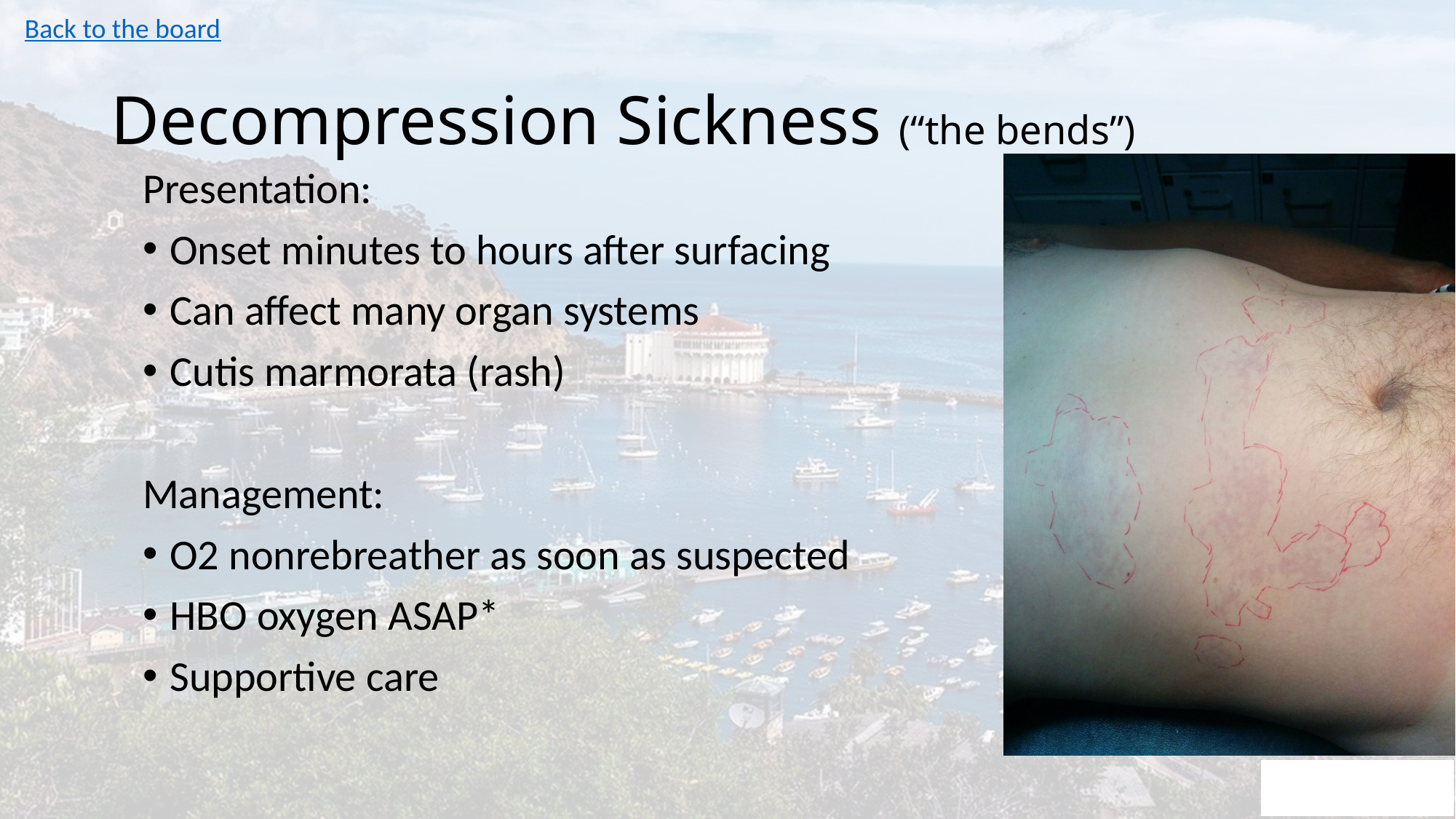

Back to the board
# Decompression Sickness (“the bends”)
Presentation:
Onset minutes to hours after surfacing
Can affect many organ systems
Cutis marmorata (rash)
Management:
O2 nonrebreather as soon as suspected
HBO oxygen ASAP*
Supportive care

## Slide 57
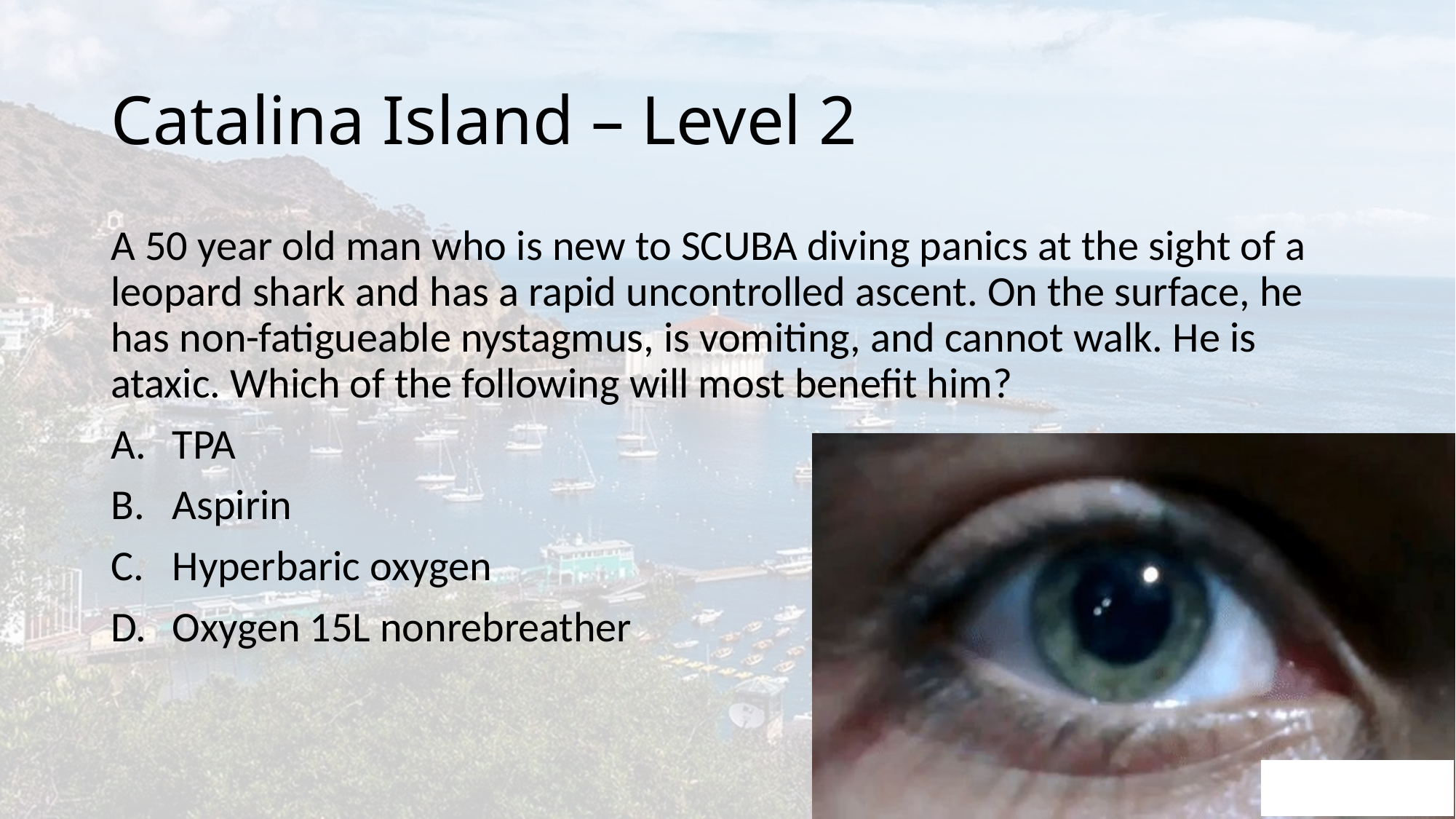

# Catalina Island – Level 2
A 50 year old man who is new to SCUBA diving panics at the sight of a leopard shark and has a rapid uncontrolled ascent. On the surface, he has non-fatigueable nystagmus, is vomiting, and cannot walk. He is ataxic. Which of the following will most benefit him?
TPA
Aspirin
Hyperbaric oxygen
Oxygen 15L nonrebreather

## Slide 58
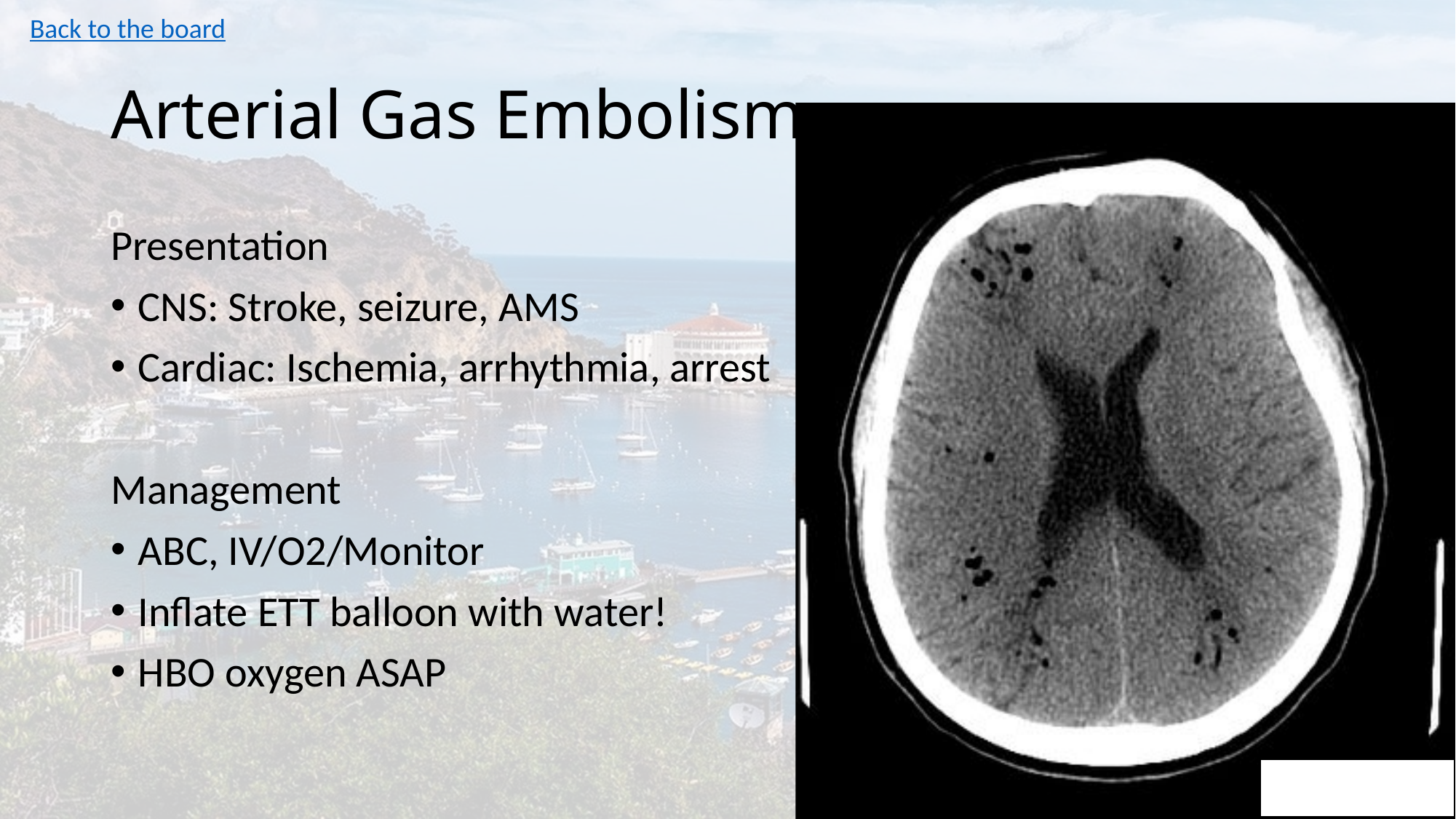

Back to the board
# Arterial Gas Embolism
Presentation
CNS: Stroke, seizure, AMS
Cardiac: Ischemia, arrhythmia, arrest
Management
ABC, IV/O2/Monitor
Inflate ETT balloon with water!
HBO oxygen ASAP

## Slide 59
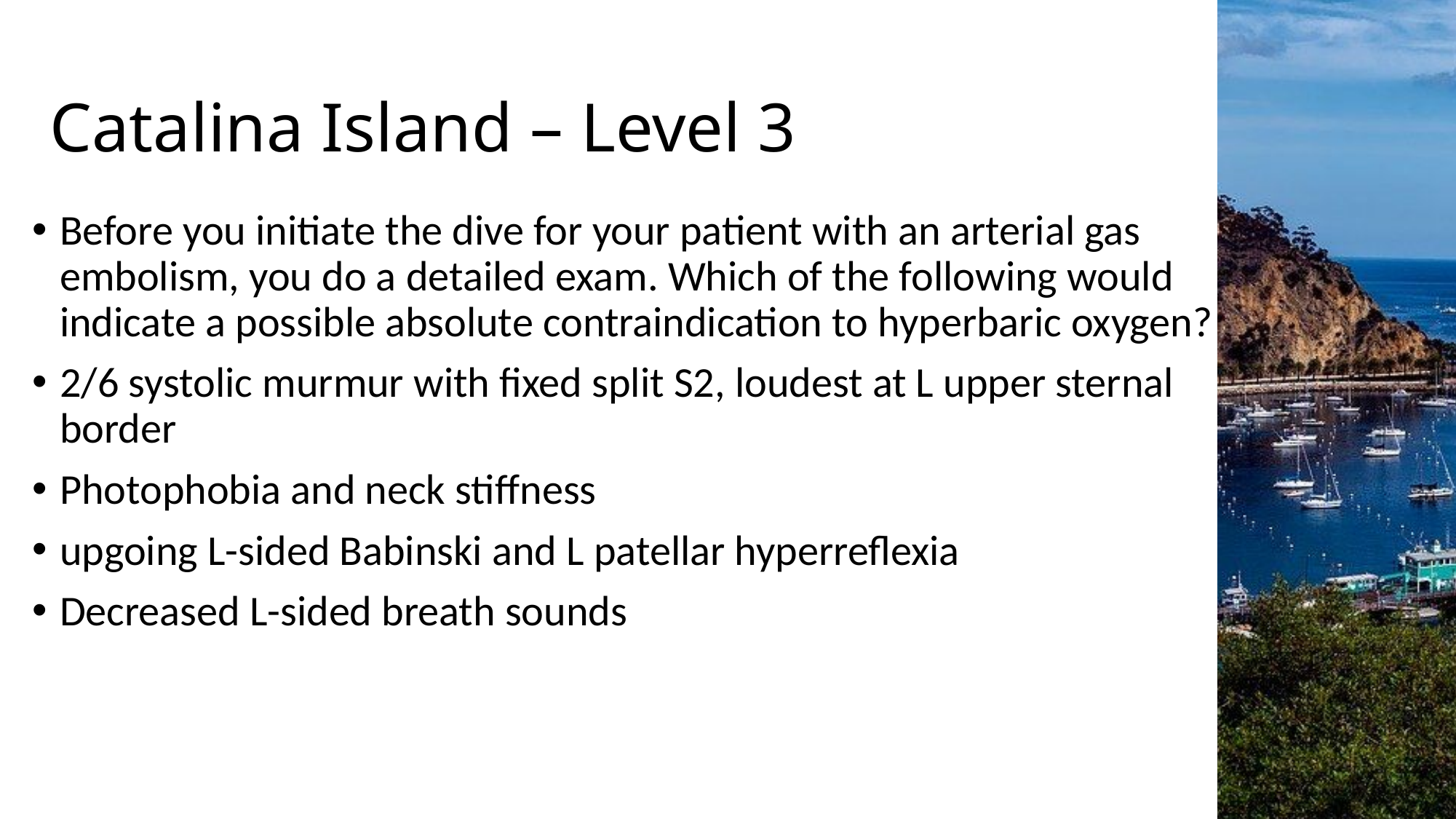

# Catalina Island – Level 3
Before you initiate the dive for your patient with an arterial gas embolism, you do a detailed exam. Which of the following would indicate a possible absolute contraindication to hyperbaric oxygen?
2/6 systolic murmur with fixed split S2, loudest at L upper sternal border
Photophobia and neck stiffness
upgoing L-sided Babinski and L patellar hyperreflexia
Decreased L-sided breath sounds

## Slide 60
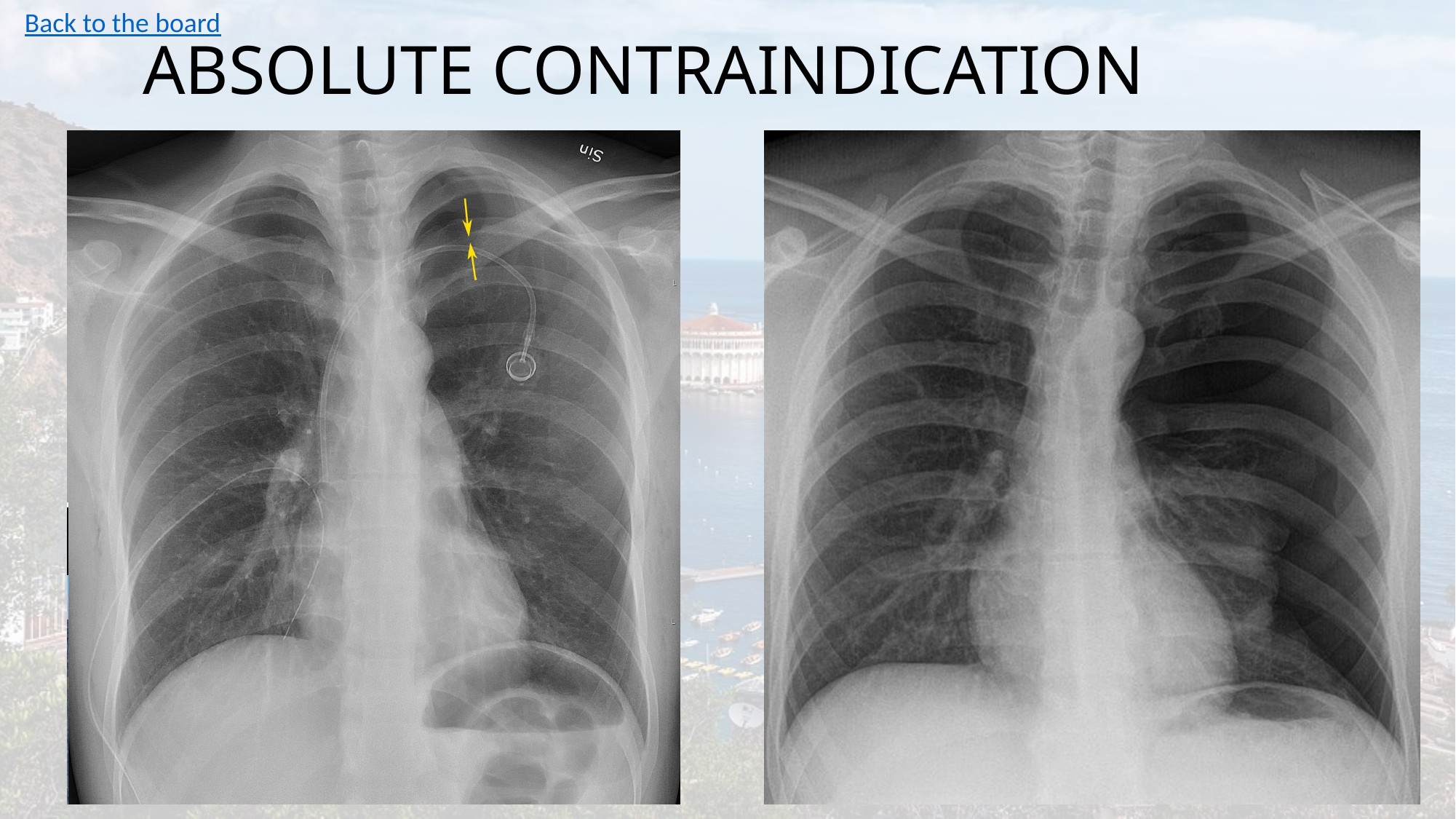

Back to the board
# ABSOLUTE CONTRAINDICATION

## Slide 61
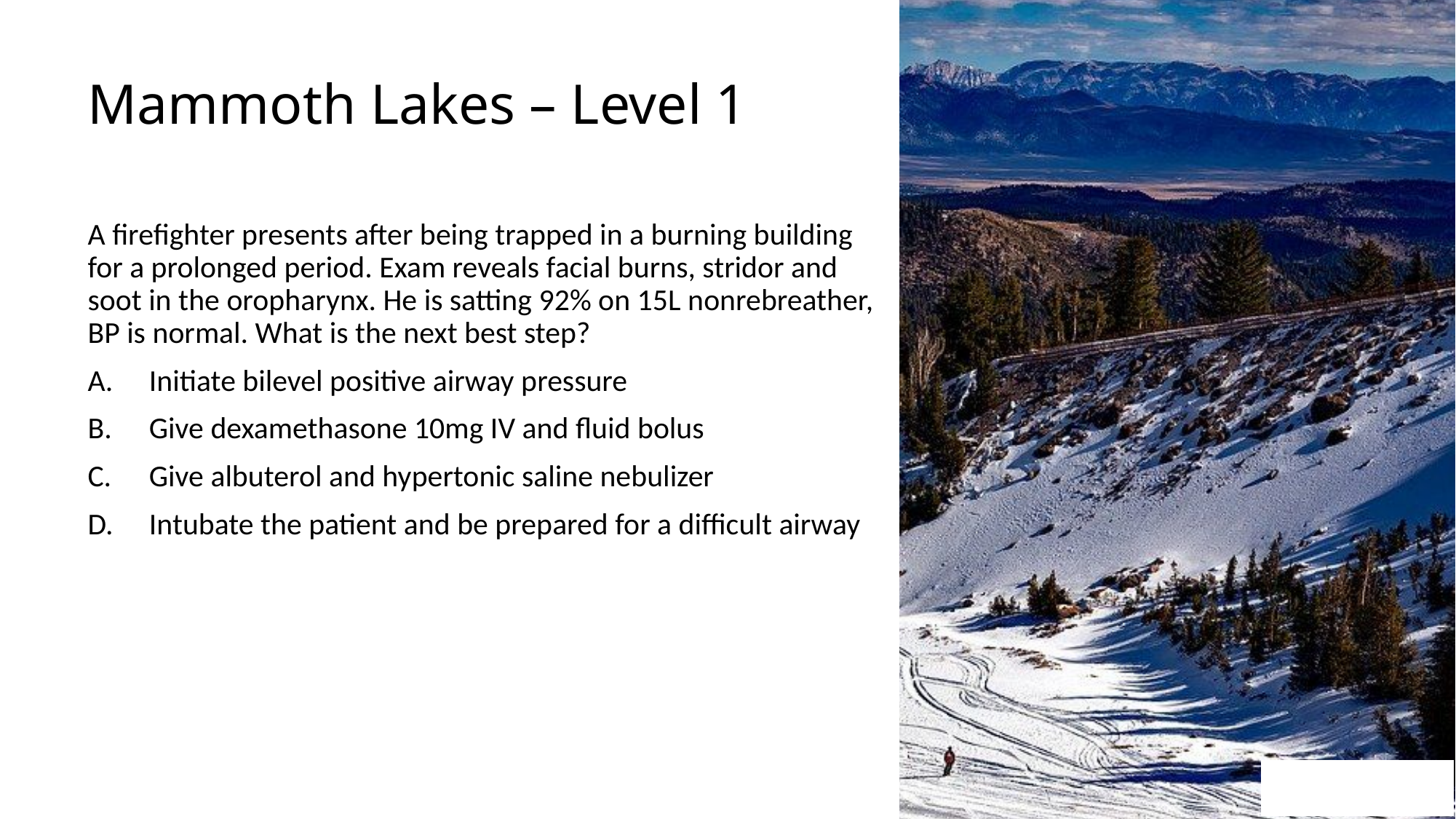

# Mammoth Lakes – Level 1
A firefighter presents after being trapped in a burning building for a prolonged period. Exam reveals facial burns, stridor and soot in the oropharynx. He is satting 92% on 15L nonrebreather, BP is normal. What is the next best step?
Initiate bilevel positive airway pressure
Give dexamethasone 10mg IV and fluid bolus
Give albuterol and hypertonic saline nebulizer
Intubate the patient and be prepared for a difficult airway

## Slide 62
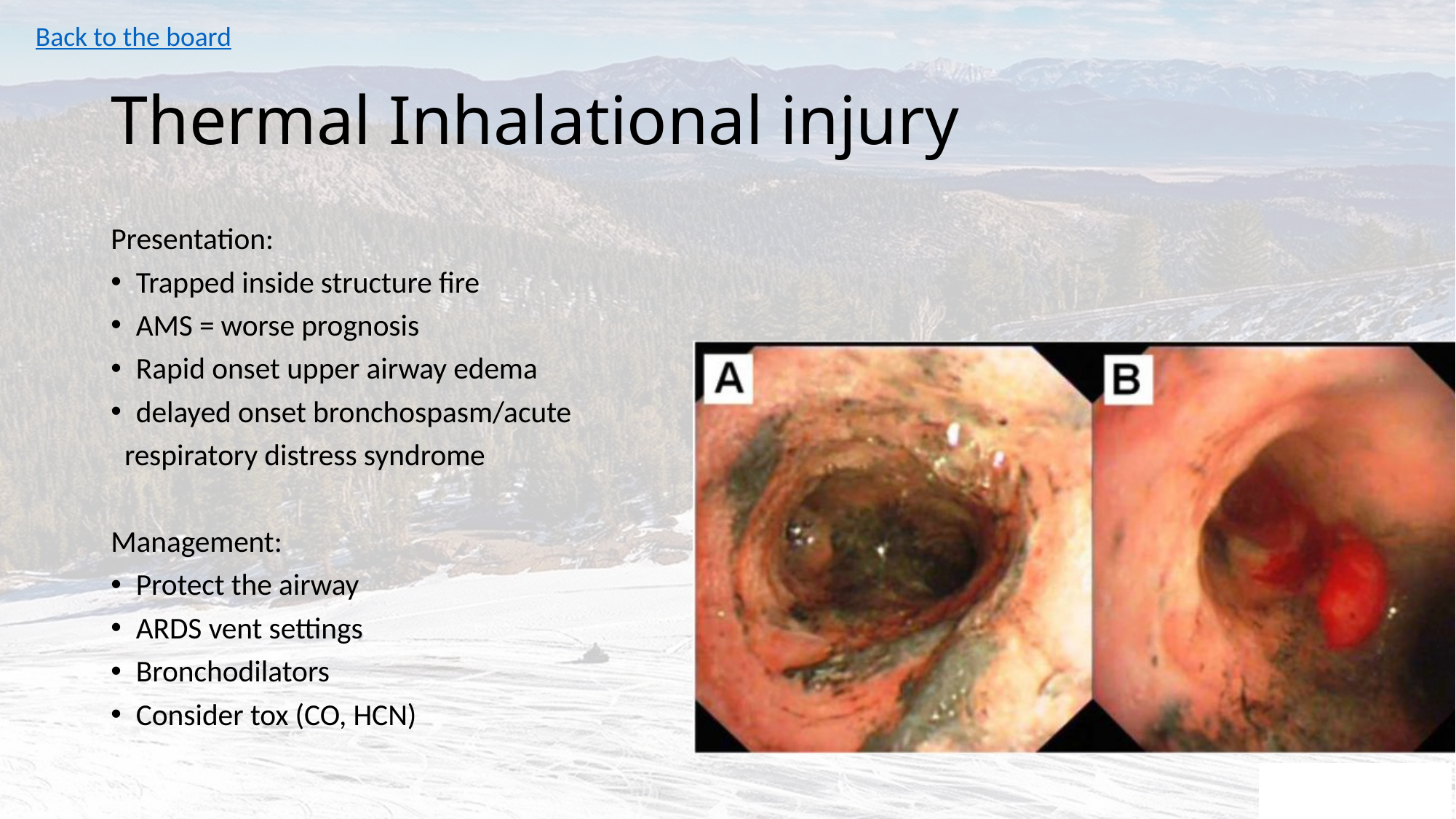

Back to the board
# Thermal Inhalational injury
Presentation:
Trapped inside structure fire
AMS = worse prognosis
Rapid onset upper airway edema
delayed onset bronchospasm/acute
 respiratory distress syndrome
Management:
Protect the airway
ARDS vent settings
Bronchodilators
Consider tox (CO, HCN)

## Slide 63
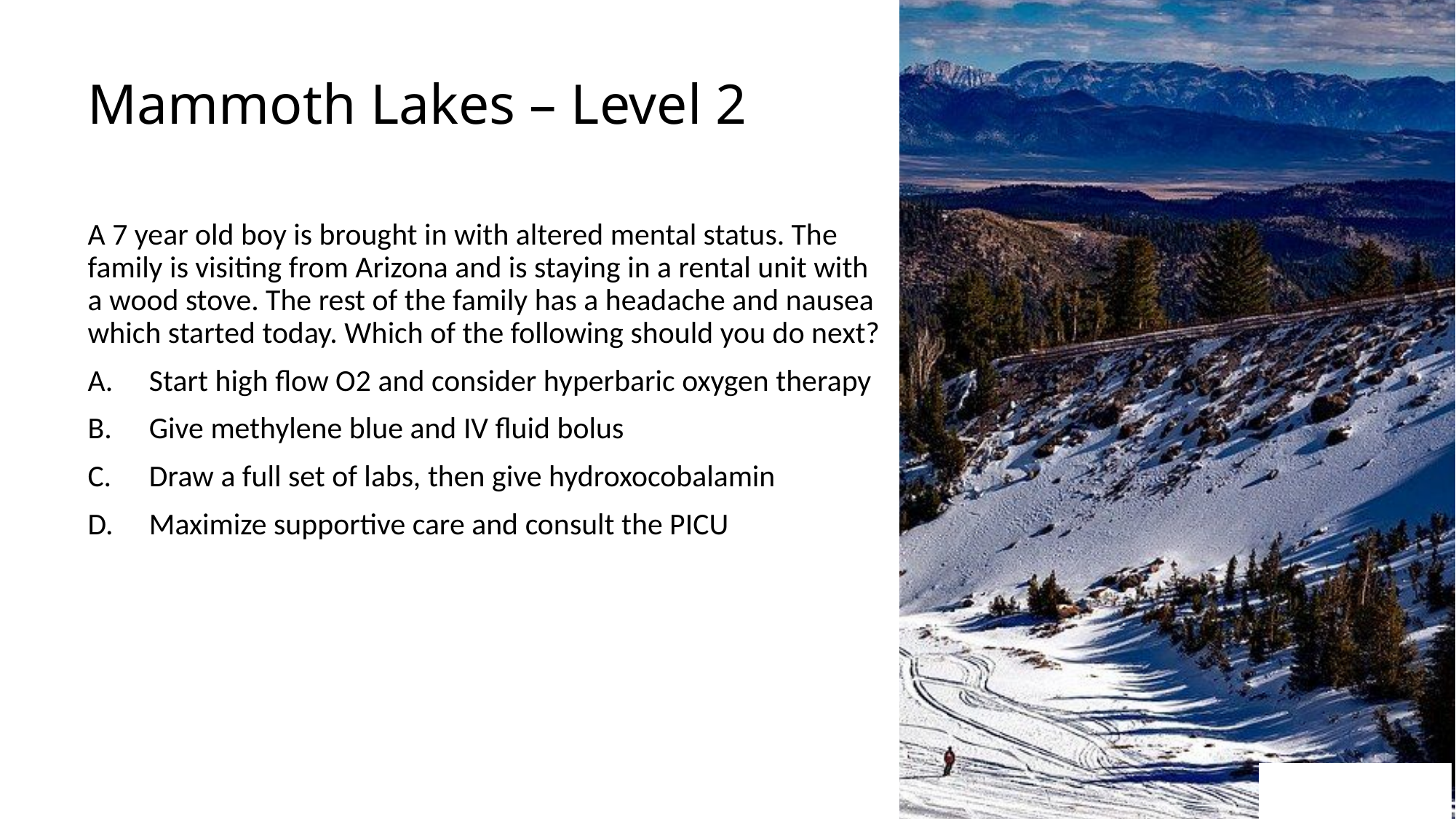

# Mammoth Lakes – Level 2
A 7 year old boy is brought in with altered mental status. The family is visiting from Arizona and is staying in a rental unit with a wood stove. The rest of the family has a headache and nausea which started today. Which of the following should you do next?
Start high flow O2 and consider hyperbaric oxygen therapy
Give methylene blue and IV fluid bolus
Draw a full set of labs, then give hydroxocobalamin
Maximize supportive care and consult the PICU

## Slide 64
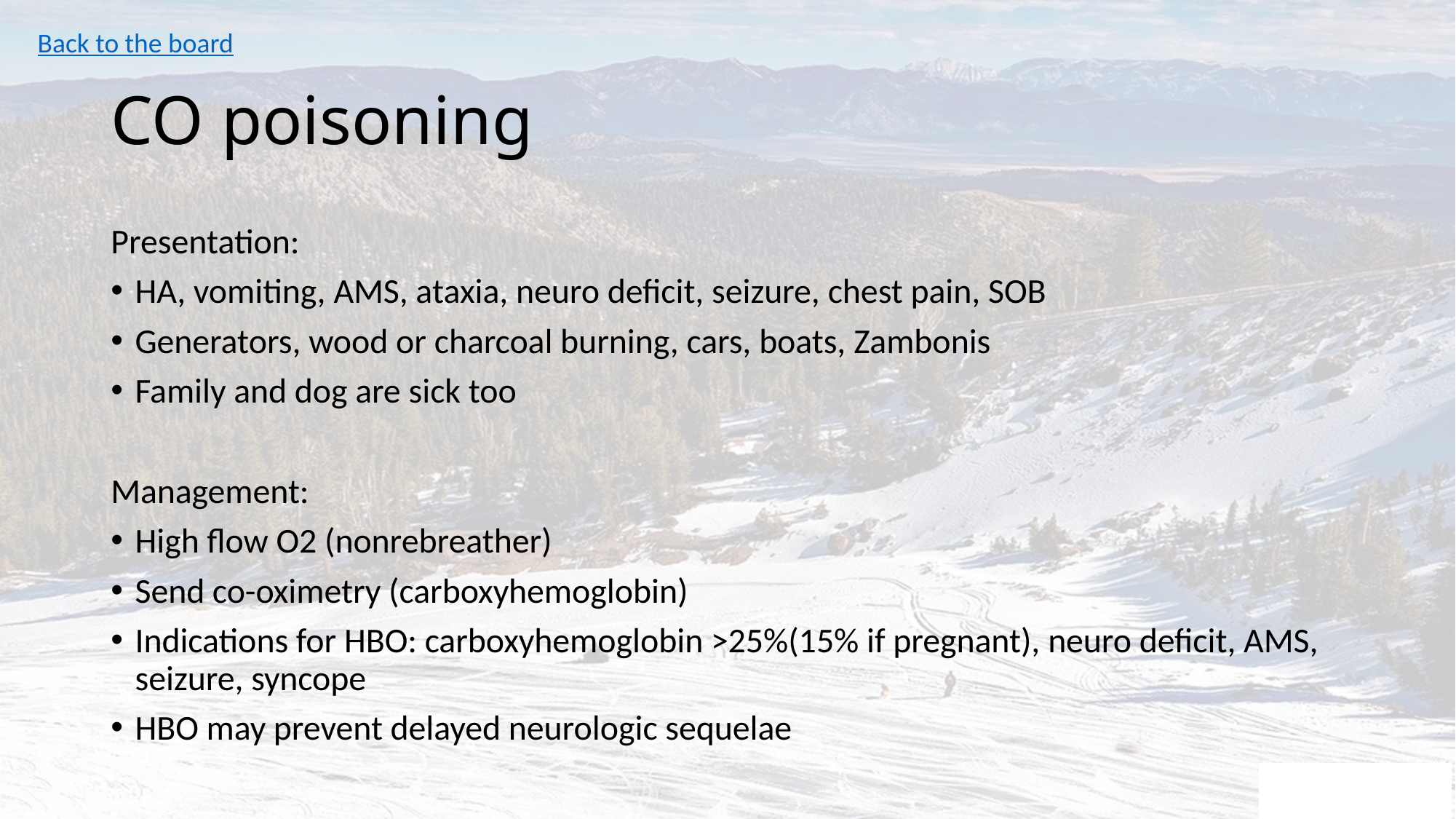

Back to the board
# CO poisoning
Presentation:
HA, vomiting, AMS, ataxia, neuro deficit, seizure, chest pain, SOB
Generators, wood or charcoal burning, cars, boats, Zambonis
Family and dog are sick too
Management:
High flow O2 (nonrebreather)
Send co-oximetry (carboxyhemoglobin)
Indications for HBO: carboxyhemoglobin >25%(15% if pregnant), neuro deficit, AMS, seizure, syncope
HBO may prevent delayed neurologic sequelae

## Slide 65
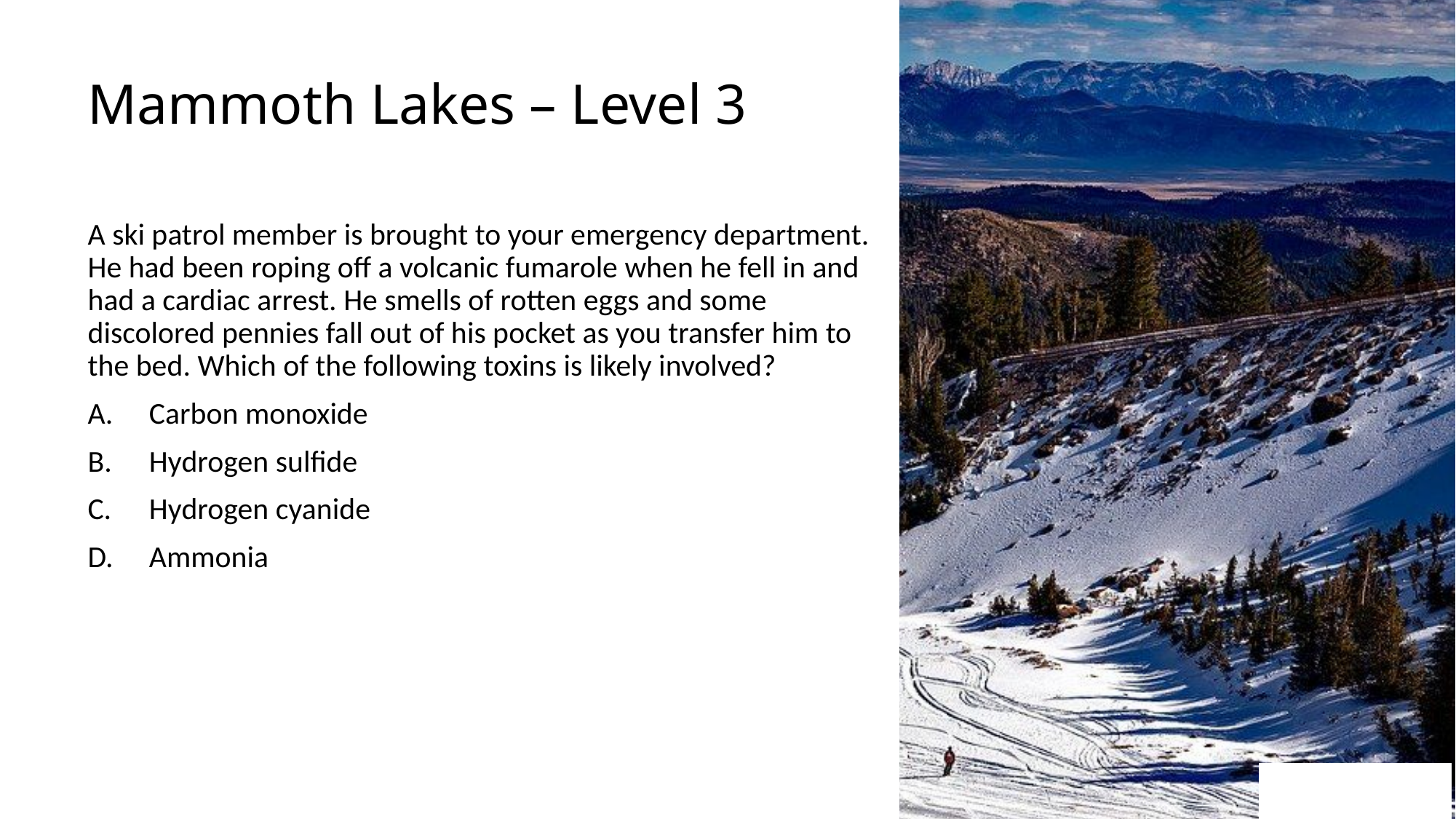

# Mammoth Lakes – Level 3
A ski patrol member is brought to your emergency department. He had been roping off a volcanic fumarole when he fell in and had a cardiac arrest. He smells of rotten eggs and some discolored pennies fall out of his pocket as you transfer him to the bed. Which of the following toxins is likely involved?
Carbon monoxide
Hydrogen sulfide
Hydrogen cyanide
Ammonia

## Slide 66
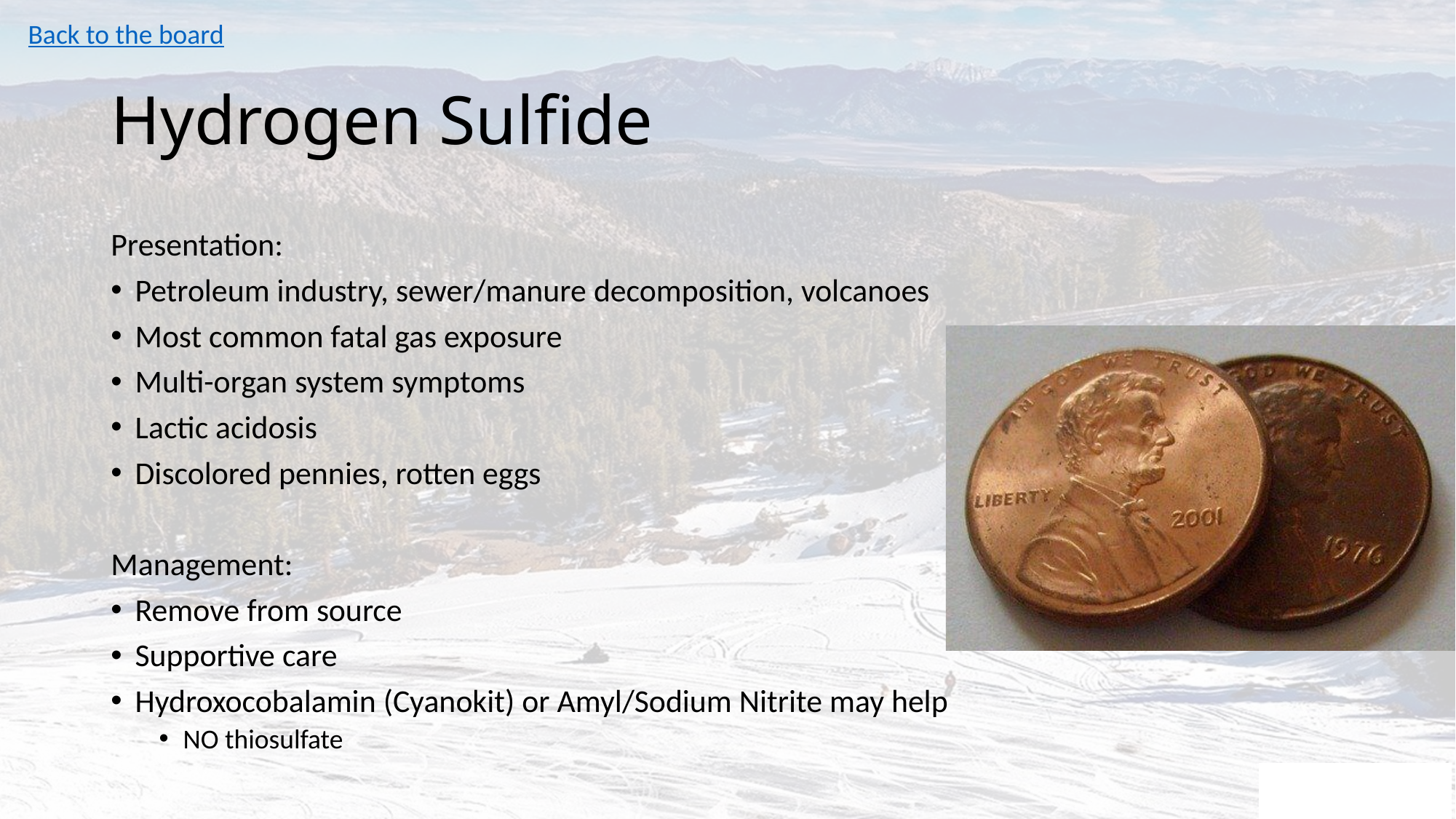

Back to the board
# Hydrogen Sulfide
Presentation:
Petroleum industry, sewer/manure decomposition, volcanoes
Most common fatal gas exposure
Multi-organ system symptoms
Lactic acidosis
Discolored pennies, rotten eggs
Management:
Remove from source
Supportive care
Hydroxocobalamin (Cyanokit) or Amyl/Sodium Nitrite may help
NO thiosulfate

## Slide 67
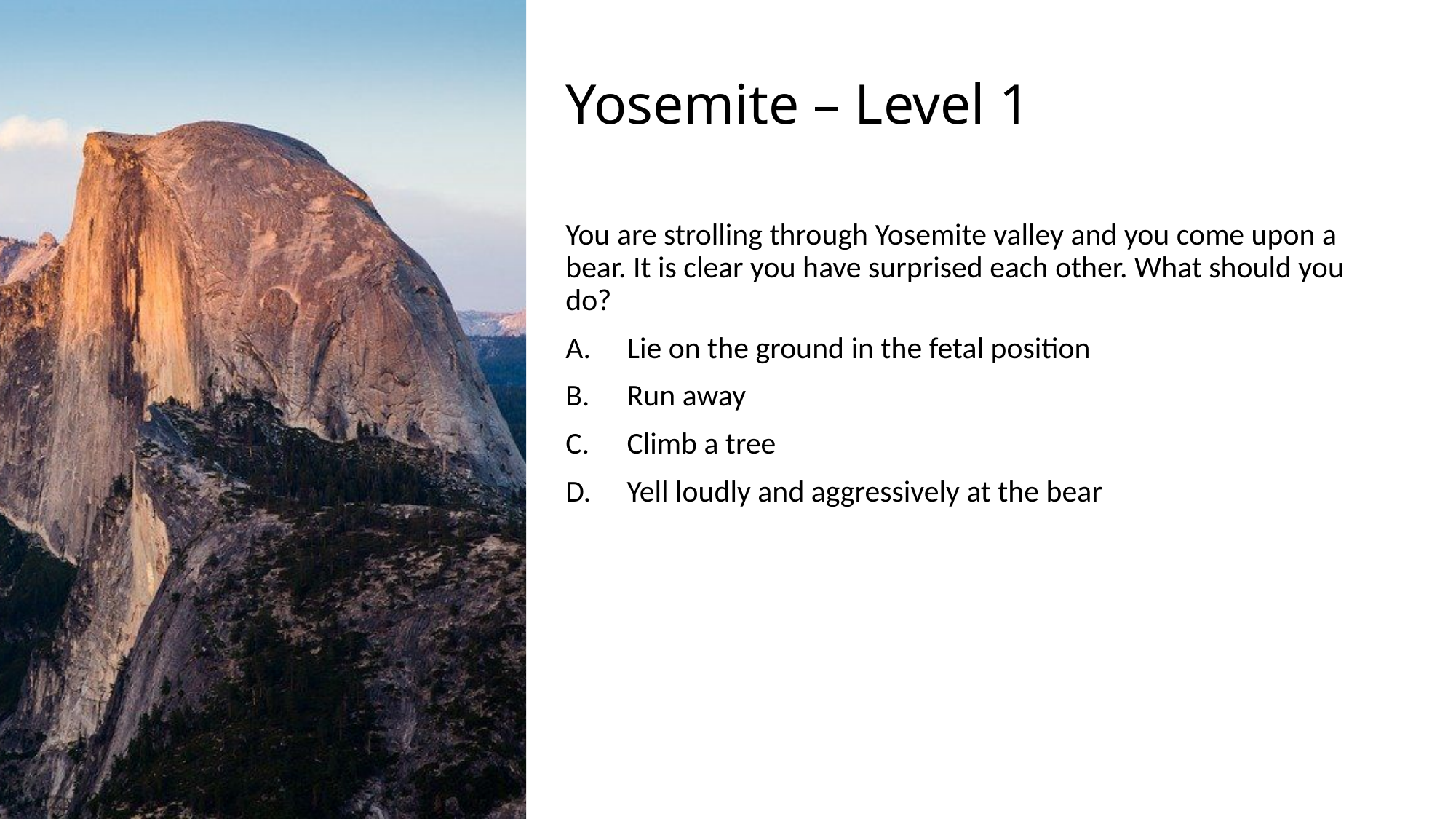

# Yosemite – Level 1
You are strolling through Yosemite valley and you come upon a bear. It is clear you have surprised each other. What should you do?
Lie on the ground in the fetal position
Run away
Climb a tree
Yell loudly and aggressively at the bear

## Slide 68
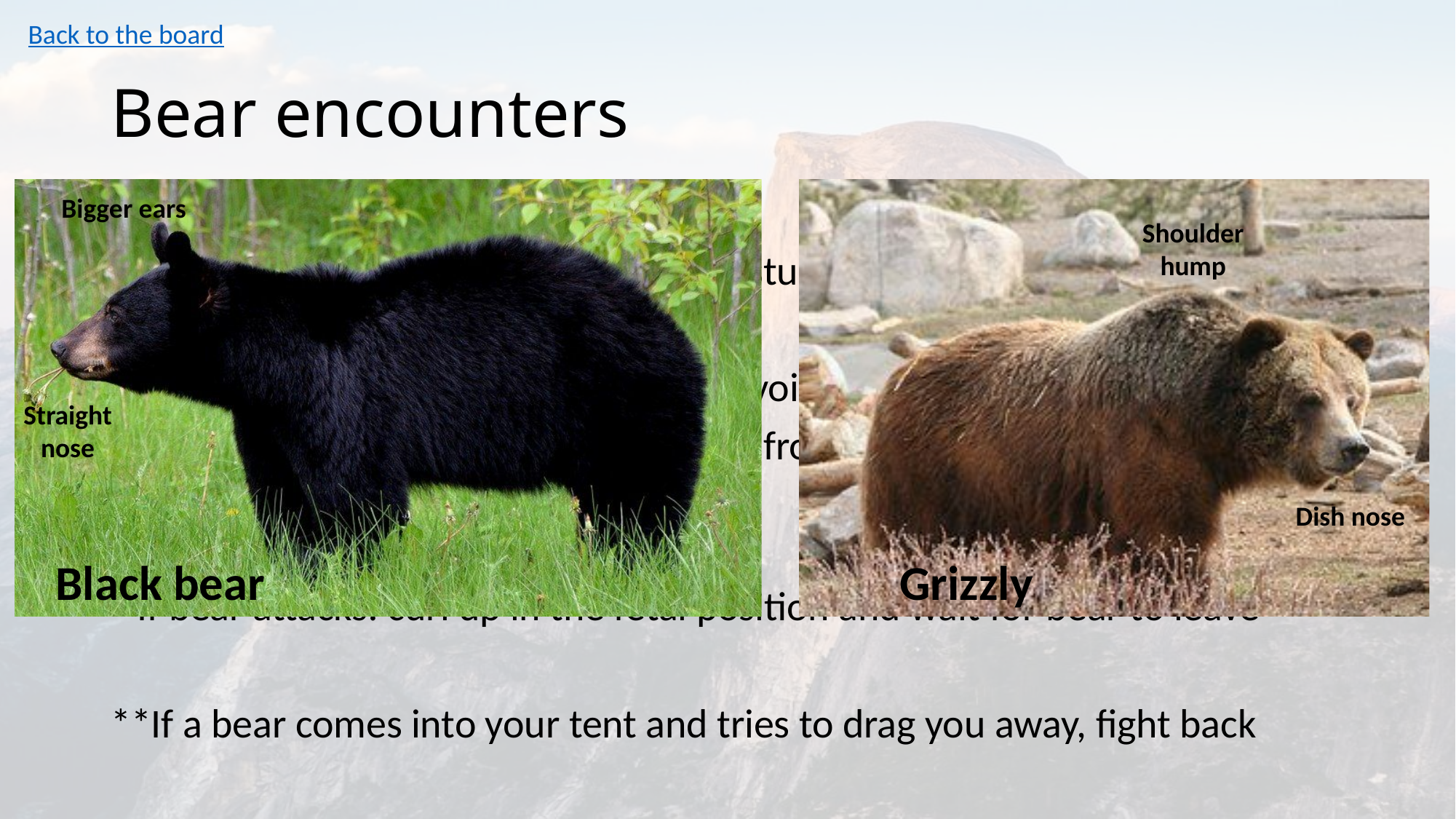

Back to the board
# Bear encounters
Bigger ears
Straight nose
Black bear
Shoulder hump
Dish nose
Grizzly
Presentation
Surprise encounters: you or the bear stumble upon each other
Management
Prevention: make noise on the trail, avoid bear areas
Unknown bear: talk calmly, step away from visual obstruction so bear can fully see you, stand your ground
Black bear: Yell aggressively
If bear attacks: curl up in the fetal position and wait for bear to leave
**If a bear comes into your tent and tries to drag you away, fight back

## Slide 69
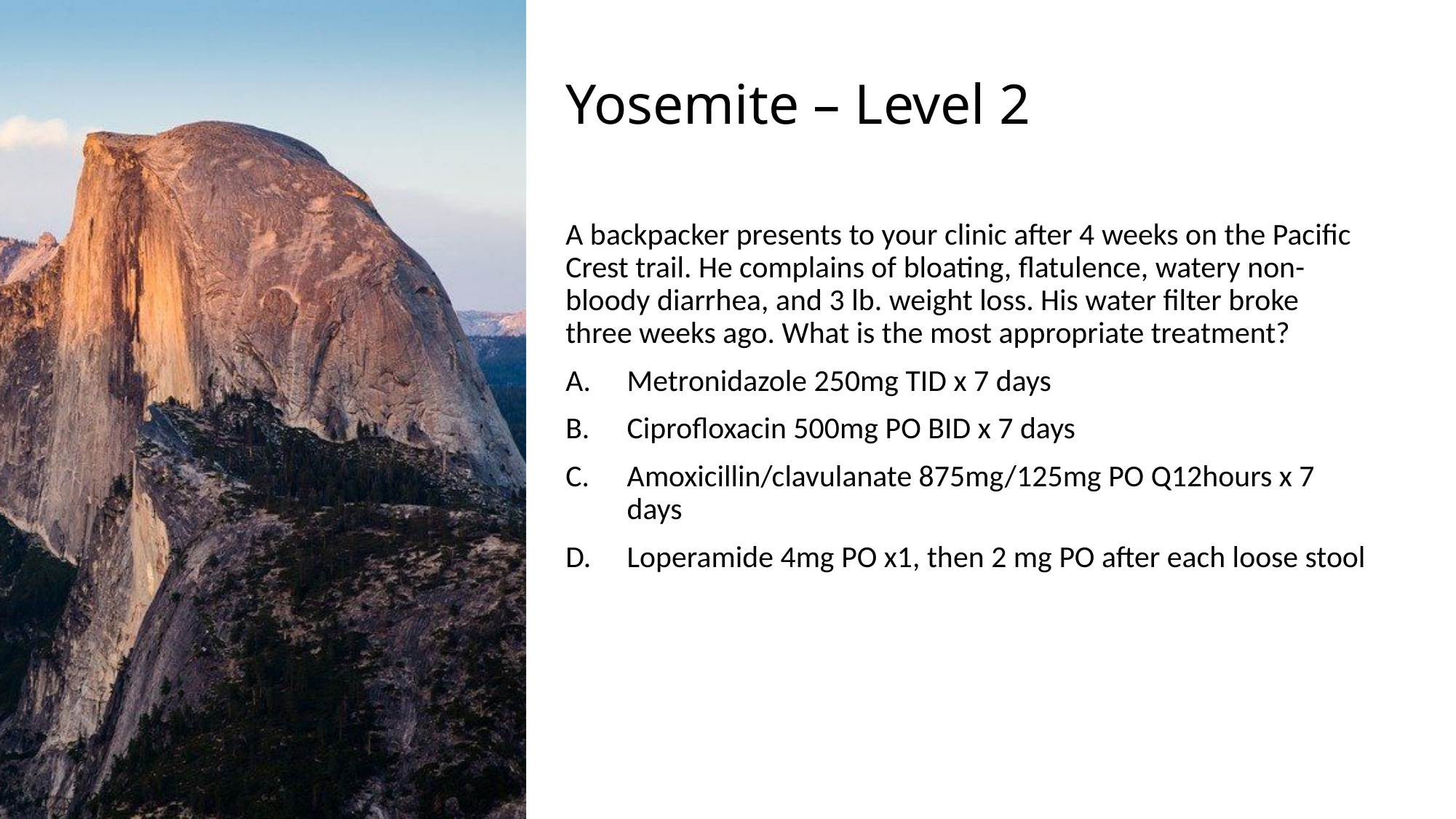

# Yosemite – Level 2
A backpacker presents to your clinic after 4 weeks on the Pacific Crest trail. He complains of bloating, flatulence, watery non-bloody diarrhea, and 3 lb. weight loss. His water filter broke three weeks ago. What is the most appropriate treatment?
Metronidazole 250mg TID x 7 days
Ciprofloxacin 500mg PO BID x 7 days
Amoxicillin/clavulanate 875mg/125mg PO Q12hours x 7 days
Loperamide 4mg PO x1, then 2 mg PO after each loose stool

## Slide 70
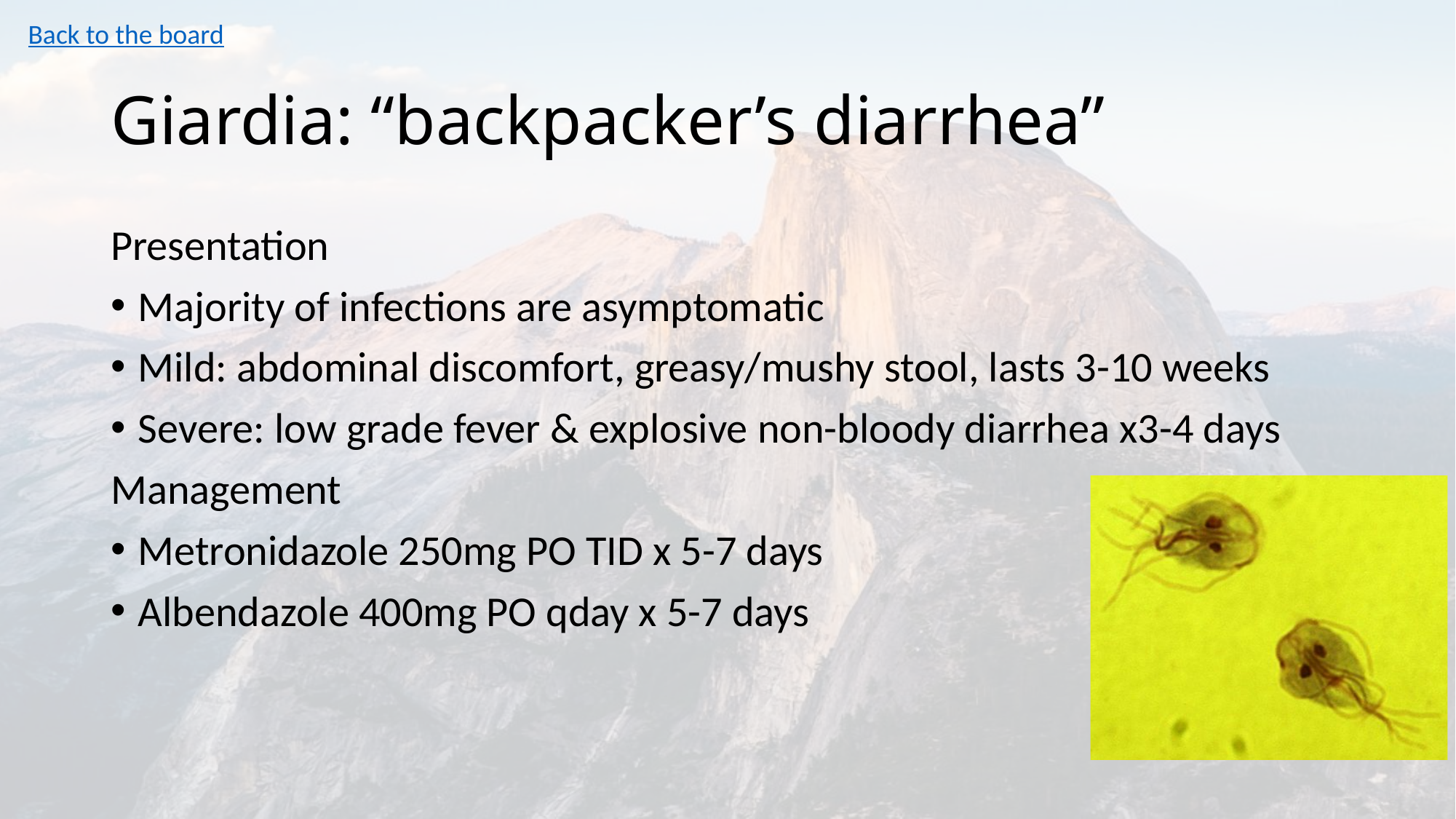

Back to the board
# Giardia: “backpacker’s diarrhea”
Presentation
Majority of infections are asymptomatic
Mild: abdominal discomfort, greasy/mushy stool, lasts 3-10 weeks
Severe: low grade fever & explosive non-bloody diarrhea x3-4 days
Management
Metronidazole 250mg PO TID x 5-7 days
Albendazole 400mg PO qday x 5-7 days

## Slide 71
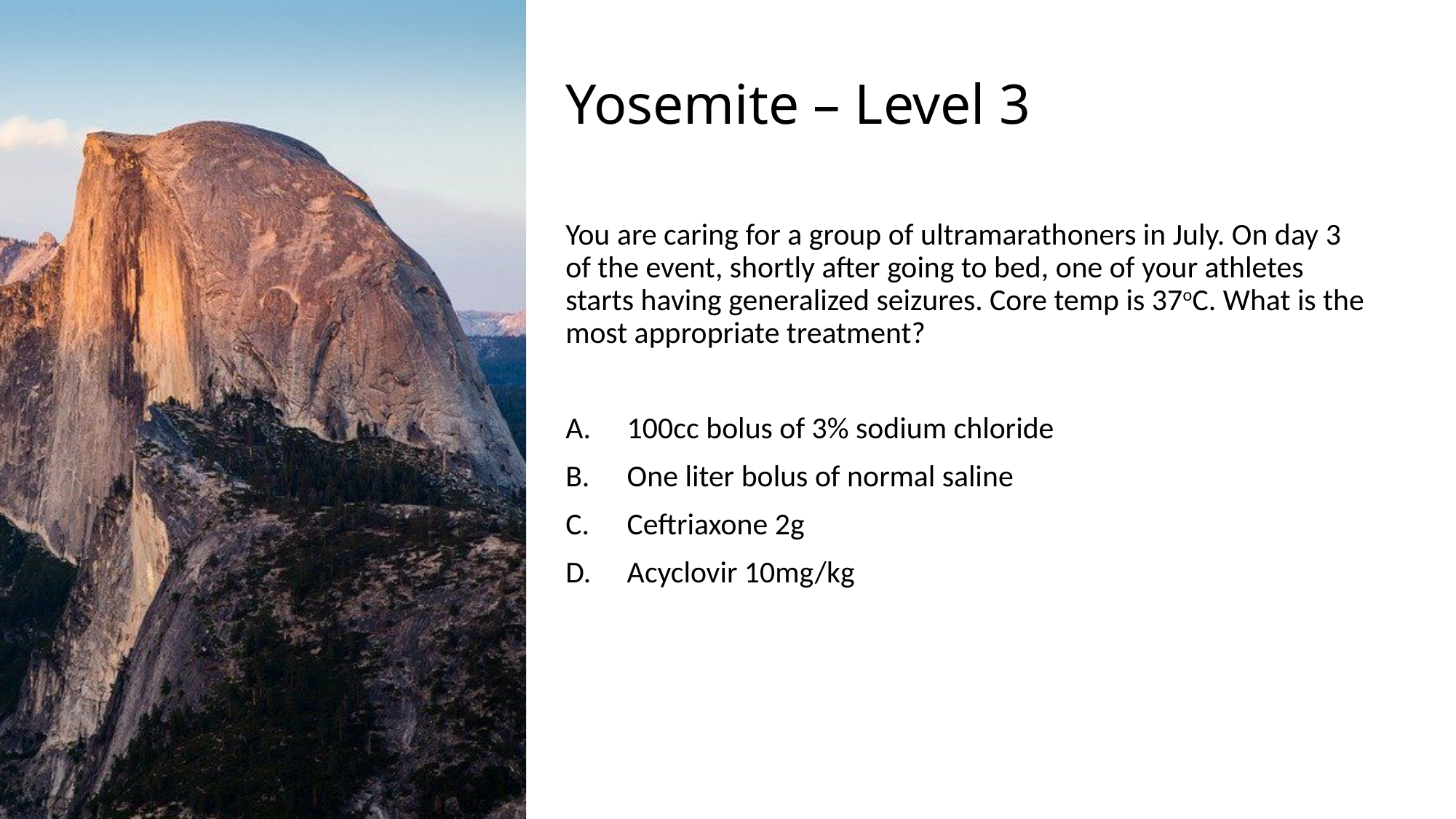

# Yosemite – Level 3
You are caring for a group of ultramarathoners in July. On day 3 of the event, shortly after going to bed, one of your athletes starts having generalized seizures. Core temp is 37oC. What is the most appropriate treatment?
100cc bolus of 3% sodium chloride
One liter bolus of normal saline
Ceftriaxone 2g
Acyclovir 10mg/kg

## Slide 72
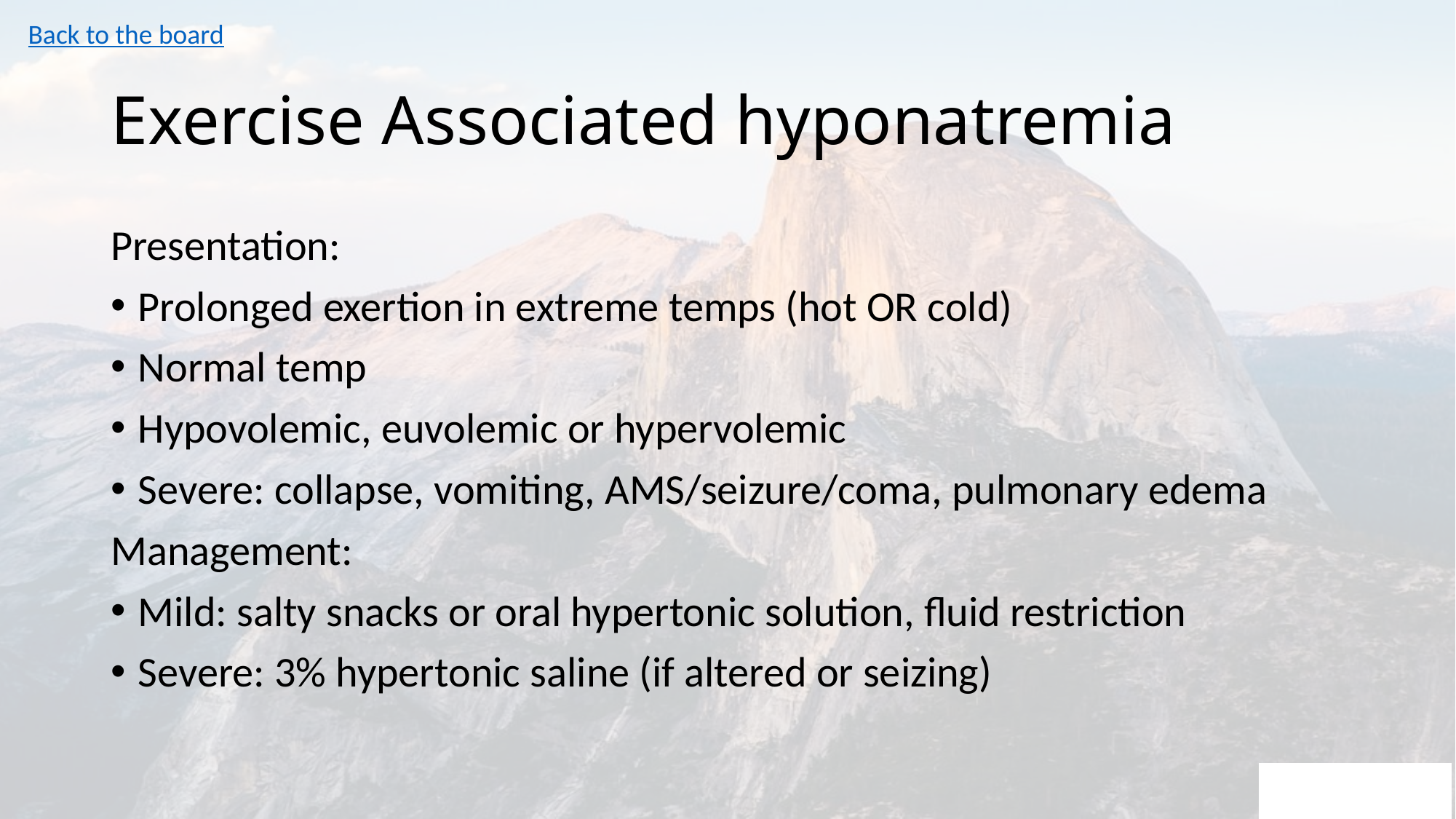

Back to the board
# Exercise Associated hyponatremia
Presentation:
Prolonged exertion in extreme temps (hot OR cold)
Normal temp
Hypovolemic, euvolemic or hypervolemic
Severe: collapse, vomiting, AMS/seizure/coma, pulmonary edema
Management:
Mild: salty snacks or oral hypertonic solution, fluid restriction
Severe: 3% hypertonic saline (if altered or seizing)

## Slide 73
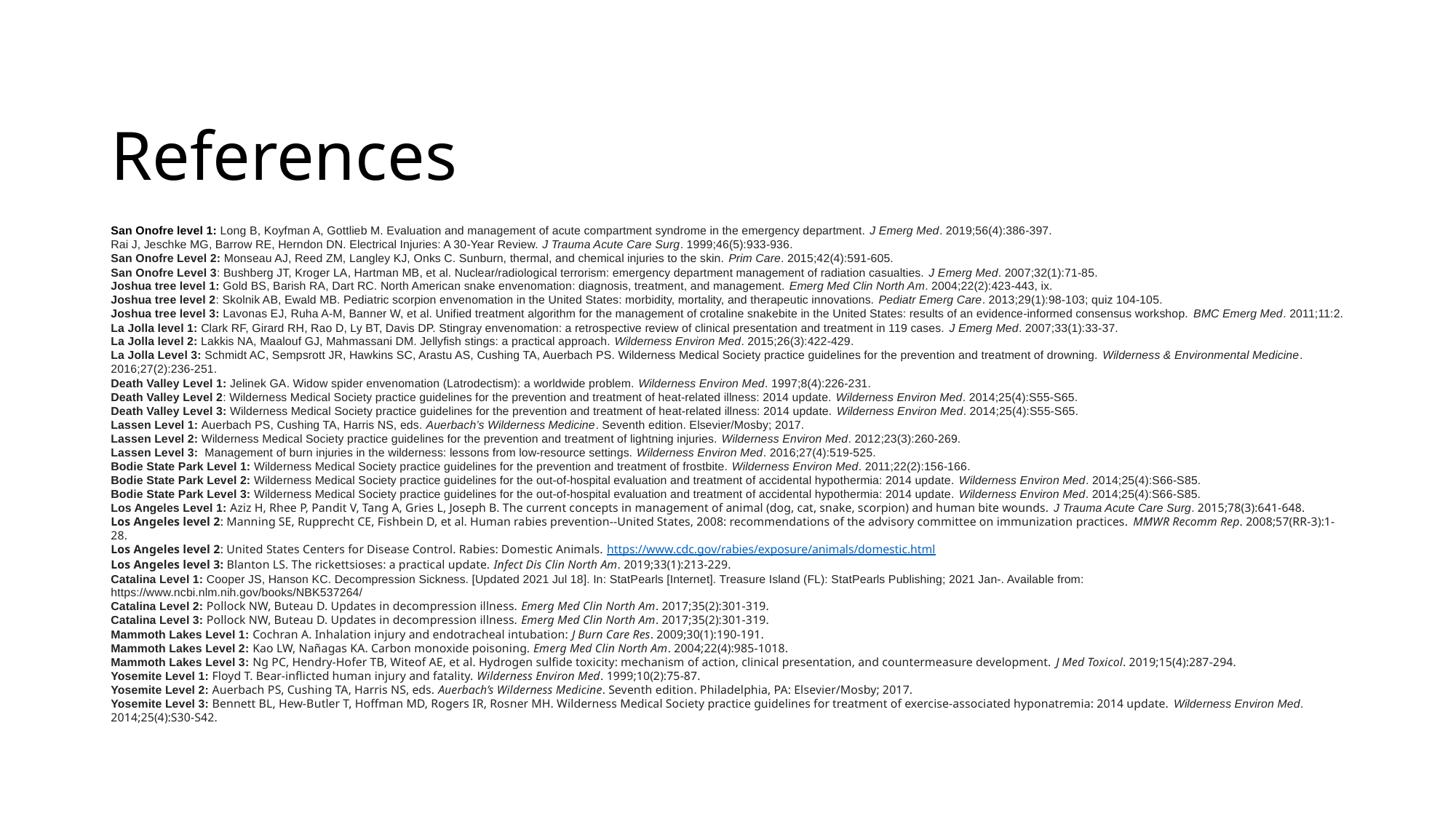

# References
San Onofre level 1: Long B, Koyfman A, Gottlieb M. Evaluation and management of acute compartment syndrome in the emergency department. J Emerg Med. 2019;56(4):386-397.
Rai J, Jeschke MG, Barrow RE, Herndon DN. Electrical Injuries: A 30-Year Review. J Trauma Acute Care Surg. 1999;46(5):933-936.
San Onofre Level 2: Monseau AJ, Reed ZM, Langley KJ, Onks C. Sunburn, thermal, and chemical injuries to the skin. Prim Care. 2015;42(4):591-605.
San Onofre Level 3: Bushberg JT, Kroger LA, Hartman MB, et al. Nuclear/radiological terrorism: emergency department management of radiation casualties. J Emerg Med. 2007;32(1):71-85.
Joshua tree level 1: Gold BS, Barish RA, Dart RC. North American snake envenomation: diagnosis, treatment, and management. Emerg Med Clin North Am. 2004;22(2):423-443, ix.
Joshua tree level 2: Skolnik AB, Ewald MB. Pediatric scorpion envenomation in the United States: morbidity, mortality, and therapeutic innovations. Pediatr Emerg Care. 2013;29(1):98-103; quiz 104-105.
Joshua tree level 3: Lavonas EJ, Ruha A-M, Banner W, et al. Unified treatment algorithm for the management of crotaline snakebite in the United States: results of an evidence-informed consensus workshop. BMC Emerg Med. 2011;11:2.
La Jolla level 1: Clark RF, Girard RH, Rao D, Ly BT, Davis DP. Stingray envenomation: a retrospective review of clinical presentation and treatment in 119 cases. J Emerg Med. 2007;33(1):33-37.
La Jolla level 2: Lakkis NA, Maalouf GJ, Mahmassani DM. Jellyfish stings: a practical approach. Wilderness Environ Med. 2015;26(3):422-429.
La Jolla Level 3: Schmidt AC, Sempsrott JR, Hawkins SC, Arastu AS, Cushing TA, Auerbach PS. Wilderness Medical Society practice guidelines for the prevention and treatment of drowning. Wilderness & Environmental Medicine. 2016;27(2):236-251.
Death Valley Level 1: Jelinek GA. Widow spider envenomation (Latrodectism): a worldwide problem. Wilderness Environ Med. 1997;8(4):226-231.
Death Valley Level 2: Wilderness Medical Society practice guidelines for the prevention and treatment of heat-related illness: 2014 update. Wilderness Environ Med. 2014;25(4):S55-S65.
Death Valley Level 3: Wilderness Medical Society practice guidelines for the prevention and treatment of heat-related illness: 2014 update. Wilderness Environ Med. 2014;25(4):S55-S65.
Lassen Level 1: Auerbach PS, Cushing TA, Harris NS, eds. Auerbach’s Wilderness Medicine. Seventh edition. Elsevier/Mosby; 2017.
Lassen Level 2: Wilderness Medical Society practice guidelines for the prevention and treatment of lightning injuries. Wilderness Environ Med. 2012;23(3):260-269.
Lassen Level 3: Management of burn injuries in the wilderness: lessons from low-resource settings. Wilderness Environ Med. 2016;27(4):519-525.
Bodie State Park Level 1: Wilderness Medical Society practice guidelines for the prevention and treatment of frostbite. Wilderness Environ Med. 2011;22(2):156-166.
Bodie State Park Level 2: Wilderness Medical Society practice guidelines for the out-of-hospital evaluation and treatment of accidental hypothermia: 2014 update. Wilderness Environ Med. 2014;25(4):S66-S85.
Bodie State Park Level 3: Wilderness Medical Society practice guidelines for the out-of-hospital evaluation and treatment of accidental hypothermia: 2014 update. Wilderness Environ Med. 2014;25(4):S66-S85.
Los Angeles Level 1: Aziz H, Rhee P, Pandit V, Tang A, Gries L, Joseph B. The current concepts in management of animal (dog, cat, snake, scorpion) and human bite wounds. J Trauma Acute Care Surg. 2015;78(3):641-648.
Los Angeles level 2: Manning SE, Rupprecht CE, Fishbein D, et al. Human rabies prevention--United States, 2008: recommendations of the advisory committee on immunization practices. MMWR Recomm Rep. 2008;57(RR-3):1-28.
Los Angeles level 2: United States Centers for Disease Control. Rabies: Domestic Animals. https://www.cdc.gov/rabies/exposure/animals/domestic.html
Los Angeles level 3: Blanton LS. The rickettsioses: a practical update. Infect Dis Clin North Am. 2019;33(1):213-229.
Catalina Level 1: Cooper JS, Hanson KC. Decompression Sickness. [Updated 2021 Jul 18]. In: StatPearls [Internet]. Treasure Island (FL): StatPearls Publishing; 2021 Jan-. Available from: https://www.ncbi.nlm.nih.gov/books/NBK537264/
Catalina Level 2: Pollock NW, Buteau D. Updates in decompression illness. Emerg Med Clin North Am. 2017;35(2):301-319.
Catalina Level 3: Pollock NW, Buteau D. Updates in decompression illness. Emerg Med Clin North Am. 2017;35(2):301-319.
Mammoth Lakes Level 1: Cochran A. Inhalation injury and endotracheal intubation: J Burn Care Res. 2009;30(1):190-191.
Mammoth Lakes Level 2: Kao LW, Nañagas KA. Carbon monoxide poisoning. Emerg Med Clin North Am. 2004;22(4):985-1018.
Mammoth Lakes Level 3: Ng PC, Hendry-Hofer TB, Witeof AE, et al. Hydrogen sulfide toxicity: mechanism of action, clinical presentation, and countermeasure development. J Med Toxicol. 2019;15(4):287-294.
Yosemite Level 1: Floyd T. Bear-inflicted human injury and fatality. Wilderness Environ Med. 1999;10(2):75-87.
Yosemite Level 2: Auerbach PS, Cushing TA, Harris NS, eds. Auerbach’s Wilderness Medicine. Seventh edition. Philadelphia, PA: Elsevier/Mosby; 2017.
Yosemite Level 3: Bennett BL, Hew-Butler T, Hoffman MD, Rogers IR, Rosner MH. Wilderness Medical Society practice guidelines for treatment of exercise-associated hyponatremia: 2014 update. Wilderness Environ Med. 2014;25(4):S30-S42.
